# Semisynthetic LC3 Probes for Autophagy Pathways Reveal a Noncanonical LC3 Interacting Region Motif Crucial for the Enzymatic Activity of Human ATG3

**DOI:** 10.1021/acscentsci.3c00009

**Published:** 2023-04-27

**Authors:** Jakob Farnung, Matthias Muhar, Jin Rui Liang, Kateryna A. Tolmachova, Roger M. Benoit, Jacob E. Corn, Jeffrey W. Bode

**Affiliations:** † Laboratory for Organic Chemistry, Department of Chemistry and Applied Biosciences ETH Zürich, CH-8093 Zürich, Switzerland; ‡ Institute of Molecular Health Sciences, Department of Biology ETH Zürich, CH-8093 Zürich, Switzerland; § Laboratory of Nanoscale Biology, Division of Biology and Chemistry, Paul Scherrer Institute, 5232 Villigen PSI, Switzerland

## Abstract

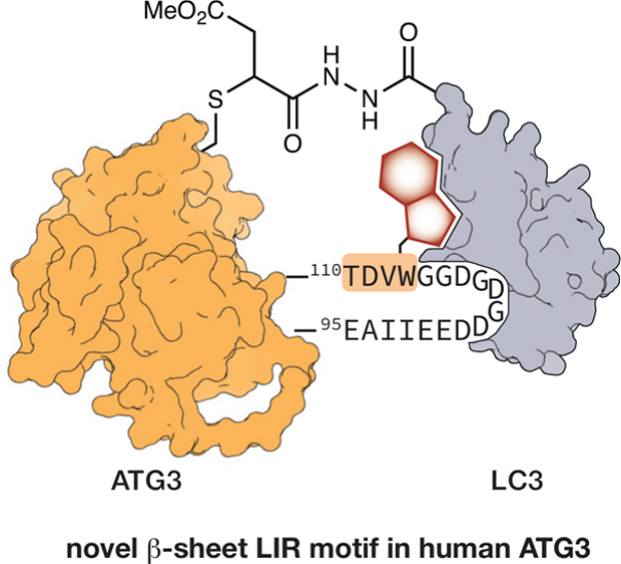

Macroautophagy is one of two major degradation systems in eukaryotic cells. Regulation and control of autophagy are often achieved through the presence of short peptide sequences called LC3 interacting regions (LIR) in autophagy-involved proteins. Using a combination of new protein-derived activity-based probes prepared from recombinant LC3 proteins, along with protein modeling and X-ray crystallography of the ATG3-LIR peptide complex, we identified a noncanonical LIR motif in the human E2 enzyme responsible for LC3 lipidation, ATG3. The LIR motif is present in the flexible region of ATG3 and adopts an uncommon β-sheet structure binding to the backside of LC3. We show that the β-sheet conformation is crucial for its interaction with LC3 and used this insight to design synthetic macrocyclic peptide-binders to ATG3. CRISPR-enabled *in cellulo* studies provide evidence that LIR^ATG3^ is required for LC3 lipidation and ATG3∼LC3 thioester formation. Removal of LIR^ATG3^ negatively impacts the rate of thioester transfer from ATG7 to ATG3.

## Abstract 2

A previously unknown LIR motif in human ATG3 was identified using a combination of activity-based probes and protein modeling. The LIR motif adopts an unusual β-sheet conformation and is crucial for the enzymatic activity of ATG3.

## Introduction

Macroautophagy (herein referred to as autophagy) is a major catabolic process in eukaryotic cells,^1^ responsible for the bulk degradation of various cellular components such as proteins,^2^ cellular compartments,^3^ and pathogens.^4^ Akin to the ubiquitin proteasome system, two families of small protein modifiers are crucial regulatory elements of autophagy. Proteins of the LC3 or GABARAP families are conjugated via their C-terminal glycine residue to phosphatidylethanolamine-containing lipids.^5^ This process is catalyzed by an intricate enzymatic cascade in which ATG7 functions as an activating E1 enzyme using ATP to form a thioester with LC3/GABARAP and transferring them to an E2 enzyme, ATG3, via a trans-thioesterification reaction. ATG3 performs the lipidation of LC3/GABARAP in conjunction with an E3-like enzyme complex of isopeptide-linked ATG5-ATG12. Membrane tethering of LC3/GABARAP is crucial for membrane expansion of autophagosomes to engulf the autophagic cargo and for eventual lysosome fusion.^6−9^ However, the mechanistic details of LC3/GABARAP-lipidation by ATG3 remain enigmatic.

A conserved feature of the autophagy pathway is the recurring presence of short peptide motifs called LC3 interacting regions (LIR) in proteins associated with autophagy.^10^ Proteins containing LIR motifs are recruited to LC3/GABARAP through hydrophobic interactions. The core sequence of LIR motifs, ΦxxΨ, is characterized by the presence of an aromatic residue (Φ)—Trp, Phe, and Tyr—followed by two variable positions and an aliphatic, hydrophobic amino acid (Ψ), generally Ile, Leu, or Val.^11^ The LIR motif adopts an extended conformation forming an intermolecular β-sheet with β2 of LC3/GABARAP. A variety of LIR motifs have been identified in selective autophagy receptors, which employ them to recruit cargos to expanding autophagosomes.^2^ In addition, these motifs can also be found in proteins involved in the attachment of LC3/GABARAP to membranes such as ATG4,^12^ a protease required for processing of proLC3/proGABARAP and delipidation of LC3/GABARAP.

Few chemical probes for autophagy have been developed.^13−15^ The majority are inhibitors of autophagy proteins functioning upstream of the lipidation cascade, such as wortmannin, a PI3K inhibitor that abrogates localization of lipidation enzymes to expanding autophagosomes.^16,17^ Despite the similarity to the ubiquitin proteasome pathway, few chemical probes exist for LC3/GABARAP lipidation.^18,19^ Hemelaar et al. reported the synthesis of LC3/GABARAP activity-based probes by direct aminolysis and employed these probes to identify ATG4 as the processing protease of proLC3/proGABARAP.^20^ However, access to these probes by direct aminolysis is generally hampered by harsh reaction conditions and inefficient conversion.

Herein, we report the facile preparation of GABARAP and LC3A activity-based probes (ABPs) using a recently established hydrazide acylation protocol.^21,22^ Access to these ABPs was critical for the identification of an unknown noncanonical LIR motif embedded in an unusual β-sheet conformation in human ATG3. Further investigation of this motif with macrocyclic peptide binders, X-ray crystallography, and CRISPR-enabled *in cellulo* studies revealed that this LIR motif is crucial for the enzymatic function of ATG3.

## Results and Discussion

### Preparation of GABARAP and LC3A Activity-Based Probes

**Figure 1:**
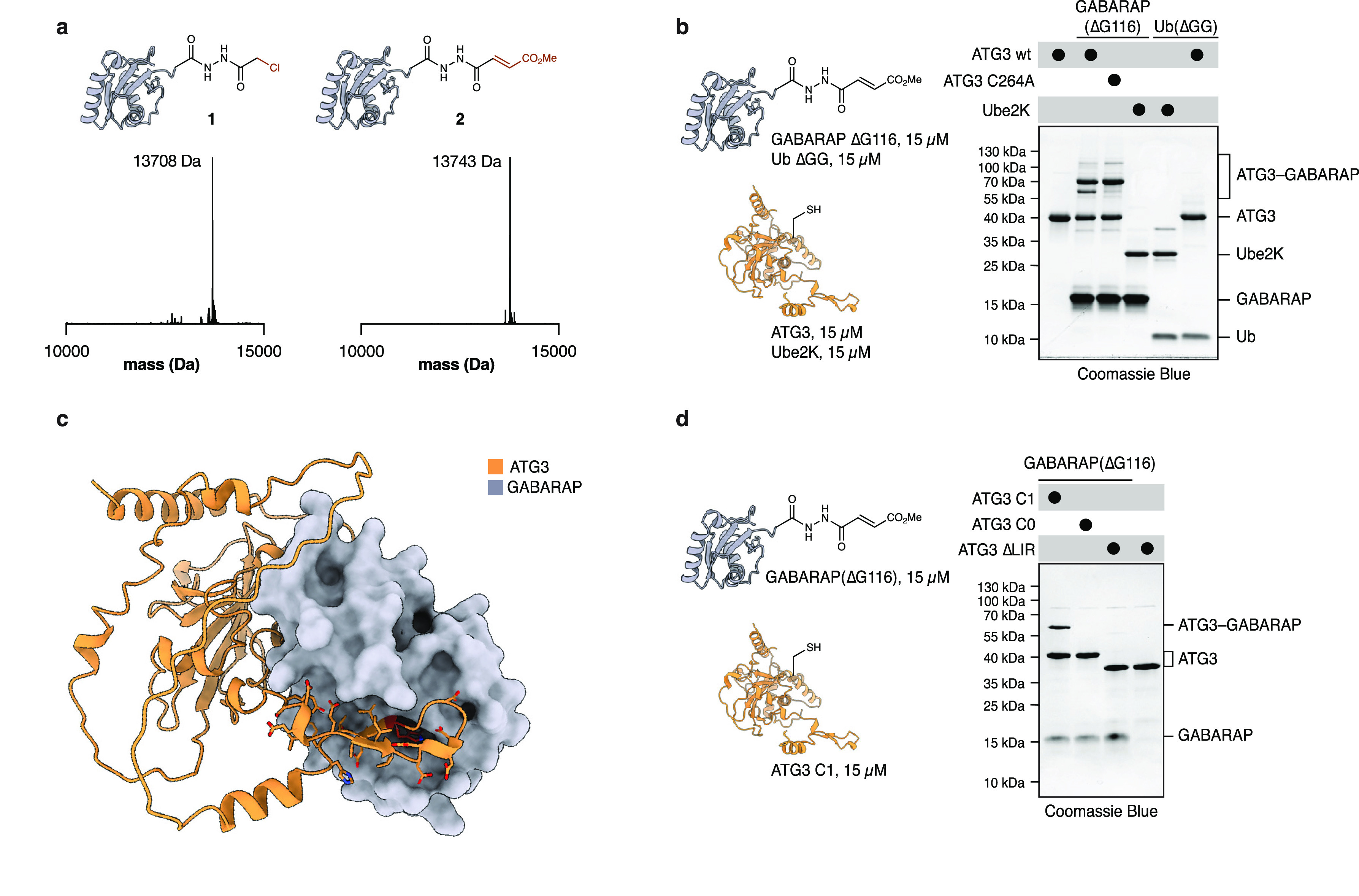
Modification of ATG3 with GABARAP ABPs depends on LIR^ATG3^. **a**. Characterization of GABARAP activity-based probes, **1** and **2**, obtained by hydrazide-acylation. Deconvoluted mass-spectrum (ESI) of GABARAP(ΔG116)–NHNH α-chloroacetyl **1**. Expected mass 13707 Da. Deconvoluted mass-spectrum (ESI) of GABARAP(ΔG116)–NHNH methyl-fumarate **2**. Expected mass 13743 Da. **b**. Reaction of **2** or Ub(ΔGG)–NHNH methyl fumarate probe (15 μM) with recombinant E2s ATG3 and Ube2K (15 μM). The reaction was allowed to proceed for 1 h and was analyzed by SDS-PAGE and Coomassie Blue staining. **c**. ColabFold-predicted structure of the protein-complex of ATG3 and GABARAP. Side-chain atoms are shown for the predicted LIR motif in ATG3 (90–112). **d**. Reaction of **2** (15 μM) with ATG3 variants C1, C0, and ΔLIR (15 μM). ATG3 C1 contains only active-site cysteine C264. ATG3 C0 contains no cysteines. ATG3 ΔLIR lacks amino acids 95–111. Reaction was allowed to proceed for 1 h and was analyzed by SDS-PAGE and Coomassie Blue staining. Full-gel images for **b** and **d** are available in the Supplementary Information.

We recently reported access to Ubl proteins bearing thiol-reactive electrophiles at their C-terminus by chemoselective acylation of recombinant protein-hydrazides with carboxylic acid anhydrides.^22^ The lack of ABPs for autophagy inspired us to prepare GABARAP ABPs using this protocol. GABARAP was expressed as an *Mxe* GyrA intein fusion to C-terminal glycine deletion (ΔG116) to conserve the atomic register at the C-terminus after the introduction of electrophilic groups. The expressed fusion proteins were cleaved with hydrazine and chemoselectively acylated with symmetric anhydrides at pH 3.0. The resulting acylhydrazides mimic the native glycine and place the electrophile close to the reactive site of the native C-terminus (Figure S1). In analogy to probes that showed excellent activity in the UFM1-pathway, we selected α-chloroacetyl probe **1** and methyl fumarate probe **2** derived from GABARAP(ΔG116)-NHNH_2_ for further studies into autophagy pathways (Figure 1a).^21^

To assess the reactivity of the probes, we incubated **1** and **2** (15 μM) with recombinantly expressed ATG3 (15 μM) and its catalytically inactive variant C264A (Figure 1b, Figure S2). Probe **2** reacted with ATG3 efficiently leading to the formation of multiple ATG3–GABARAP bands. Mutation of active-site cysteine 264 to alanine caused the loss of one ATG3–GABARAP band but did not abrogate additional bands for the complex, indicating unspecific modification of ATG3, likely due to the presence of multiple cysteine residues in flexible regions of ATG3. Probe **2** nonetheless reacted specifically with its cognate E2 ATG3, as ubiquitin E2 Ube2K did not react with **2** and ATG3 wt did not react with Ub(ΔGG) methyl fumarate probe. We were intrigued by the cross-linking efficiency of **2** with ATG3 and sought to investigate the origin of this efficiency by generating a C1 variant of ATG3 that only contains the catalytic cysteine 264 and a corresponding C0 variant containing no cysteines. Probe **2** showed excellent reactivity with ATG3 C1, and as expected, no reaction was observed with ATG3 C0 (Figure 1d, lanes 1–2). ATG3 C1 maintains its cross-linking efficiency compared to ATG3 wt indicating that cross-linking does not arise simply from the presence of numerous cysteine residues in ATG3 but rather from enhanced affinity between ATG3 and GABARAP. In general, the affinity of E2s for their cognate Ubls are quite low, as the thioester transfer from the E1 to the E2 enzyme is facilitated by additional interactions from the UFD domain of the E1 enzyme.^23^ The specificity and high reactivity of our probes with human ATG3 indicated that ATG3 may contain additional binding elements, resulting in a tighter interaction than generally observed for E2s and their Ubl cognates.^24^

### Human ATG3 Contains a Noncanonical LIR Motif

Previous reports established that yeast ATG3 contains an ATG8 interaction motif required for interaction with ATG8, the yeast analogue of LC3 and GABARAP.^25^ However, this motif is not conserved in human ATG3 and therefore did not explain the enhanced interaction of human ATG3 with GABARAP (Figure S3). As there was no structural information available on human ATG3 that could explain the higher affinity of ATG3 for GABARAP, we turned to computational methods. Artificial intelligence driven modeling such as AlphaFold has shown great promise in accessing structural data on proteins for which no structural data is available.^26^ Recent improvements have enabled researchers to model protein–protein interactions,^27^ and we employed an open-source modeling tool, ColabFold, to model the protein–protein interaction between ATG3 and GABARAP (Figure 1c, Figures S4–S5).^28^ Intriguingly, ColabFold modeled a complex of ATG3 and GABARAP that resembled the canonical closed-conformation observed in E2-Ub thioester complexes.^29,30^ Additionally, a section of the flexible region of ATG3, L94-Y111, folded into a short β-sheet that was bound to a groove formed by helices α2 and α3. The interaction site on GABARAP was equivalent to the canonical binding region of LIR motifs. Inspection of the β-sheet sequence shows a sequence motif, W^107^VDT^110^, reminiscent of core LIR motifs but containing threonine instead of the canonical Ile, Leu, or Val residues. The binding mode of the WDVT motif was similar to binding modes observed for previously investigated LIR motifs; W107 binds to hydrophobic pocket (HP) 1 and T110 to HP2. Therefore, the WVDT motif likely represents a noncanonical LIR motif previously undiscovered in human ATG3, in which T110 binds to GABARAP instead of the canonical aliphatic amino acids. The β-sheet forms further interactions with GABARAP outside of the core LIR motif. Several ionic interactions between Asp and Glu residues in the peptide and Lys/Arg residues surrounding the binding site seem to further stabilize the interaction.

Sequence alignment of ATG3 protein sequences from different organisms shows that the LIR motif described herein is conserved across species and kingdoms (Figures S6, S7). Intriguingly, several species contain two LIR motifs, an LIR motif analogous to the motif found in *S. cerevisiae* and a second LIR motif analogous to the LIR motif found in human ATG3 described herein. In contrast, animals and plants only encode for one LIR motif corresponding to the human LIR motif.^31^*S. cerevisiae* and the closely related *S. pastorianus* were the only analyzed species not containing an analogue of the human LIR motif. Alignment of the predicted LIR motif shows strict conservation of tryptophan and preference for threonine. Surprisingly, the second conserved residue, occupied by threonine in humans, is almost exclusively populated by residues not considered canonical for LIR motifs (Figure S8).

LIR^ATG3^ was predicted to be embedded in a β-sheet, a conformation rarely found in reported LIR motifs. Most LIR motifs are embedded in an extended conformation that forms an intermolecular β-sheet with β2 of LC3/GABARAP. The presence of LIR-motifs within β-sheets has only recently been reported for the pathogenic protein RavZ,^32^ FNIP,^33^ and TP53INP2^34^ (vide infra). However, several other noncanonical LIR motifs have been reported. For example, UBA5 binds GABARAP using two aliphatic residues and an additional aromatic amino acid outside of the core LIR sequence,^35^ and NDP52 exclusively binds to LC3C via its cLIR motif consisting of only aliphatic residues.^36^ Nevertheless, even these noncanonical motifs bind in an extended linear fashion to LC3/GABARAP.

We hypothesized that the putative noncanonical LIR motif in ATG3 was responsible for its high affinity interaction with GABARAP, resulting in the efficient reaction of our probes with ATG3. We sought to test this with a cross-linking assay and chose ATG3 variants C1 and C0 (vide supra) to facilitate analysis. We expressed a variant of ATG3 C1 lacking the LIR motif (Δ95–111), ΔLIR. Incubation of **2** with ATG3 ΔLIR showed no complex formation by SDS-PAGE analysis (Figure 1d). Removal of the LIR motif by deletion of residues 95–111 had the same effect as removal of the catalytic cysteine C264. The same LIR-dependence was observed for cross-linking with wild-type ATG3, which retained all its cysteine residues (Figure S9). This observation was independent of the probe used; reaction of probe **1** and cross-linking with ATG3 were also strictly dependent on LIR^ATG3^. These findings support the ColabFold model that ATG3 contains an additional binding element for GABARAP through its LIR motif.

GABARAP is representative of one of two protein families conjugated by ATG3. The LC3-family is also tethered to membranes by ATG3. To test the general involvement of LIR^ATG3^ in ATG3 activity beyond GABARAP we also prepared LC3A probes **3** and **4**. We allowed these probes to react with ATG3 C1, C0, and ΔLIR. As observed for GABARAP, LC3A shows efficient reaction with ATG3 C1 but no reaction with either C0 or ΔLIR variants (Figure S10). These results indicate that LIR^ATG3^ has a general role in ATG3 binding to LC3/GABARAP.

### LIR^ATG3^ Motif Forms a β-Sheet

**Figure 2:**
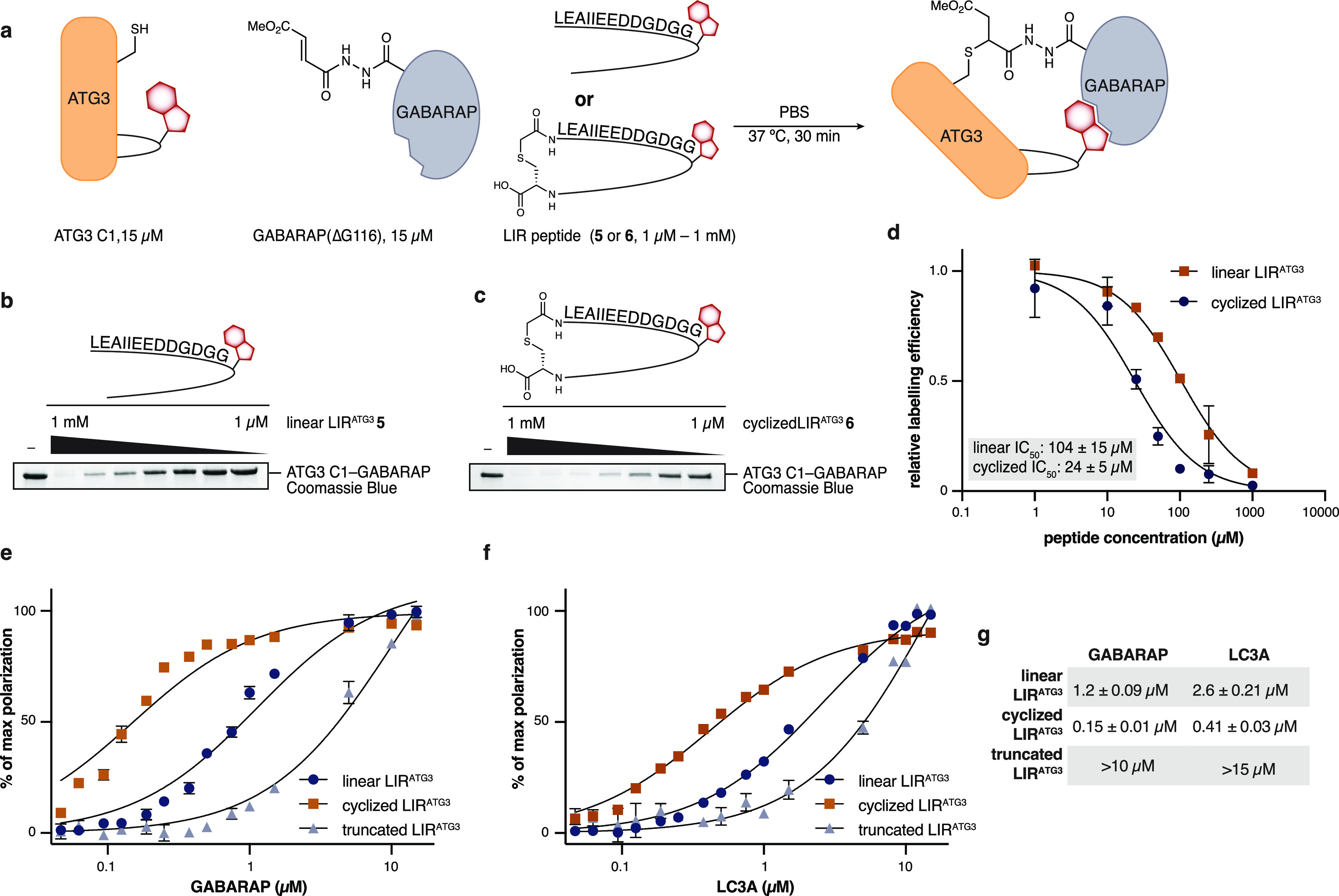
Characterization of LIR^ATG3^. **a**. Reaction scheme outlining competition assay used for **b**, **c**, and **d**. **b**. Coomassie Blue-stained SDS-PAGE analysis for reaction of **2** with ATG3 C1 in the presence of varying concentrations of linear LIR^ATG3^ peptide **5** as outlined in **a**. **c**. Coomassie Blue-stained SDS-PAGE analysis for reaction of **2** with ATG3 C1 in the presence of varying concentrations of cyclized LIR^ATG3^ peptide **6** as outlined in **a**. **d**. Quantification of competition assays shown in **b** and **c**. The amount of GABARAP–ATG3 C1 complex was quantified by gel-densitometry and normalized to the reaction without peptide for each gel. IC_50_ was estimated by nonlinear regression. *n* = 2,3 independent experiments with similar results. Data are presented as average values ± s.d. **e**. Fluorescence polarization binding-data of fluorescein-modified LIR^ATG3^ peptide binding to GABARAP. Either linear, cyclized, or truncated (103–111) LIR^ATG3^ peptide was used. The measurement was performed in triplicates and data shown as average values ± s.d. *K*_D_ was estimated using nonlinear regression. **f**. Fluorescence polarization data of fluorescein-modified LIR^ATG3^ peptide binding to LC3A. Either linear, cyclized, or truncated (103–111) LIR^ATG3^ peptide was used. *K*_D_ was estimated using nonlinear regression. The measurement was performed in triplicate and data shown as average values ± s.d. **g**. Table summarizing *K*_D_ values obtained in **e**,**f**. Full-gel images for **b** and **c** are available in the Supplementary Information.

To exclude an effect of LIR deletion on protein activity we performed a competition experiment with chemically prepared LIR (L94-H112) peptide **5**. Probe **2** (15 μM) was incubated with ATG3 C1 in the presence of increasing amounts of **5**. The peptide blocked ATG3 modification in a concentration-dependent manner with an IC_50_ of 104 μM, indicating that the LIR motif is involved in binding GABARAP (Figure 2a,b,d). We also postulated that cyclization of the LIR peptide would recapitulate its β-sheet conformation and lead to tighter binding than the linear peptide due to preorganization.^37^ This approach has recently been shown to be successful in the *de novo* design of GABARAP-specific peptide binders.^38^ Cyclic peptide **6** was prepared by solid-phase peptide synthesis followed by cyclization using selective cysteine alkylation (Figure S11).^39,40^ Peptide **6** inhibited the reaction of **2** with ATG3 C1 with an IC_50_ of 24 μM (Figure 2c,d). This is 5-fold lower than the IC_50_ observed for the linear peptide **5,** suggesting that the β-sheet conformation is indeed crucial for the interaction of the ATG3 LIR motif with GABARAP. CD spectroscopic analysis of the peptides showed that linear peptide **5** is not structured in solution. Upon cyclization **6** remains mostly unstructured with some transition to an organized structure (Figure S12). Additionally, we confirmed the interaction of LIR^ATG3^ peptides with GABARAP and LC3A by fluorescence polarization (Figure 2e–g). LIR^ATG3^-derived peptides bind to both LC3A and GABARAP. As indicated by the competition experiments, cyclic peptides bound markedly tighter to GABARAP and LC3A than the linear peptides. Tighter binding upon peptide cyclization strongly suggests that it is the β-sheet conformation, observed in our prediction, that binds to GABARAP/LC3A because conformational restriction of the peptide precludes binding to other regions of GABARAP in an extended fashion. A truncated peptide, containing amino acids G103-Y111 including the core LIR motif, bound 6-fold weaker than the linear peptide. The severely diminished affinity observed for the truncated peptide indicates that the solvent-exposed upper half of the β-sheet (104–95) contributes significantly to the binding of LIR^ATG3^.

### Cocrystal Structure of GABARAP and LIR^ATG3^

**Figure 3:**
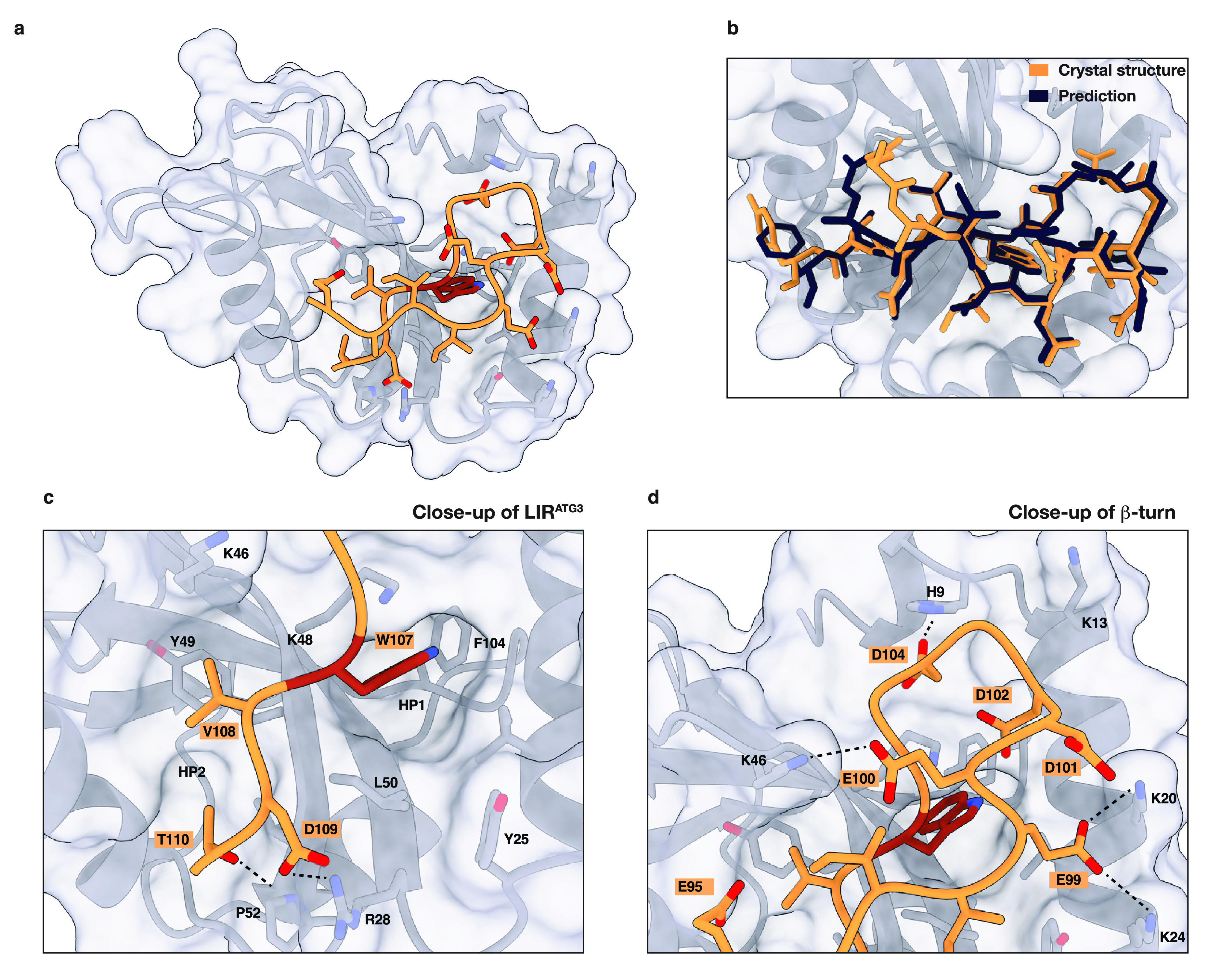
Cocrystal structure of GABARAP and LIR^ATG3^. **a**. Cocrystal structure of GABARAP (gray) and LIR^ATG3^ (orange). Side-chains in contact with LIR^ATG3^ are shown. For clarity, W107 is colored in red. One copy of the GABARAP-LIR^ATG3^ complex was chosen from the asymmetric unit. Other copies in the asymmetric unit show similar structures of the complex. **b**. Zoom-in and overlap of LIR^ATG3^ (orange) with structure of LIR^ATG3^ predicted by ColabFold (dark blue); backbone-atom RMSD is 0.61 Å. **c**. Zoom-in of core LIR^ATG3^ motif binding to hydrophobic pockets HP1 and HP2. H-bonding and ionic interactions are indicated by dashed lines. **d**. Zoom-in of hairpin turn and the interaction of LIR^ATG3^ with basic residues in GABARAP. Residues E95–D104 have been omitted for clarity. H-bonding and ionic interactions are indicated by dashed lines.

To further corroborate our findings, we cocrystallized GABARAP with an LIR^ATG3^ peptide (Y90–H112). We solved the cocrystal structure at a resolution of 2.6 Å and could resolve amino acids E95–T110 of the LIR^ATG3^ peptide (Table S1, Figure 3a). GABARAP adopted its previously described closed conformation and showed no structural rearrangements.^41^ The structure of the LIR peptide and also its interactions with GABARAP were consistent with respect to the ColabFold prediction with a backbone-atom root-mean-square deviation of 0.61 Å (Figure 3b). ATG3 W107 binds to HP1 via hydrophobic interactions in a deep pocket formed by GABARAP P30, K48, and F104. The binding mode is identical to that observed for other LIR motifs (Figure 3c). ATG3 V108 is buried by hydrophobic interactions with GABARAP K46 and Y49 and the upper strand of the LIR β-sheet (E95, I97). These interactions block any solvent contact of V108 and likely strengthen its hydrophobic packing. ATG3 D109 forms a surface-exposed salt bridge with R28. ATG3 T110 binds to HP2 akin to the more conserved Ile, Leu, and Val residues commonly observed in LIR motifs. The shallow pocket is formed by GABARAP Y49, V51, P52, L55, and L63. Thr110 likely mimics the canonical LIR residues, Ile, Leu, and Val, by rotation of its methyl group to form hydrophobic contacts with HP2, enabling its alcohol moiety to participate in H-bonding interactions with Pro52. To form the observed β-sheet conformation, residues N-terminal of W107 form a hairpin turn consisting of glycine and aspartic acid residues, mainly binding via H-bonding and electrostatic interactions to GABARAP. ATG3 D104 binds to H9 via a hydrogen bond. Aspartic and glutamic acid residues E99, E100, D101, and D102 form salt bridges with various basic residues, K20, K24, and K48 (Figure 3d). Following residue E99, a short β-sheet is formed for which backbone atoms could be resolved until E95. Overall, the interaction between the ATG3 LIR motif and GABARAP buries 660 Å^2^.^42^

**Figure 4:**
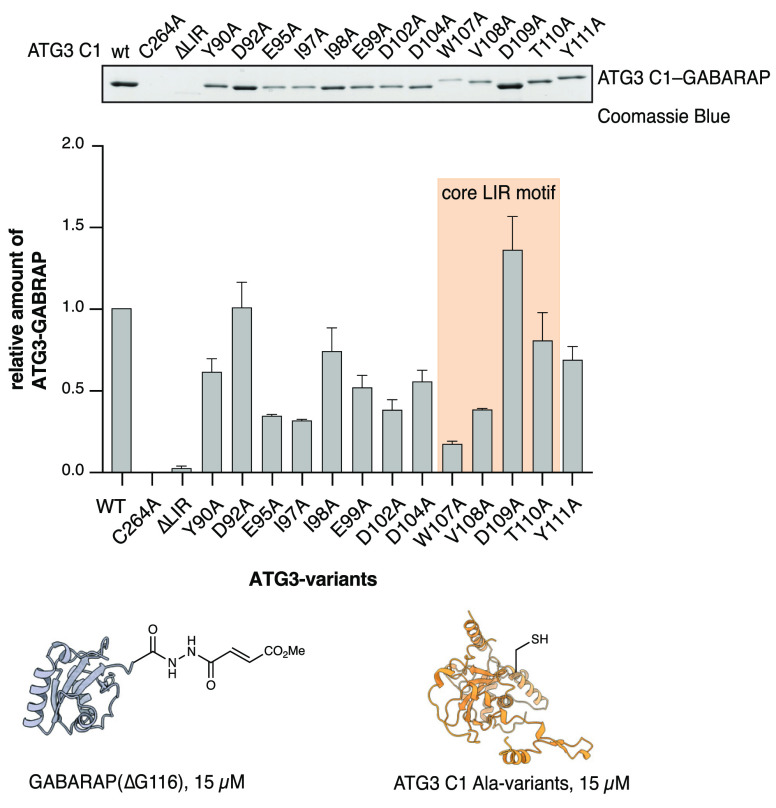
Dissection of interactions between GABARAP and LIR^ATG3^ by alanine scanning. GABARAP probe **2** (15 μM) was reacted with ATG3 C1 alanine variants (15 μM) for 30 min. Coomassie Blue stained SDS-PAGE gel showing modification of ATG3 C1 variants with GABARAP methyl fumarate. The indicated ATG3 residues were mutated to alanine. Cross-linking efficiency was estimated by gel densitometry and normalized to the reaction with ATG3 C1. Data are presented as average values ± s.d. *n* = 2 independent experiments. Full-gel images are available in the Supplementary Information.

Based on the predicted ATG3-GABARAP model and our cocrystal structure, we identified residues in the LIR motif involved in the ATG3-GABARAP interaction. To probe the contribution of LIR^ATG3^ residues to GABARAP binding we performed an Ala-screen using our previously established cross-linking assay as the readout (Figure 4). Cross-linking efficiency of probe **2** with ATG3 or its mutants is dependent on the affinity of the LIR motif. We expressed ATG3 alanine mutants of the identified residues and reacted them with probe **2**. As expected for LIR motifs, mutation of W107 almost completely abrogated the reaction of **2** with ATG3. Similarly, V108A significantly reduced the cross-linking efficiency. This is in agreement with observations for other LIR motifs showing that the first variable position contributes significantly to the binding of LIR motifs.^43^ Intriguingly, mutation of D109 or T110 had almost no effect on reaction efficiency. Additionally, the majority of the other screened residues have at least some impact on the conjugation with GABARAP. E95 and I97 severely diminish cross-linking supporting our hypothesis that these residues are important for β-sheet formation and burying V108. The hairpin turn binds mainly via electrostatic interactions to GABARAP. Increasing the salt concentration disrupts this interaction as shown by reduced fluorescence polarization of LIR^ATG3^ peptide upon binding to GABARAP and cross-linking with ATG3 (Figure S13). These data highlight how binding of LIR^ATG3^ to GABARAP/LC3A is driven by a combination of hydrophobic and ionic interactions.

### LIR Motif Affects LC3 Lipidation and Thioester Transfer *In Cellulo*

LIR motifs are crucial to the function of various proteins involved in autophagy including selective autophagy receptors as well as enzymes involved in LC3 lipidation such as ATG7^44^ or ATG4.^12^ The LIR motif in yeast ATG3 was shown to affect Atg8 lipidation *in vitro* and also affects the cytoplasm-to-vacuole pathway.^25,45^

**Figure 5:**
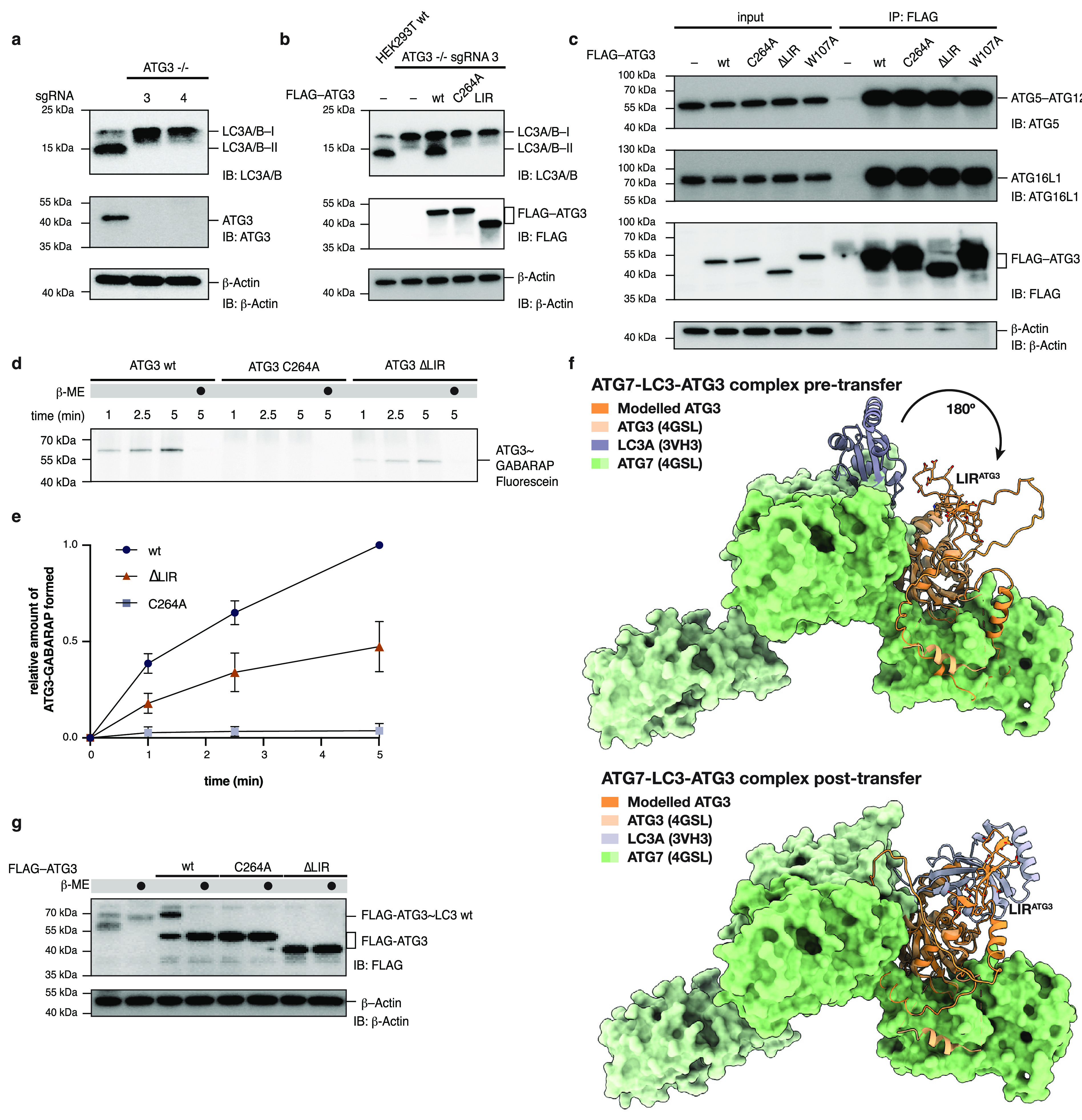
LIR^ATG3^ effects LC3 lipidation and thioester transfer. **a**. Generation of homozygous knockouts of HEK293T cells using CRISPR-Cas9 gene editing. Gene editing efficiency was validated by immunoblotting against ATG3 and LC3A/B. Analysis for two knockout clones is shown. **b**. Rescue with FLAG-ATG3 variants in HEK293T ATG3 – /– cells and autophagy induced by starvation for 1 h in the presence of chloroquine (40 μM). Lipidation was assessed by immunoblotting against LC3A/B. As a positive control wild-type HEK293T cells were starved and analyzed in parallel to ATG3 – /– cells. **c**. Coimmunoprecipitation of FLAG-ATG3 variants (wt, C264A, ΔLIR, and W107A). Interaction of ATG3 with ATG5–ATG12 and ATG16L1 was assessed by immunoblotting. **d**. Pulse-chase assay of GABARAP transfer from ATG7 to ATG3. ATG7 was charged with fluorescein-labeled GABARAP. The reaction was quenched by addition of EDTA, and transfer initiated by addition of ATG3 variants (wt, C264A, ΔLIR). The reaction was monitored for the indicated time-points. Thioester-transfer was assessed by SDS-PAGE and in-gel fluorescence. **e**. Quantification of in-gel fluorescence of **d**. **f**. Structural models showing the potential role of LIR^ATG3^ in thioester transfer from ATG7 to ATG3. ATG3–ATG7 complex was modeled and overlaid with the previously reported structure (PDB: 4GHL). The Atg7-Atg8 model^44^ (PDB: 3VH3) was overlaid with structures. For the post-transfer model, the ATG3–GABARAP model from Figure 1c was used and aligned to ATG3 of 4GHL. **g**. Detection of ATG3∼LC3A/B thioester *in cellulo*. FLAG-ATG3 variants were transiently expressed in HCT116 cells in which endogenous ATG3 was knocked down by CRISPR inhibition (CRISPRi). Autophagy was induced by starvation in the presence of chloroquine (40 μM) for 1 h. Cell lysates were prepared either in the absence or in the presence of β-mercaptoethanol (β-ME). Samples containing reducing agent were boiled prior to SDS-PAGE analysis. The presence of a thioester intermediate was analyzed by anti-FLAG immunoblotting. ATG3∼LC3A/B thioester was only observed for ATG3 wt. Data are presented as average values ± s.d. and normalized to gel density of ATG3 wt 5 min. *n* = 3 independent experiments. Full-gel images for **a**–**e** are available in the Supplementary Information.

To gauge the role of the LIR motif in human ATG3, we generated a homozygous HEK293T ATG3 knockout (KO) cell line using CRISPR-Cas9. The knockout was validated by immunoblotting against ATG3 and LC3A/B showing defective LC3 lipidation (Figure 5a). Upon starvation, rescue with FLAG-ATG3 wild-type led to LC3 lipidation as evidenced by the presence of the LC3–II band. However, expression of ATG3 C264A or ATG3 ΔLIR did not induce LC3 lipidation (Figure 5b, Figure S14). This finding indicates that the LIR motif in human ATG3 is a prerequisite for LC3 lipidation by ATG3 and contrasts with *S. cerevisiae,* in which the LIR motif had no effect on starvation induced Atg8 lipidation.

### How Does LIR^ATG3^ Influence ATG3’s Activity?

We investigated the interaction of ATG3 with components of its enzymatic cascade by coimmunoprecipitation. We transiently expressed FLAG-ATG3 variants in HCT116 ATG3 knock-down cells generated using CRISPRi (Figure S15) and assessed their interaction with E3 complex subunits by anti-FLAG Co-IP.^46^ ATG3 wt, C264A, ΔLIR, and W107A precipitated the ATG5-ATG12 complex and ATG16L1 with similar efficiency (Figure 5c). Therefore, LIR^ATG3^ does not affect lipidation through defective binding to the E3 enzyme complex. Likewise, we performed pull-down of recombinantly expressed ATG3 variants with FLAG-ATG7 immobilized on anti-FLAG resin. No difference in pull-down efficiency was observed (Figure S16). This indicates that LIR^ATG3^ or the lack thereof does not impact binding of ATG3 to ATG7 via its ATG7 interacting region (RIA7).^47^

In order to assess the influence of LIR^ATG3^ on thioester transfer from ATG7 to ATG3 we performed a pulse-chase assay. Fluorescein-labeled GABARAP was charged onto ATG7 and thioester transfer initiated by addition of ATG3 variants. We observed a significant reduction in transfer rate between ATG3 wt and ΔLIR (Figure 5d,e). This observation contrasts with *S. cerevisiae* Atg3 in which the LIR motif had no effect on Atg8 transfer. In order to understand the origin of this rate defect we modeled ATG3 onto the E1 enzyme ATG7 using ColabFold. We correctly predicted RIA7 of ATG3 and its interaction with ATG7.^47^ The predicted structure is in good agreement with a previously reported structure of the ATG7–ATG3 complex.^48^ Overlaying the predicted structure with a reported structure of Atg8 bound to Atg7 places LIR^ATG3^ in proximity (20–30 Å) of the LIR binding region of LC3/Atg8. Based on this proximity we speculate that the LIR motif aids ATG3 in abstracting LC3 from ATG7 by facilitating thioester exchange (Figure 5f). LC3 can undergo a 180° rotation around its C-terminus to adopt the closed conformation observed in our ColabFold prediction (Figure 1c).

To confirm these *in vitro* results *in cellulo*, we expressed FLAG-ATG3 variants in HEK293T cells to analyze the presence of the ATG3∼LC3A/B thioester complex formation. Cell lysis under reducing or nonreducing conditions showed the presence of a thiol-sensitive band for wild-type ATG3. This band corresponds to the ATG3∼LC3 thioester complex, as no thioester was detected for ATG3 C264A (Figure 5g). With ATG3 ΔLIR no higher MW thiol-sensitive band was detected either. This observation suggests that LIR^ATG3^ influences ATG3∼LC3A/B complex beyond a defect in its formation potentially due to complex stability.

## Conclusions

Using a combination of chemical, biochemical, computational, and structural methods we have identified a previously unknown LIR motif in human ATG3. The LIR motif is distinct from a previously reported LIR motif in *S. cerevisiae* Atg3 (LIR^SC^), which occurs in a completely different part of its homologue. Sequence alignment shows that the yeast LIR motif was lost and replaced by the LIR motif described herein. These findings suggest an evolutionary advantage of the human LIR (LIR^HS^) motif over LIR^SC^. The conservation of LIR^HS^ among other species including most fungi suggests that *S. cerevisiae* is a poor model to derive general conclusions on the enzymatic function of ATG3 across different species.

The core LIR motif, WVDT, is noncanonical and embedded in an unusual β-sheet conformation. Amino acids beyond the core LIR motif and the β-sheet conformation are involved in ATG3 binding to LC3/GABARAP. This uncommon β-sheet conformation for LIR motifs has only been reported for a few cases, including the FNIP tumor suppressor,^33^ the *Legionella* effector protein RavZ.^32^ A cursory search of reported LIR structures suggests that ALFY likely contains a β-sheet embedded LIR motif.^49^ We provide conclusive evidence that the bent β-sheet conformation for LIR^ATG3^ is required for efficient binding to LC3/GABARAP and is not an artifact of cocrystallizing LIR peptide and LC3/GABARAP. This finding suggests that bent LIR conformations may be more prevalent than currently appreciated. It is likely that a more widespread identification of this conformation has been precluded to this date by investigating LIR motifs in structural studies that were truncated in their N-terminal region and could not form the β-sheet conformation. This information will be useful in guiding bioinformatics methods to identify previously unidentified LIR motifs and extending the realm of LIR structures.

In addition, we show that LIR^ATG3^ is required for LC3 lipidation *in cellulo* and effects efficient thioester transfer from ATG7 to ATG3. While we observed no requirement of LIR^ATG3^ for interaction with either ATG5–ATG12, ATG16L1, or ATG7, the LIR motif influences the catalytic activity of the complex. ATG3’s flexible region contains not only the LIR motif but also two additional amino acid stretches required for ATG7 interaction, RIA7, and ATG12, RIA12. RIA7 and RIA12 partially overlap and are therefore mutually exclusive in their binding. It is probable that LIR^ATG3^ facilitates or influences binding of either region to their respective binding partner and therefore drives the lipidation reaction forward. It is also plausible that LIR^ATG3^ binds to and blocks the LIR-binding region of LC3/GABARAP from binding to the plethora of proteins bearing LIR motifs during its transfer from ATG7 to ATG3 to its substrate lipid. Interaction of LC3/GABARAP with one of these effector proteins during its transfer would likely negatively impact the efficiency of the lipidation reaction and stall efficient autophagosome expansion.

## Data Availability

Atomic coordinates and structure factors for the reported crystal structure have been deposited at the Protein Data Bank (PDB) with accession code 8AFI.

## Supporting Information Available

Figures, methods, protein sequences, including characterization by HPLC and mass spectrometry, denaturing PAGE, and Western blot analysis (PDF)
Transparent Peer Review report available (PDF)

## Notes

Safety Statement. No unexpected or unusually high safety hazards were encountered.

## References

[ref1] Bento C. F.; Renna M.; Ghislat G.; Puri C.; Ashkenazi A.; Vicinanza M.; Menzies F. M.; Rubinsztein D. C. Mammalian Autophagy: How Does It Work?. Annu. Rev. Biochem. 2016, 85, 685–713. 10.1146/annurev-biochem-060815-014556. 26865532

[ref2] Pankiv S.; Clausen T. H.; Lamark T.; Brech A.; Bruun J. A.; Outzen H.; Overvatn A.; Bjorkoy G.; Johansen T. p62/SQSTM1 binds directly to Atg8/LC3 to facilitate degradation of ubiquitinated protein aggregates by autophagy. J. Biol. Chem. 2007, 282 (33), 24131–24145. 10.1074/jbc.M702824200. 17580304

[ref3] Novak I.; Kirkin V.; McEwan D. G.; Zhang J.; Wild P.; Rozenknop A.; Rogov V.; Lohr F.; Popovic D.; Occhipinti A.; et al. Nix is a selective autophagy receptor for mitochondrial clearance. Embo Rep 2010, 11 (1), 45–51. 10.1038/embor.2009.256. 20010802 PMC2816619

[ref4] Tumbarello D. A.; Manna P. T.; Allen M.; Bycroft M.; Arden S. D.; Kendrick-Jones J.; Buss F. The Autophagy Receptor TAX1BP1 and the Molecular Motor Myosin VI Are Required for Clearance of Salmonella Typhimurium by Autophagy. Plos Pathog 2015, 11 (10), e1005174 10.1371/journal.ppat.1005174. 26451915 PMC4599966

[ref5] Ichimura Y.; Kirisako T.; Takao T.; Satomi Y.; Shimonishi Y.; Ishihara N.; Mizushima N.; Tanida I.; Kominami E.; Ohsumi M.; et al. A ubiquitin-like system mediates protein lipidation. Nature 2000, 408 (6811), 488–492. 10.1038/35044114. 11100732

[ref6] Maruyama T.; Alam J. M.; Fukuda T.; Kageyama S.; Kirisako H.; Ishii Y.; Shimada I.; Ohsumi Y.; Komatsu M.; Kanki T.; et al. Membrane perturbation by lipidated Atg8 underlies autophagosome biogenesis. Nat. Struct Mol. Biol. 2021, 28 (7), 583 10.1038/s41594-021-00614-5. 34239122

[ref7] Nguyen T. N.; Padman B. S.; Usher J.; Oorschot V.; Ramm G.; Lazarou M. Atg8 family LC3/GABARAP proteins are crucial for autophagosome-lysosome fusion but not autophagosome formation during PINK1/Parkin mitophagy and starvation. J. Cell Biol. 2016, 215 (6), 857–874. 10.1083/jcb.201607039. 27864321 PMC5166504

[ref8] Manil-Segalen M.; Lefebvre C.; Jenzer C.; Trichet M.; Boulogne C.; Satiat-Jeunemaitre B.; Legouis R.; The C. elegans LC3 Acts Downstream of GABARAP to Degrade Autophagosomes by Interacting with the HOPS Subunit VPS39 (vol 28, pg 43, 2014). Dev Cell 2014, 30 (1), 110–110. 10.1016/j.devcel.2014.06.030. 24374177

[ref9] Weidberg H.; Shvets E.; Shpilka T.; Shimron F.; Shinder V.; Elazar Z. LC3 and GATE-16/GABARAP subfamilies are both essential yet act differently in autophagosome biogenesis. EMBO J. 2010, 29 (11), 1792–1802. 10.1038/emboj.2010.74. 20418806 PMC2885923

[ref10] Noda N. N.; Kumeta H.; Nakatogawa H.; Satoo K.; Adachi W.; Ishii J.; Fujioka Y.; Ohsumi Y.; Inagaki F. Structural basis of target recognition by Atg8/LC3 during selective autophagy. Genes Cells 2008, 13 (12), 1211–1218. 10.1111/j.1365-2443.2008.01238.x. 19021777

[ref11] Birgisdottir A. B.; Lamark T.; Johansen T. The LIR motif - crucial for selective autophagy. J. Cell Sci. 2013, 126 (15), 3237–3247. 10.1242/jcs.126128. 23908376

[ref12] Skytte Rasmussen M.; Mouilleron S.; Kumar Shrestha B.; Wirth M.; Lee R.; Bowitz Larsen K.; Abudu Princely Y.; O’Reilly N.; Sjottem E.; Tooze S. A.; et al. ATG4B contains a C-terminal LIR motif important for binding and efficient cleavage of mammalian orthologs of yeast Atg8. Autophagy 2017, 13 (5), 834–853. 10.1080/15548627.2017.1287651. 28287329 PMC5446077

[ref13] Fan S. J.; Yue L. Y.; Wan W.; Zhang Y. Y.; Zhang B. D.; Otomo C.; Li Q. F.; Lin T. T.; Hu J. C.; Xu P.; et al. Inhibition of Autophagy by a Small Molecule through Covalent Modification of the LC3 Protein. Angew. Chem. Int. Edit 2021, 60 (50), 26105–26114. 10.1002/anie.202109464. PMC8845813 34590387

[ref14] Yang A. M.; Pantoom S.; Wu Y. W. Elucidation of the anti-autophagy mechanism of the Legionella effector RavZ using semisynthetic LC3 proteins. Elife 2017, 6, e23905 10.7554/eLife.23905. 28395732 PMC5388539

[ref15] Li Y. T.; Yi C.; Chen C. C.; Lan H.; Pan M.; Zhang S. J.; Huang Y. C.; Guan C. J.; Li Y. M.; Yu L.; et al. A semisynthetic Atg3 reveals that acetylation promotes Atg3 membrane binding and Atg8 lipidation. Nat. Commun. 2017, 8, 14846 10.1038/ncomms14846. 28327644 PMC5473643

[ref16] Norman B. H.; Shih C.; Toth J. E.; Ray J. E.; Dodge J. A.; Johnson D. W.; Rutherford P. G.; Schultz R. M.; Worzalla J. F.; Vlahos C. J. Studies on the mechanism of phosphatidylinositol 3-kinase inhibition by wortmannin and related analogs. J. Med. Chem. 1996, 39 (5), 1106–1111. 10.1021/jm950619p. 8676346

[ref17] Blommaart E. F. C.; Krause U.; Schellens J. P. M.; VreelingSindelarova H.; Meijer A. J. The phosphatidylinositol 3-kinase inhibitors wortmannin and LY294002 inhibit autophagy in isolated rat hepatocytes. Eur. J. Biochem. 1997, 243 (1–2), 240–246. 10.1111/j.1432-1033.1997.0240a.x. 9030745

[ref18] Sui X.; Wang Y.; Du Y. X.; Liang L. J.; Zheng Q. Y.; Li Y. M.; Liu L. Development and application of ubiquitin-based chemical probes. Chem. Sci. 2020, 11 (47), 12633–12646. 10.1039/D0SC03295F. 34123237 PMC8163311

[ref19] Henneberg L. T.; Schulman B. A. Decoding the messaging of the ubiquitin system using chemical and protein probes. Cell Chem. Biol. 2021, 28 (7), 889–902. 10.1016/j.chembiol.2021.03.009. 33831368 PMC7611516

[ref20] Hemelaar J.; Lelyveld V. S.; Kessler B. M.; Ploegh H. L. A single protease, Apg4B, is specific for the autophagy-related ubiquitin-like proteins GATE-16, MAP1-LC3, GABARAP, and Apg8L. J. Biol. Chem. 2003, 278 (51), 51841–51850. 10.1074/jbc.M308762200. 14530254

[ref21] Tolmachova K. A.; Farnung J.; Liang J. R.; Corn J. E.; Bode J. W. Facile Preparation of UFMylation Activity-Based Probes by Chemoselective Installation of Electrophiles at the C-Terminus of Recombinant UFM1. ACS Central Science 2022, 8 (6), 756–762. 10.1021/acscentsci.2c00203. 35756382 PMC9228560

[ref22] Farnung J.; Tolmachova K. A.; Bode J. W. Installation of electrophiles onto the C-terminus of recombinant ubiquitin and ubiquitin-like proteins. Chem. Sci. 2022, 14 (1), 121–129. 10.1039/D2SC04279G. 36605735 PMC9769091

[ref23] Huang D. T.; Paydar A.; Zhuang M.; Waddell M. B.; Holton J. M.; Schulman B. A. Structural basis for recruitment of Ubc12 by an E2 binding domain in NEDD8’s E1. Mol. Cell 2005, 17 (3), 341–350. 10.1016/j.molcel.2004.12.020. 15694336

[ref24] Miura T.; Klaus W.; Gsell B.; Miyamoto C.; Senn H. Characterization of the binding interface between ubiquitin and class I human ubiquitin-conjugating enzyme 2B by multidimensional heteronuclear NMR spectroscopy in solution. J. Mol. Biol. 1999, 290 (1), 213–228. 10.1006/jmbi.1999.2859. 10388568

[ref25] Yamaguchi M.; Noda N. N.; Nakatogawa H.; Kumeta H.; Ohsumi Y.; Inagaki F. Autophagy-related Protein 8 (Atg8) Family Interacting Motif in Atg3Mediates the Atg3-Atg8 Interaction and Is Crucial for the Cytoplasm-to-Vacuole Targeting Pathway. J. Biol. Chem. 2010, 285 (38), 29599–29607. 10.1074/jbc.M110.113670. 20615880 PMC2937991

[ref26] Jumper J.; Evans R.; Pritzel A.; Green T.; Figurnov M.; Ronneberger O.; Tunyasuvunakool K.; Bates R.; Zidek A.; Potapenko A.; et al. Highly accurate protein structure prediction with AlphaFold. Nature 2021, 596 (7873), 583–589. 10.1038/s41586-021-03819-2. 34265844 PMC8371605

[ref27] Evans R.; O’Neill M.; Pritzel A.; Antropova N.; Senior A.; Green T.; Žídek A.; Bates R.; Blackwell S.; Yim J. Protein complex prediction with AlphaFold-Multimer. bioRxiv 2022, 1 10.1101/2021.10.04.463034.

[ref28] Mirdita M.; Schütze K.; Moriwaki Y.; Heo L.; Ovchinnikov S.; Steinegger M. ColabFold: making protein folding accessible to all. Nat. Methods 2022, 19 (6), 679–682. 10.1038/s41592-022-01488-1. 35637307 PMC9184281

[ref29] Pruneda J. N.; Stoll K. E.; Bolton L. J.; Brzovic P. S.; Klevit R. E. Ubiquitin in motion: structural studies of the ubiquitin-conjugating enzyme approximately ubiquitin conjugate. Biochemistry-Us 2011, 50 (10), 1624–1633. 10.1021/bi101913m. PMC3056393 21226485

[ref30] Ibrahim T.; Khandare V.; Mirkin F. G.; Tumtas Y.; Bubeck D.; Bozkurt T. O. AlphaFold2-multimer guided high-accuracy prediction of typical and atypical ATG8-binding motifs. PLoS Biol. 2023, 21 (2), e3001962 10.1371/journal.pbio.3001962. 36753519 PMC9907853

[ref31] Zhang S.; Yazaki E.; Sakamoto H.; Yamamoto H.; Mizushima N. Evolutionary diversification of the autophagy-related ubiquitin-like conjugation systems. Autophagy 2022, 18 (12), 2969–2984. 10.1080/15548627.2022.2059168. 35427200 PMC9673942

[ref32] Kwon D. H.; Kim L.; Kim B. W.; Kim J. H.; Roh K. H.; Choi E. J.; Song H. K. A novel conformation of the LC3-interacting region motif revealed by the structure of a complex between LC3B and RavZ. Biochem Bioph Res. Co 2017, 490 (3), 1093–1099. 10.1016/j.bbrc.2017.06.173. 28668392

[ref33] Goodwin J. M.; Walkup W. G.; Hooper K.; Li T. Y. N.; Kishi-Itakura C.; Ng A.; Lehmberg T.; Jha A.; Kommineni S.; Fletcher K. GABARAP sequesters the FLCN-FNIP tumor suppressor complex to couple autophagy with lysosomal biogenesis. Sci. Adv. 2021, 7 (40), 1 10.1126/sciadv.abj2485. PMC10938568 34597140

[ref34] Park S. W.; Jeon P.; Yamasaki A.; Lee H. E.; Choi H.; Mun J. Y.; Jun Y. W.; Park J. H.; Lee S. H.; Lee S. K.; et al. Development of new tools to study membrane-anchored mammalian Atg8 proteins. Autophagy 2022, 1–20. 10.1080/15548627.2022.2132040. PMC10240976 36250672

[ref35] Huber J.; Obata M.; Gruber J.; Akutsu M.; Lohr F.; Rogova N.; Guntert P.; Dikic I.; Kirkin V.; Komatsu M.; et al. An atypical LIR motif within UBA5 (ubiquitin like modifier activating enzyme 5) interacts with GABARAP proteins and mediates membrane localization of UBA5. Autophagy 2020, 16 (2), 256–270. 10.1080/15548627.2019.1606637. 30990354 PMC6984602

[ref36] von Muhlinen N.; Akutsu M.; Ravenhill B. J.; Foeglein A.; Bloor S.; Rutherford T. J.; Freund S. M. V.; Komander D.; Randow F. LC3C, Bound Selectively by a Noncanonical LIR Motif in NDP52, Is Required for Antibacterial Autophagy. Mol. Cell 2012, 48 (3), 329–342. 10.1016/j.molcel.2012.08.024. 23022382 PMC3510444

[ref37] Zorzi A.; Deyle K.; Heinis C. Cyclic peptide therapeutics: past, present and future. Curr. Opin Chem. Biol. 2017, 38, 24–29. 10.1016/j.cbpa.2017.02.006. 28249193

[ref38] Brown H.; Chung M.; Üffing A.; Batistatou N.; Tsang T.; Doskocil S.; Mao W.; Willbold D.; Bast R. C. Jr.; Lu Z.; et al. Structure-Based Design of Stapled Peptides That Bind GABARAP and Inhibit Autophagy. J. Am. Chem. Soc. 2022, 144 (32), 14687–14697. 10.1021/jacs.2c04699. 35917476 PMC9425296

[ref39] Goto Y.; Ohta A.; Sako Y.; Yamagishi Y.; Murakami H.; Suga H. Reprogramming the translation initiation for the synthesis of physiologically stable cyclic peptides. ACS Chem. Biol. 2008, 3 (2), 120–129. 10.1021/cb700233t. 18215017

[ref40] Bechtler C.; Lamers C. Macrocyclization strategies for cyclic peptides and peptidomimetics. Rsc Med. Chem. 2021, 12 (8), 1325–1351. 10.1039/D1MD00083G. 34447937 PMC8372203

[ref41] Coyle J. E.; Qamar S.; Rajashankar K. R.; Nikolov D. B. Structure of GABARAP in two conformations: implications for GABA(A) receptor localization and tubulin binding. Neuron 2002, 33 (1), 63–74. 10.1016/S0896-6273(01)00558-X. 11779480

[ref42] Pettersen E. F.; Goddard T. D.; Huang C. R. C.; Meng E. E. C.; Couch G. S.; Croll T. I.; Morris J. H.; Ferrin T. E. UCSF ChimeraX: Structure visualization for researchers, educators, and developers. Protein Sci. 2021, 30 (1), 70–82. 10.1002/pro.3943. 32881101 PMC7737788

[ref43] Wirth M.; Zhang W.; Razi M.; Nyoni L.; Joshi D.; O’Reilly N.; Johansen T.; Tooze S. A.; Mouilleron S. Molecular determinants regulating selective binding of autophagy adapters and receptors to ATG8 proteins. Nat. Commun. 2019, 10 (1), 2055 10.1038/s41467-019-10059-6. 31053714 PMC6499816

[ref44] Noda N. N.; Satoo K.; Fujioka Y.; Kumeta H.; Ogura K.; Nakatogawa H.; Ohsumi Y.; Inagaki F. Structural basis of Atg8 activation by a homodimeric E1, Atg7. Mol. Cell 2011, 44 (3), 462–475. 10.1016/j.molcel.2011.08.035. 22055191

[ref45] Sakoh-Nakatogawa M.; Kirisako H.; Nakatogawa H.; Ohsumi Y. Localization of Atg3 to autophagy-related membranes and its enhancement by the Atg8-family interacting motif to promote expansion of the membranes. Febs Lett. 2015, 589 (6), 744–749. 10.1016/j.febslet.2015.02.003. 25680528

[ref46] Kuma A.; Mizushima N.; Ishihara N.; Ohsumi Y. Formation of the approximately 350-kDa Apg12-Apg5.Apg16 multimeric complex, mediated by Apg16 oligomerization, is essential for autophagy in yeast. J. Biol. Chem. 2002, 277 (21), 18619–18625. 10.1074/jbc.M111889200. 11897782

[ref47] Ohashi K.; Otomo T. Identification and characterization of the linear region of ATG3 that interacts with ATG7 in higher eukaryotes. Biochem Bioph Res. Co 2015, 463 (3), 447–452. 10.1016/j.bbrc.2015.05.107. PMC4507496 26043688

[ref48] Yamaguchi M.; Matoba K.; Sawada R.; Fujioka Y.; Nakatogawa H.; Yamamoto H.; Kobashigawa Y.; Hoshida H.; Akada R.; Ohsumi Y.; et al. Noncanonical recognition and UBL loading of distinct E2s by autophagy-essential Atg7. Nat. Struct Mol. Biol. 2012, 19 (12), 1250–1256. 10.1038/nsmb.2451. 23142983

[ref49] Lystad A. H.; Ichimura Y.; Takagi K.; Yang Y. J.; Pankiv S.; Kanegae Y.; Kageyama S.; Suzuki M.; Saito I.; Mizushima T.; et al. Structural determinants in GABARAP required for the selective binding and recruitment of ALFY to LC3B-positive structures. Embo Rep 2014, 15 (5), 557–565. 10.1002/embr.201338003. 24668264 PMC4210083

