# Supplementary material for: Semisynthetic LC3 Probes for Autophagy Pathways Reveal a Noncanonical LC3 Interacting Region Motif Crucial for the Enzymatic Activity of Human ATG3
Source: ACS Cent Sci. 2023 Apr 27;9(5):1025–34. doi: 10.1021/acscentsci.3c00009 (PMC10214526; doi:10.1021/acscentsci.3c00009)
Supplement: Supplementary file 1 — oc3c00009_si_001.pdf [file oc3c00009_si_001.pdf]

Supplementary Information

Semi-synthetic LC3 probes for autophagy pathways reveal a non-canonical LIR motif crucial for the enzymatic activity of human ATG3

Jakob Farnung,[1] Matthias Muhar,[2] Jin Rui Liang,[2] Kateryna A. Tolmachova,[1]
Roger M. Benoit,[3] Jacob E. Corn,[2] Jeffrey W. Bode[1]*

[1]Laboratory for Organic Chemistry, Department of Chemistry and Applied Biosciences ETH Zürich, CH-8093 Zürich, Switzerland

[2]Institute of Molecular Health Sciences, Department of Biology
ETH Zürich, CH-8093 Zürich, Switzerland

[3]Laboratory of Nanoscale Biology, Division of Biology and Chemistry, Paul Scherrer Institute, 5232 Villigen PSI, Switzerland

* E-mail: bode@org.chem.ethz.ch

Contents

1. Supplementary figures 2
2. General information 13
3. General methods 19
4. Characterization of peptides and proteins 27
5. Amino acid sequences of recombinantly expressed proteins 42
6. Primer sequences 43
7. Uncropped gels and blots 45
8. References 49

1. Supplementary figures

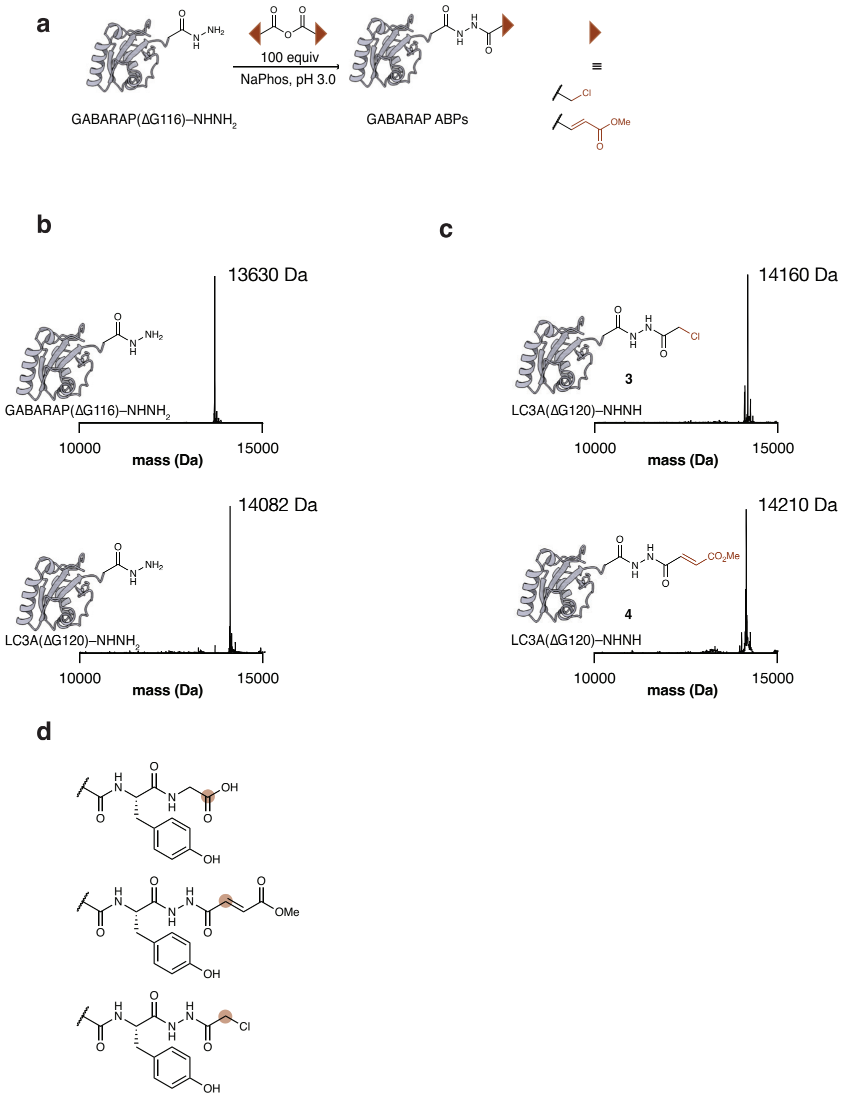

Figure S1. GABARAP and LC3A probe preparation by acyl hydrazide modification. a, Reaction scheme showing modification of GABARAP(ΔG116)–$NHNH_2$ with symmetrical anhydrides at acidic pH. α-chloroacetyl and methyl fumarate probes were prepared. **b**, Deconvoluted mass-spectrum (ESI) of GABARAP(ΔG116)–$NHNH_2$. Expected mass 13630 Da. Deconvoluted mass spectrum (ESI) of LC3A(ΔG120)–$NHNH_2$. Expected mass 14082 Da. **c**, Deconvoluted mass spectrum (ESI) of LC3A(ΔG120)–NHNH α-chloroacetyl **3**. Expected mass 14158 Da. Deconvoluted mass spectrum (ESI) of LC3A(ΔG120)–NHNH methyl fumarate **4**. Expected mass 14210 Da. **d**, Alignment of native C-terminus of GABARAP and C-termini obtained by hydrazide modification. Site of modification is highlighted by brown circle.

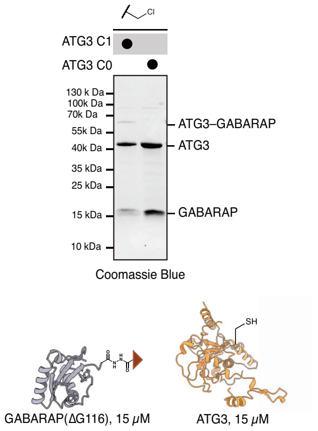

Figure S2. Reaction of GABARAP(ΔG116)–NHNH α-chloroacetyl probe 1 with ATG3 C1. GABARAP probe **1** (15 µM) was incubated with ATG3 C1 or C0 (15 µM) for 1 h and analyzed by Coomassie-Blue staining and SDS-PAGE analysis. Cross-linking was only observed for ATG3 C1.

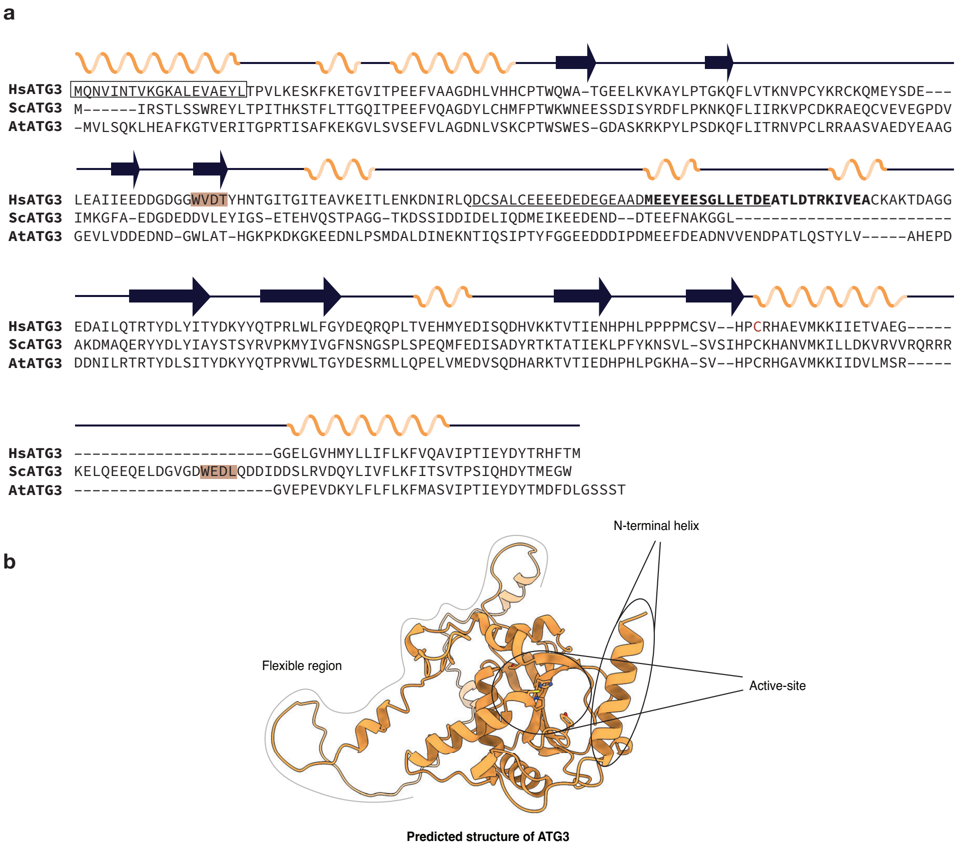

Figure S3. Sequence and structural elements of ATG3. a, Sequence alignment of Homo Sapiens, Saccharomyces cerevisiae ATG3 and Arabidopsis thaliana ATG3. Sequences are aligned and structural elements of HsATG3 are shown. LIR^{ATG3} is highlighted by brown box. N-terminal amphipathic helix is highlighted by box. RIA12 is highlighted by underlined text. RIA7 is highlighted by bold text. AIM^{ATG3} in ScATG3 is highlighted by brown box. **b**, Predicted structure of HsATG3 using Colabfold. Structural elements are indicated.

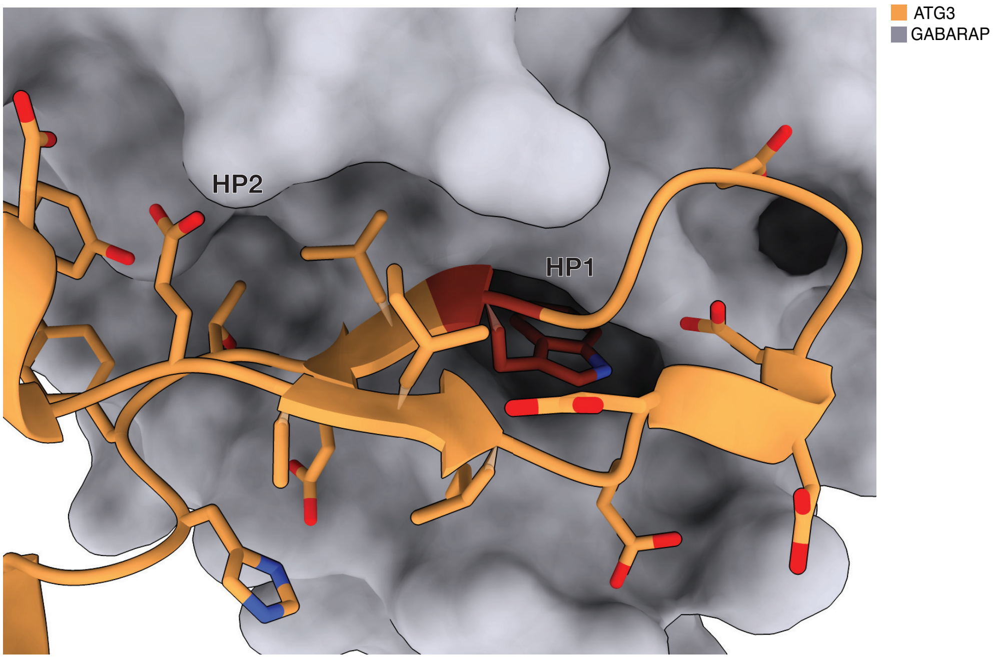

Figure S4. Zoom-in of predicted LIR motif in ATG3. Zoom-in of predicted LIR motif in ATG3. Full-view shown in Figure 1c. W107 and T110 binding to hydrophobic pockets HP1 and HP2, respectively, are indicated. W107 is highlighted in red.

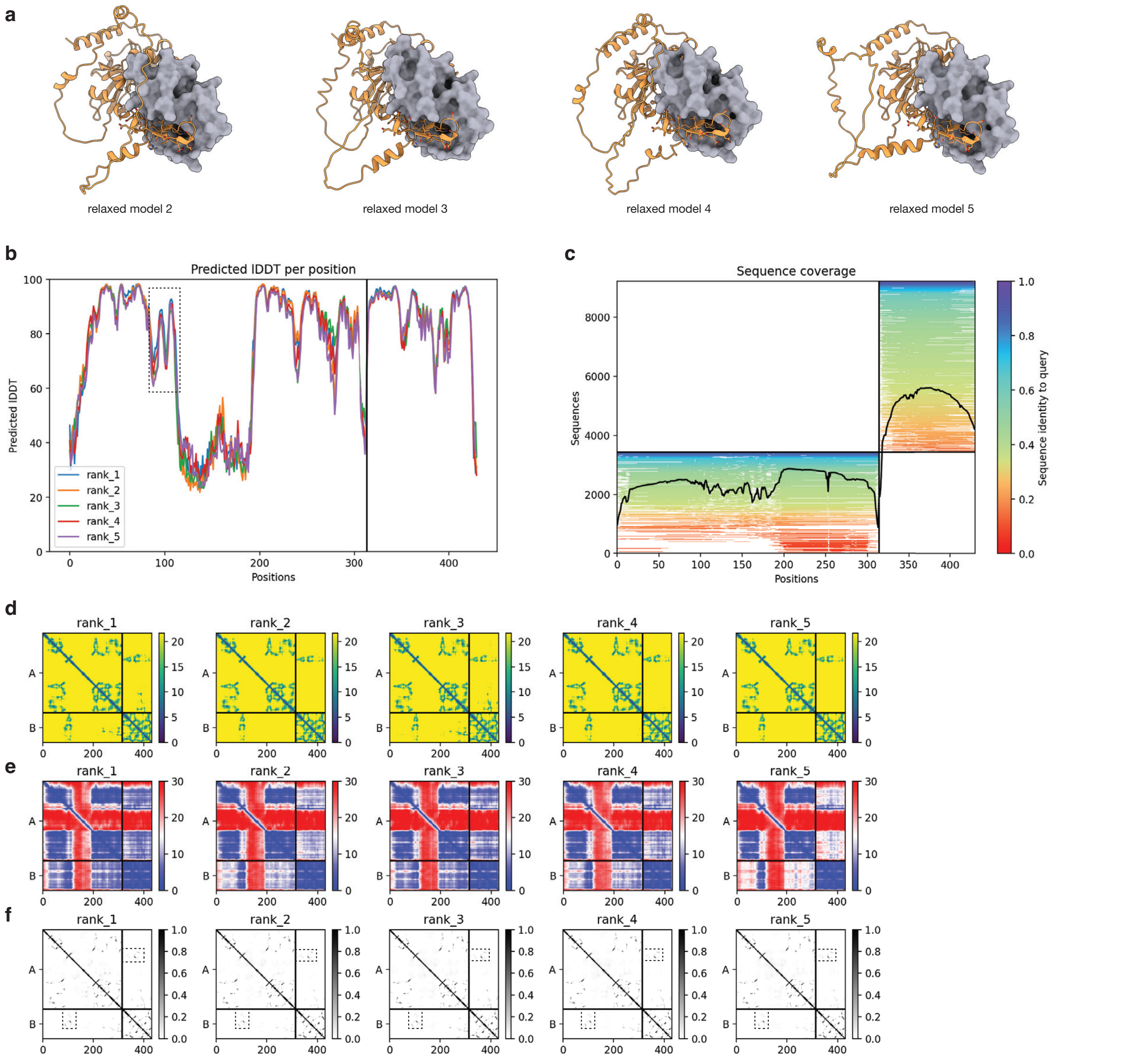

Figure S5. ColabFold prediction of GABARAP–ATG3 complex. a Additional relaxed models predicted by ColabFold for GABARAP–ATG3 complex. Relaxed model 1 shown in Figure 1c. **b** Predicted IDDT values for relaxed models. LIR motif marked with dashed-box. **c** Multi-sequence alignment coverage for relaxed models. **d** Predicted distograms for all relaxed models. **e** Predicted errors of all relaxed models. **f** Predicted contacts of all relaxed models. LIR motif shown in dashed box.

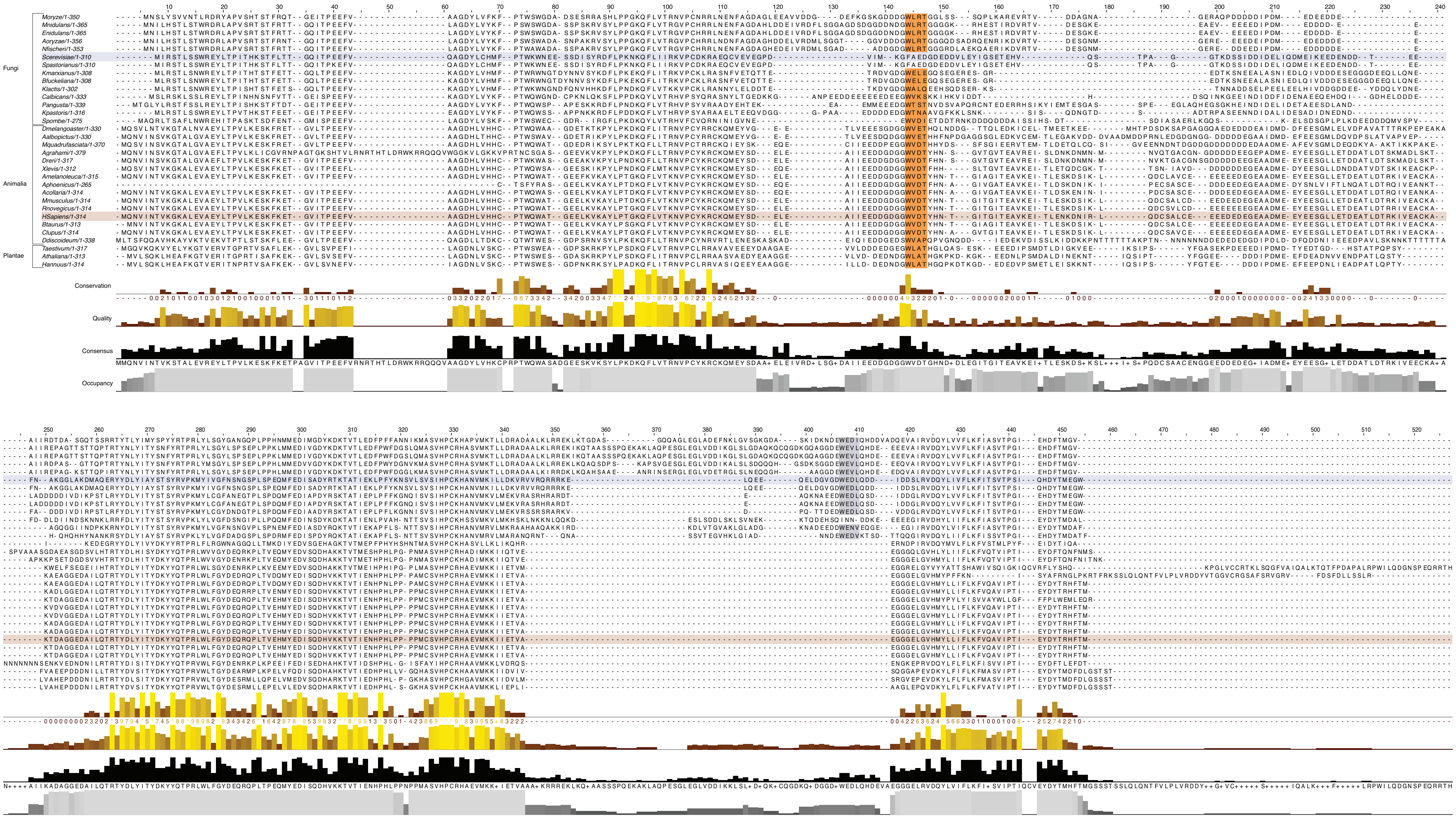

Figure S6. Full sequence alignment of ATG3 variants. Sequence alignment of ATG3 variants from different kingdoms. LIR motif identified in this study highlighted by orange box. LIR motif previously identified in *S. cerevisiae* highlighted by grey box. Sequences for *H. sapiens* and *S. cerevisiae* are highlighted. Both LIR motifs are >150 amino acids apart in sequence space. LIR^{SC} is absent from higher organisms.

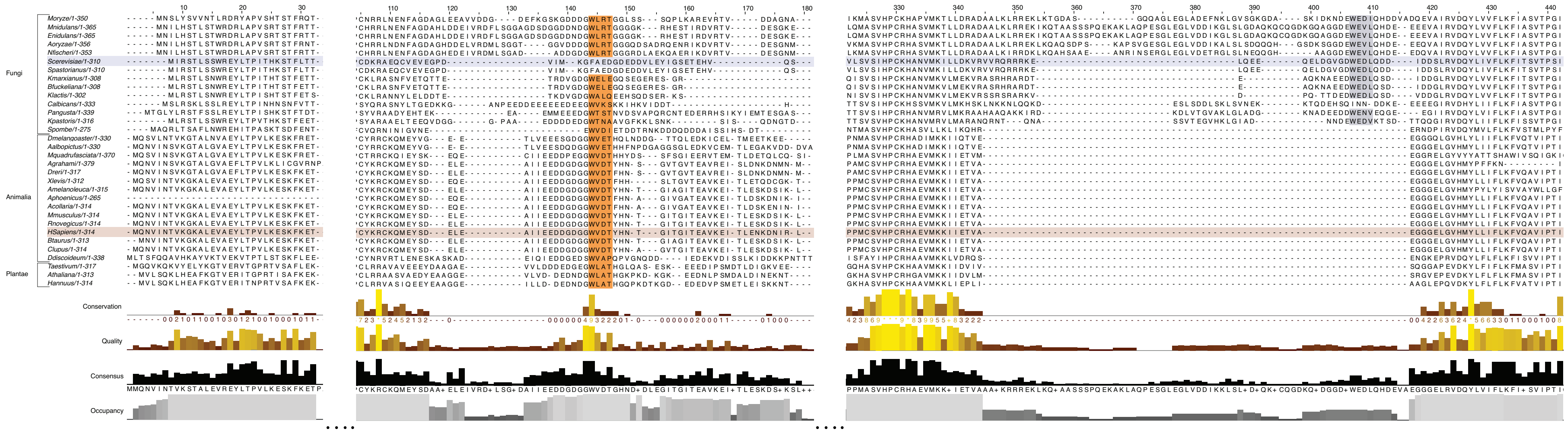

Figure S7. Sequence alignment of ATG3 variants highlighting LIR motifs. Sequence alignments of Supplementary Fig. 6 cropped to highlight both LIR motifs. LIR motif identified in this study highlighted by orange box. LIR motif previously identified in *S. cerevisiae* highlighted by grey box. Sequences for *H. sapiens* and *S. cerevisiae* are highlighted. Both LIR motifs are >150 amino acids apart in sequence space. LIR^{SC} is absent from higher organisms.

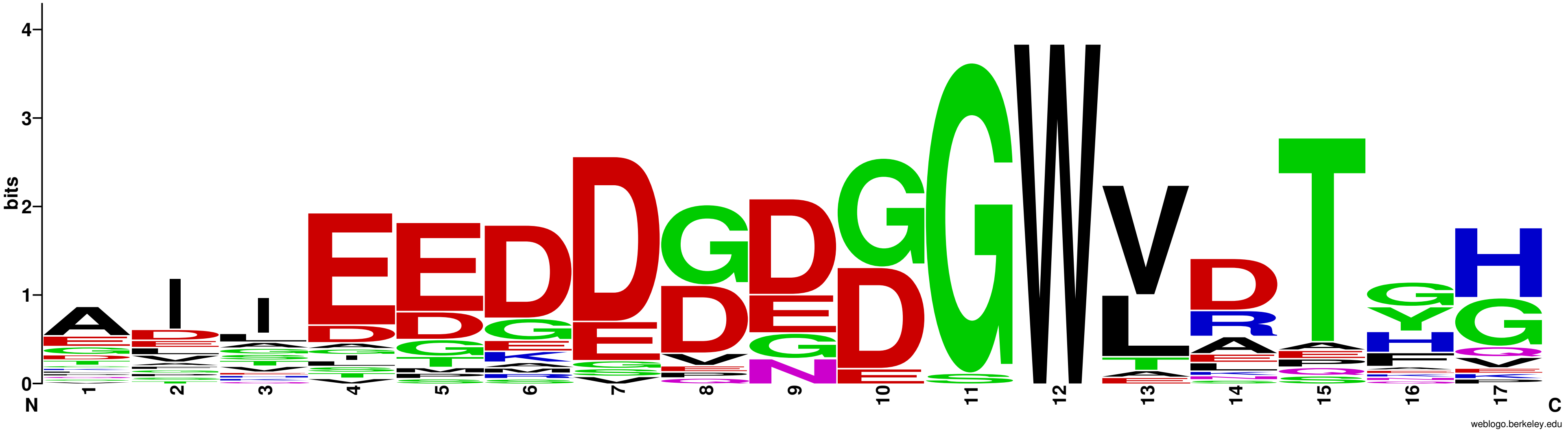

Figure S8. Consensus ATG3 LIR motif derived from sequence alignment. Consensus motif of LIR motifs analogous to human LIR motif derived from organisms used for sequence alignment in Supplementary Figure 6. Consensus motif was generated using Weblogo (http://weblogo.berkeley.edu).

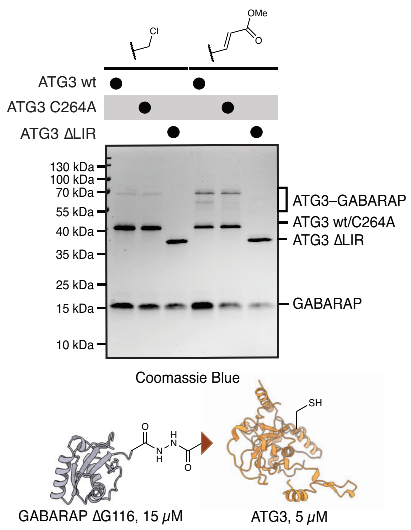

Figure S9. Modification of ATG3 wild-type is dependent on LIR^{ATG3}. Coomassie Blue stained SDS-PAGE gel showing the reaction of GABARAP probes **1** and **2** (15 μM) with ATG3 wt, C264A and ΔLIR (5 μM). Source data and full-gel images are available in the Source Data File.

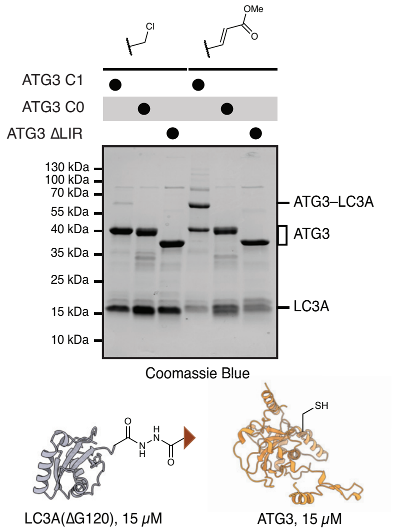

Figure S10. Modification of ATG3 with LC3A is dependent on LIR^{ATG3}. Coomassie Blue stained SDS-PAGE gel showing the reaction of LC3A probes **3** and **4** (15 μM) with ATG3 C1, C0 and ΔLIR (15 μM). Source data and full-gel images are available in the Source Data File.

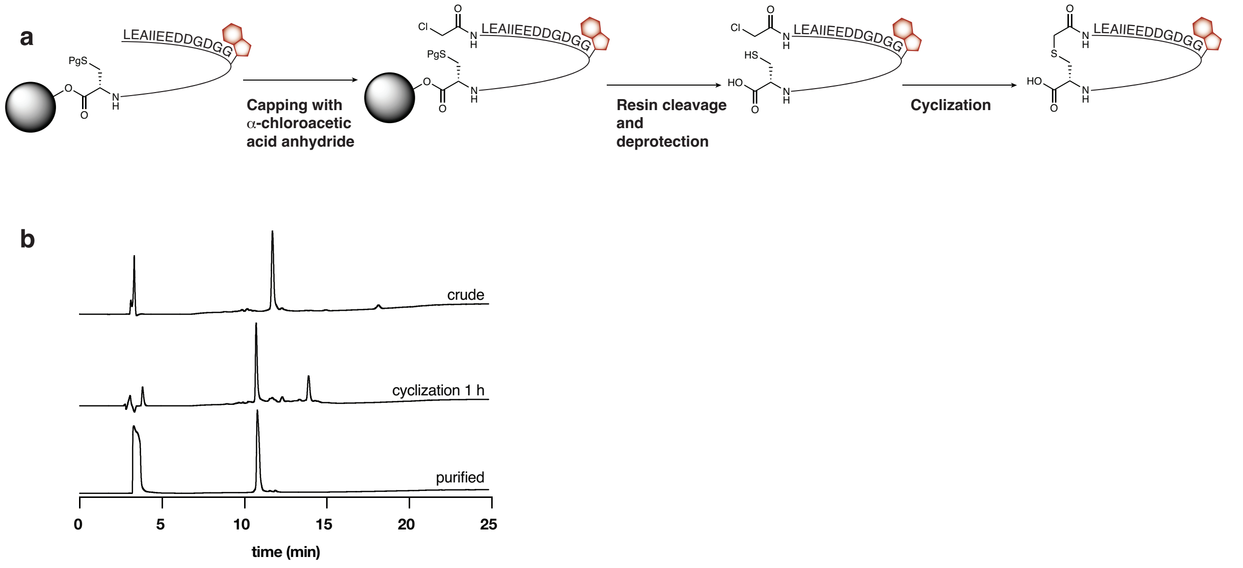

Figure S11. Preparation of cyclized LIR^{ATG3} peptide 6. a, Synthetic scheme describing the preparation of **6**. A cysteine residue is introduced at the C-terminus of the LIR motif. Following standard Fmoc-SPPS the LIR peptide is capped at its N-terminus with α-chloroacetic acid anhydride. Following resin cleavage, the peptide is cyclized by cysteine alkylation. **b**, Analytical RP-HPLC chromatograms showing the cyclization of the LIR peptide to provide **6**. Upper chromatogram shows the linear peptide prior to cyclization. Middle chromatogram shows the cyclization reaction after 1 h. Lower chromatogram shows purified peptide **6**.

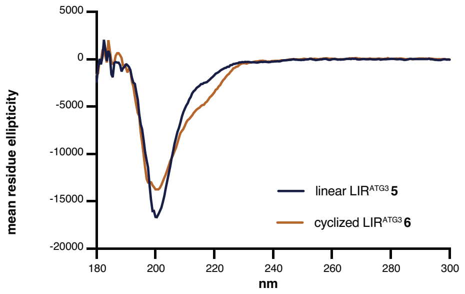

Figure S12. CD spectra of linear and cyclic LIR^{ATG3} peptides 5 and 6. CD spectra of LIR^{ATG3} peptides **5** and **6** were recorded in 20 mM KPhos buffer (pH 7.2) at a concentration of 700 µM. Cyclized LIR^{ATG3} peptide **6** shows a marked shift to a more organized structure as indicated by a decrease of the band intensity at 200 nm and an increase in band intensity at 215 nm.

Parameters	8AFI
**Data collection**	
Wavelength (Å)	1.0
Space group	P1
Cell dimensions	
a, b, c (Å)	62.80, 63.80, 73.90
α, β, γ (°)	64.50, 86.50, 70.30
Resolution (Å)	49.13 – 2.66
R_{merge}	0.068 (1.13)
Completeness (%)	94.7
$I/\sigma(I)$	14.19 (1.02)
$CC_{1/2}$ (%)	99.9 (48.1)
Redundancy	3.5 (3.4)
**Refinement**	
Resolution (Å)	47.14-2.66 (2.75-2.66)
Rwork/Rfree	0.215/0.262
No. reflections	52724
RMSD	
bond lengths (Å)	0.011
bond angles (°)	1.22
No. atoms	
Protein	8485
Ligand	50
water	22
B-factors	
Protein	90.67
Ligand	80.10
water	56.28
Ramachandran plot	
Favored	96.87
Allowed	2.93
Dis-allowed	0.20

Table S1. Data collection and refinement statistics of co-crystal structure of GABARAP with LIR^{ATG3} peptide. Values in parentheses indicated values for high resolution shell.

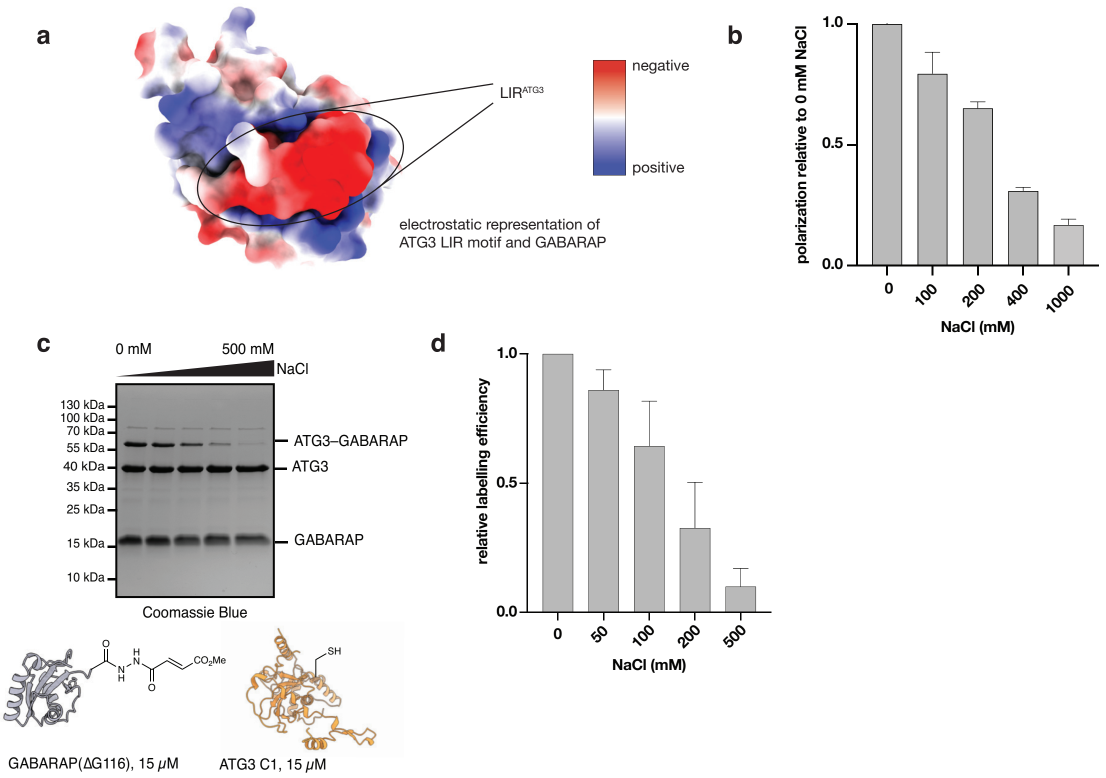

Figure S13. Interaction of ATG3 and GABARAP is salt-dependent. **a**, ColabFold prediction of ATG3-GABARAP complex (in Fig. 1c) shown with its Coulombic potential. Only LIR motif of ATG3 shown for clarity. LIR^{ATG3} is mostly negatively charged and binds to a strongly positively charged surface on GABARAP. **b**, Binding of cyclized LIR^{ATG3} peptide to GABARAP (0.2 µM) in the presence of varying amounts of NaCl. Binding was performed in triplicates and normalized to polarization for 0 mM NaCl. **c**, Modification of ATG3 C1 with **2** in the presence of increasing concentrations of NaCl (0-500 mM). Coomassie Blue stained SDS-PAGE gel was analyzed by gel densitometry. **d**, Quantification of data shown in **b**. Data are presented as average values ± s.d. and normalized to gel density of ATG3 with 0 mM NaCl. n=3 independent experiments. Source data and full-gel images are available in the Source Data File.

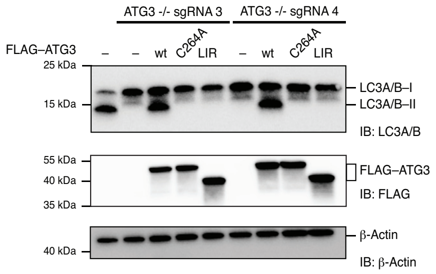

Figure S14. Reconstitution of LC3 lipidation with ATG3 variants in HEK293T ATG3 –/–. FLAG-ATG3 variants were re-expressed in HEK293T ATG3 $^{-/-}$ cells and autophagy induced by starvation for 1 h in the presence of chloroquine (40 μM). Lipidation was assessed by immunoblotting against LC3A/B. Lipidation was observed upon re-expression of ATG3 wt but not ATG3 C264A or ΔLIR. As a positive control wild-type HEK293T cells were starved and analyzed in parallel to ATG3 –/– cells. Data for ATG3 –/– knockout with sgRNA 3 shown in Figure 6b.

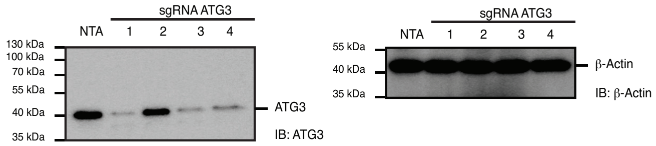

Figure S15. Western blot analysis of HCT116 ATG3 knock-down cells. Western blot analysis anti-ATG3 and anti-β-actin. sgRNAs 1,3 and 4 cause robust knockdown of ATG3 compared to non-targeting sgRNA.

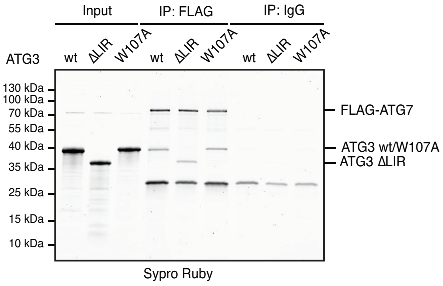

Figure S16. LIR^{ATG3} does not impact the interaction between ATG7 and ATG3. 3xFLAG-ATG7 was expressed in HCT116 cells with endogenous ATG3 knocked-down by CRISPRi and immobilized on Anti-FLAG beads following cell-lysis. Recombinant ATG3 variants (wt, ΔLIR, W107A) were incubated with immobilized ATG7. Bound proteins were eluted by boiling in non-reducing Laemmli buffer and analyzed by Sypro-Ruby staining of SDS-PAGE gel. Antibody fragments are visible after resin elution at 28 kDa. Source data and full-gel are available in the Source Data File.

2. General information

2.1. Reagents and solvents

Chemical reagents were purchased from Sigma Aldrich (Buchs, Switzerland), Acros Organics (Geel, Belgium), TCI Europe (Zwijndrecht, Belgium) and used without further purification. CH_2Cl_2, DMF, EtOH, THF and MeOH were purchased from Fisher Scientific (Geel, Belgium) and Sigma Aldrich (Buchs, Switzerland) and used without further purification (reagent or HPLC grade). Milli-Q water was obtained from a Millipore purification system. Q5 High-Fidelity DNA Polymerase, PCR reagents, Gibson Assembly Master Mix, were purchased from New England BioLabs (Ipswich, MA, USA). DNase I was obtained from Roche Diagnostics GmbH (Mannheim, Germany). DNA purification kits were purchased from Fisher Scientific (Geel, Belgium) and Zymo Research (Irvine, CA, USA). Lysozyme (22500 U/mg) was obtained from Axon Lab AG (Baden, Switzerland). Kanamycin sulfate and ampicillin were obtained from AppliChem GmbH (Darmstadt, Germany). Ni-NTA agarose resin was obtained from Qiagen GmbH (Hilden, Germany). Bradford protein assays were performed using Protein Asaay Dye Reagent Concentrate from Bio-Rad. Dialysis tubing was obtained from Thermo Fisher Scientific (Waltham, MA, USA). Amicon Ultra centrifugal filters were purchased from Merck (Darmstadt, Germany), VivaSpin 500 centrifugal concentrators were purchased from Satorius Stedim Lab (Stonehoude, UK), PD MiniTrap desalting columns were purchased from Cytiva. All buffers were

prepared using Mili-Q water, sterile filtered (0.2 µm membrane filter), pH was adjusted at the temperature the buffer was used. Oligonucleotide synthesis and sequencing was carried out by Microsynth AG (Balgach, Switzerland). Synthetic genes were ordered from ATG Biosynthetics (Merzhausen, Germany). Cell culture reagents including Dulbecco's modified eagle medium (DMEM), OptiMEM, phosphate-buffered saline (PBS), trypsin-EDTA, HBSS and Fetal Bovine Serum (FBS) were purchased from Thermo Fisher Scientific. Transfection reagent Xtreme gene HP was obtained from Roche. ATG7 was purchased form R&D Systems (Boston, USA).

Fmoc-amino acids with suitable side-chain protecting groups, HATU (1-[bis(dimethyl-amino)methylene]-1H-1,2,3-triazolo[4,5-b]pyridinium 3-oxide hexafluorophosphate) were purchased from Merck-Milipore and ChemImpex. HPLC grade CH_3CN from Sigma-Aldrich was used for analytical and preparative HPLC purification. Trifluoroacetic acid for HPLC analytical and preparative HPLC purification was purchased from ABCR. DMF (> 99.8%) from Sigma-Aldrich and N-methylpyrrolidine from ABCR were directly used without further purification for solid phase peptide synthesis. Other commercially available reagents and solvents were purchased from Sigma-Aldrich (Buchs, Switzerland), Acros Organics (Geel, Belgium) and TCI Europe (Zwijndrecht, Belgium).

2.2. Mass spectrometric characterization

High-resolution mass spectra were recorded by the Molecular and Biomolecular Analysis Service (MoBiAS) at ETH Zurich with a Bruker maXis instrument (ESI-MS measurements) equipped with an ESI source and a Q-TOF detector. Reaction monitoring was performed on a Bruker microFLEX instrument (MALDI-TOF) using 4-hydroxy-α-cyanocinnamic acid as the matrix. LC-MS analysis was performed on a Waters Xevo G2-XS QTof. Mass analysis was performed using MassLynx. Sample peaks were integrated and deconvoluted using MaxEnt1.

2.3. RP-HPLC analysis and purification

Peptides were analyzed and purified by reverse phase high performance liquid chromatography (RP-HPLC) on JASCO analytical and preparative instruments equipped with dial pump, mixed and in-line degasser, a variable wavelength UV detector (simultaneous detection of the eluent at 220 nm, 254 nm and 301 nm) and a Rheodyne injector with a 200 µL or 10 mL injection loop. Columns were heated to 60 °C using a Jetstream 2 column heater (analytical) or a H_2O water bath (preparative). The mobile-phases for RP-HPLC were Milipore-H_2O containing 0.1% (v/v) TFA and HPLC grade CH_3CN containing 0.1% (v/v) TFA. Analytical HPLC was performed at a flow rate of 1 mL/min. Analytical columns used were Shiseido Capcell Pak C18 (5 µm, 4.6 mm I.D. x 250 mm) or Agilent Eclipse XDB-C8 (5 µm, 4.6 mm x 150 mm). Preparative HPLC was performed on Shiseido Capcell Pak MGIII (5 µm, 20 mm I.D. x 250 mm) at a flow rate of 40 mL/min.

General *analytical* HPLC methods:

- flow 1 mL/min, isocratic 10% CH_3CN for 3 min, then gradient from 5% to 95% CH_3CN in 14 min

General *preparative* HPLC methods:

- flow 40 mL/min, isocratic 5% CH_3CN for 5 min, then gradient from 5% to 65% CH_3CN in 28 min.

2.4. Fast protein liquid chromatography (FPLC)

Chromatographic protein purification was performed on an Äkta Pure system (GE Healthcare) at 4 °C. Buffers were prepared and pH-adjusted at 4 °C. Buffers were filtered (2 µm) and degassed prior to use. Ion exchange chromatography was performed using MonoS 5/50 GL, or MonoQ 5/50 GL. Size exclusion chromatography was performed on a Superdex 75 Increase 10/300 G, or HiLoad 16/600 Superdex 200. Protein elution was monitored at 280 nm and acquired using Unicorn (6.3.2.89).

2.5. Solid-phase peptide synthesis

Loading of amino acids on solid support was performed as followed:
C-terminal carboxylic acid: The amino acid (1.20 equiv of desired loading) was dissolved in CH_2Cl_2 (200 mM). NMM (2 equiv) was added to the solution. The solution was given to preswollen chloro-trityl resin and shaken for 1h. The resin was washed with CH_2Cl_2 and DMF. Remaining chloro-trityl moieties were capped with CH_2Cl_2/MeOH/NMM (17:2:1, v:v:v) for 1 min. The capping step was repeated once. The resin was washed with CH_2Cl_2 and DMF. The resin was dried using a N_2 stream. The resin was stored at 4 °C.

Peptides were synthesized on a Multisyntech Syro I parallel synthesizer using Fmoc-SPPS chemistry. The following Fmoc amino acids with side-chain protection groups were used: Fmoc-Ala-OH, Fmoc-Arg(Pbf)-OH, Fmoc-Asn(Trt)-OH, Fmoc-Asp(OtBu)-OH, Fmoc-Cys(Trt)-OH, Fmoc-Gln(Trt)-OH, Fmoc-Glu(OtBu)-OH, Fmoc-Gly-OH, Fmoc-His(1-Trt)-OH, Fmoc-Ile-OH, Fmoc-Leu-OH, Fmoc-Lys(Boc)-OH, Fmoc-Lys(Alloc)-OH Fmoc-Nle-OH, Fmoc-Phe-OH, Fmoc-Pro-OH, Fmoc-Ser(tBu)-OH, Fmoc-Thr(tBu)-OH, Fmoc-Trp(Boc)-OH, Fmoc-Tyr(tBu)-OH, Fmoc-Val-OH.

General methods on Multisyntech Syro I parallel synthesizer:

- Amino acids were dissolved in DMF to a concentration of 0.5 M. HATU was dissolved in DMF to a concentration of 0.5 M. DIPEA was dissolved in NMP to a concentration of 2 M. Amino acid, HATU and DIPEA are mixed to a final concentration of 0.2 M, 0.2 M and 0.4 M,

respectively, and added to the resin. The resin was agitated for 45 min. Coupling steps were repeated once.

- Capping was performed with acetic anhydride. 20 vol% acetic anhydride in DMF was mixed with 2 M DIPEA at a ratio of 3:2 and added to the resin. The resin was agitated for 5 min. The capping step was repeated once.
- Fmoc deprotection was performed with 20 vol% piperidine in DMF for 10 min. The deprotection step was repeated once.

2.6. Gel electrophoresis

Sodium dodecyl sulfate-polyacrylamide gel electrophoresis (SDS-PAGE) was carried out on a Mini-PROTEAN Tetra Cell system (Bio-Rad) or a Criterion Cell connected to the PowerPac Basic (Bio-Rad) power supply. Reducing samples were treated with sample buffer (Laemmli 2x or 4x Concentrate, Sigma Aldrich), heated at 95 °C for 5 min and used for separation. A 10-180 kDa pre-stained protein ladder (Thermo Fisher) was applied to at least one well of each gel (4 µL). Samples were separated on 8-16% gradient Mini-PROTEAN TGX Precast gels (Bio-Rad) for 30 minutes at 200 V or 8-16% gradient Criterion TGX Precast gels (Bio-Rad) for 60 min at 180 V at 4 °C. Gels were stained with Coomassie (0.1% Coomassie Brilliant Blue R, 40% MeOH, 10% acetic acid) with agitation for 1h and subsequent destaining (40% MeOH, 10% acetic acid), or with Sypro-Ruby (Bio-Rad) according to the manufacturers procedure. Destained gels were imaged on a Bio-Rad Molecular Imager Pharos FX (Coomassie Blue). Gels were quantified by gel densitometry using ImageLab (Bio-Rad). Gels were cropped in Adobe Illustrator. Full-gels are shown in the Source Data.

2.7. Western blotting

Proteins were electrophoretically transferred from resolved gels to low-fluorescence PVDF membranes using TransBlot (Bio-Rad). For blots anti FLAG, ATG5, ATG16L1 and β-actin, transfer was performed using Mixed MW method (7 min, 1.3 A). For blots anti LC3A/B transfer was performed using Low MW method (5 min, 1.3 A). Blots were blocked with 5 wt% dry skim milk powder in TBS-T for 2 h at room temperature. Blots were washed with TBS-T and then incubated with antibodies at indicated dilutions in TBS-T with 1 wt% BSA for 18 h at 4 °C. Blots were exhaustively washed with TBS-T and incubated with secondary antibody at indicated dilutions in TBS-T with 1 wt% BSA for 1.5 h at room temperature. Blots were exhaustively washed and incubated with Clarity Western ECL (Bio-Rad) and visualized using Bio-Rad Molecular Imager Pharos FX (Chemiluminescence). Blots were cropped in Adobe Illustrator. Full-blots are shown in the Source Data.

2.8. Antibodies

Plasmid	Supplier	Identifier	Dilution
Anti-FLAG	Thermo-Fisher	#MA1-91878	1:1,000
Anti-LC3/AB	Cell-Signalling	#12741	1:1,000
Anti-ATG5	Cell-Signalling	#D5F5U	1:1,000
Anti-ATG16L1	Cell-Signalling	#D6D5	1:1,000
Anti-β-Actin	Thermo-Fisher	#BA3R	1:2,000
Anti-Mouse	Thermo-Fisher	#62-6520	1:3,000
Anti-Goat	Cell-Signalling	#91196	1:3,000

Table S2 Antibodies used in this study.

2.9. Circular dichroism spectroscopy

CD spectra were recorded on a JASCO J-1500 CD spectrometer in a 0.1 cm cuvette. Samples were measured in potassium phosphate (20 mM, pH 7.2) at 20 °C. Data were recorded between 180-300 nm with a measuring speed of 20 nm/min. Measurements were repeated (5x) and averaged. Background spectrum was recorded and subtracted from measured spectra. Data were converted to mean residue ellipticity.

2.10. Gene constructs

Ubiquitin (P0CG48) was synthesized by ATG-Biosynthetics, codon-optimized for *E. coli*. SUMO2 (P61956, Addgene plasmid #53142) was a gift from Dieter Willibold. GABARAP was a gift from Dieter Willibold (O95166, Addgene plasmid #73948), LC3A was a gift from Dieter Willibold (Q9H492, Addgene plasmid #73946), Ube2K C170S (P61086, Addgene plasmid #18892) was a gift from Cecile Pickart, ATG3 was a gift from Robin Ketteler (Q9NT62, Addgene plasmid #129296), SENP1 cat. domain (Q9P0U3, Addgene plasmid #16356) was a gift from Guy Salvesen, ATG7 was a gift from Eiki Kominami and Isei Tanida (O95352, Addgene plasmid #87867). Ubiquitin was subcloned into *Mxe*-GyrA-His$_6$ pET28a (Merck Novagen) using Gibson assembly. GABARAP and LC3A were subcloned into *Mxe*-GyrA-His$_6$ pET28a (Merck Novagen) with an N-terminal SUMO2 solubilizing tag to access protein hydrazides. GABARAP and LC3A were subcloned into pOPINS to express GABARAP and LC3 wild-type proteins. ATG3 was subcloned into pOPINS using Gibson assembly. Mutations were introduced using inverse PCR. All sequences were confirmed by DNA Sanger sequencing (Microsynth, Plasmidsaurus).

Plasmid	Purpose
pET28a Ub(ΔGG)–*Mxe* GyrA-His6	Ub(ΔGG) intein expression to generate Ub(ΔGG)-$NHNH_2$
pET28a SUMO2-GABARAP(ΔG116)–*Mxe* GyrA-His6	SUMO-GABARAP(ΔG116) intein expression to generate GABARAP(ΔG116)-$NHNH_2$
pET28a SUMO2-LC3A(ΔG120)–*Mxe* GyrA-His6	SUMO-LC3A(ΔG116) intein expression to generate LC3A(ΔG116)-$NHNH_2$
pET28 His6-TEV-Ube2K	Ube2K expression
pOPINS His6-SUMO-ATG3	ATG3 wt expression
pOPINS His6-SUMO-ATG3 C264A	ATG3 C264A expression
pOPINS His6-SUMO-ATG3 ΔLIR	ATG3 ΔLIR (Δ90-112) expression
pOPINS His6-SUMO-ATG3 C1	ATG3 expression containing only C264
pOPINS His6-SUMO-ATG3 C1 C264A	ATG3 expression containing no cysteines
pOPINS His6-SUMO-ATG3 C1 ΔLIR	ATG3 ΔLIR (Δ90-112) expression containing only C264
pCMV-3XFLAG-ATG3	Mammalian expression of 3x-FLAG ATG3
pCMV-3XFLAG-ATG3 C264A	Mammalian expression of 3x-FLAG ATG3 C264A
pCMV-3XFLAG-ATG3 ΔLIR	Mammalian expression of 3x-FLAG ATG3 ΔLIR
pCMV-3XFLAG-ATG7	Mammalian expression of 3x-FLAG ATG7

Table S3 Table showing plasmids used in this study. Plasmids for Ala-screen (Fig. 4) are derived from plasmid pOPINS His6-SUMO-ATG3 C1.

2.11. Protein expression

Chemically competent BL21 (DE3) cells (Ub, Ube2K) or Rosetta-CodonPlus (DE3)-RIL (GABARA, LC3A, ATG3, SENP1) were heat-shock transformed with the plasmids and single colonies were used to inoculate overnight precultures in selective lysogeny broth (LB) Miller medium. For Ube2K, ATG3, Ubiquitin-*Mxe*-GyrA, preculture was diluted 1:200 with fresh selective LB Miller medium, cultures were grown in baffled shake flasks at 37 °C. Ub-*Mxe*-GyrA fusion proteins were grown to OD_{600} 0.6. The temperature was reduced to 25 °C and protein expression induced by addition of isopropyl β-D-1-thiogalactopyranoside at a final concentration of 0.5 mM. Expression was allowed to proceed for 18 h. Cells were harvested (4,500 g, 35 min, 4 °C) and resuspended in intein lysis buffer (20 mM Tris, 200 mM NaCl, pH 7.2). Cells were lysed by addition of lysoszyme, DNase I. Cells were further lysed by sonication and suspension clarified by centrifugation (20,000 g, 35 min, 4 °C). Intein fusion proteins were purified by gravity Ni-NTA purification. Resin was washed with excess intein lysis buffer and eluted using intein elution buffer (20 mM Tris, 200 mM NaCl, 300 mM imidazole pH 7.2). The isolated fusion protein was dialyzed against intein cleavage buffer (see below). Ube2K was expressed and purified as previously reported.[1] For SUMO-GABARAP/LC3– *Mxe*-GyrA preculture was diluted 1:200 with fresh autoinduction media. Cells were grown to OD_{600} 1.0, the temperature was reduced to 18 h and cells were grown for an additional 36 h. Cell lysis was performed as for ubiquitin. ATG3 was grown to grown to OD_{600} 0.6. Temperature was reduced to 25 °C and protein expression induced by addition of isopropyl β-D-1-thiogalactopyranoside at a final concentration of 0.5 mM. Cultures were grown for 18 h. Cells were harvested (4,500 g, 35 min, 4 °C) and resuspended in intein lysis buffer (50 mM Tris, 350 mM NaCl, 10 vol% glycerol, 30 mM imidazole, pH 7.4). Cells were lysed by addition of lysoszyme, DNase I and DTT. Cells were further lysed by sonication and

suspension clarified by centrifugation (20,000 g, 35 min, 4 °C). Following Ni-NTA purification. Protein was diluted with 25 mM HEPES pH 7.0 and SENP1 was added to cleave His_6-SUMO. Proteins were further purified by anion exchange chromatography using a MonoQ column (Cytiva) on an Äkta Pure (GE Healthcare) with a gradient of 25 mM HEPES pH 7.0 to 25 mM HEPES, 500 mM NaCl, pH 7.0 over 25 column volumes. Product fractions were pooled and concentrated. For-SUMO2-GABARAP/LC3A wild-tyoe, proteins were expressed according to protocol of intein fusion proteins. After Ni-NTA, SENP1 (1 wt%) was added and protein diluted with 25 mM HEPES pH 7.0. Following complete SUMO-cleavage, GABARAP/LC3A were further purified by anion exchange chromatography using a MonoQ column (Cytiva) on an Äkta Pure (GE Healthcare) with a gradient of 25 mM HEPES pH 7.0 to 25 mM HEPES, 500 mM NaCl, pH 7.0 over 25 column volumes. Protein concentrations were estimated by absorption at 280 nm using extinction coefficients calculated using the online tool ProtParam (https://web.expasy.org/protparam). Proteins were aliquoted, flash frozen in liquid N_2 and stored at –80 °C until use.

2.12. Mammalian cell culture

HEK293T and HCT116 cells were obtained from ATCC. Cells were cultured in DMEM supplemented with FBS and penicillin/streptomycin. Cells were seeded at appropriate densities in 6-well, T25 or T75 flasks. Cells were maintained at 37 °C with 5 vol% CO_2.

3. General methods

3.1. Acylation of hydrazides with anhydrides

Proteins were dissolved or diluted with acylation buffer (100 mM NaPhos, 50 mM NaCl, pH 3.0) to a concentration of 100 µM. The anhydride was dissolved in THF or DMF to a final concentration of 500 mM. The anhydride was added to the desired concentration (1 mM – 20 mM) and mixed. The reaction was allowed to proceed for ca. 5 min. The reaction was analyzed by RP-HPLC or LC-MS analysis. The reaction was purified by dialysis at 4 °C or by buffer exchange using desalting columns (Cytiva). Products could be used without further purification.

For GABARAP(ΔG116)–NHNH α-chloroacetyl 200 equiv of α-chloroacetic acid anhydride were added.

For GABARAP(ΔG116)–NHNH methyl fumarate 85 equiv methyl fumarate anhydride was added.

For LC3A(ΔG120)–NHNH α-chloroacetyl 200 equiv α-chloroacetic acid anhydride was added.

For LC3A(ΔG120)–NHNH methyl fumarate 20 equiv methyl fumarate anhydride was added.

3.2. Generation of Ub hydrazides from Ub-*Mxe* GyrA-His_6 fusions

Ub-*Mxe* GyrA-His_6 fusion after Ni-NTA purification was dialyzed against intein cleavage buffer (25 mM HEPES, 200 mM NaCl, 1 mM EDTA pH 7.2 at 4 °C). After dialysis hydrazine monohydrate (100 mM) was added and the pH adjusted to 7.8. The solution was allowed to stand at room temperature

and monitored using LC-MS analysis. Upon completion the solution was dialyzed against 25 mM HEPES, 200 mM NaCl pH 7.2 at 4 °C. Cleaved intein was removed using gravity Ni-NTA purification. The flow-through was collected and combined with resin washes. The combined fractions were dialyzed and further purified using ion exchange.

3.3. Generation of GABARAP and LC3A hydrazides from SUMO-GABARAP/LC3A-*Mxe* GyrA-His_6 fusions

SUMO-GABARAP/LC3A-*Mxe* GyrA-His_6 fusion after Ni-NTA purification was dialyzed against intein cleavage buffer (25 mM HEPES, 500 mM NaCl, 1 mM EDTA pH 7.2 at 4 °C). After dialysis hydrazine monohydrate (75 mM) and MESNa (10 mM) was added and the pH adjusted to 7.8. The solution was allowed to stand at room temperature and monitored using LC-MS analysis. Upon completion the solution was dialyzed against 25 mM HEPES, 500 mM NaCl pH 7.2 at 4 °C. SENP1 (1 wt%) was added to cleave the SUMO-tag. The cleaved intein was removed using gravity Ni-NTA purification. The flow-through was collected and combined with resin washes. The combined fractions were dialyzed and further purified using cation exchange using a buffer 25 mM Tris pH 8.0 and a gradient of 0–500 M NaCl in the same buffer over 25 column volumes. The product fractions were pooled, concentrated, and stored at –80 °C until further use.

3.4. Synthesis of symmetrical anhydrides

The carboxylic acid was dissolved in CH_2Cl_2 (300 mM) and dicyclohexylcarbodiimide (0.5 eqiv) was added in one portion. The reaction was stirred under N_2 for 18 h. The suspension was filtered and concentrated to ~10% of the initial volume. The solution was placed at 4 °C for 1 h and filtered again. The filtrate was concentrated to obtain the symmetrical anhydride which was used without further purification. Anhydrides can be stored at –20 °C for weeks.

3.5. Reaction of modified Ubl probes with E2s

Ubl probes (15 µM) were mixed with E2s (15 µM) in PBS. The mixture was incubated at 37 °C in a water bath for 0.5-3 h. The reaction was quenched by addition of 2x Laemmli buffer and boiled at 95 °C for 5 min. Samples were resolved by SDS-PAGE (8-16%, Bio-Rad) and visualized by Coomassie staining. If required, gel bands were quantified by gel densitometry using ImageLab (Bio-Rad).

3.6. Competition experiment in the presence of LIR^{ATG3} peptides

ATG3 C1 (15 µM) was mixed with peptides **5** or **6** (1 µM – 1 mM) in PBS. GABARAP methyl fumarate **2** (15 µM) was added and the reaction mixture was incubated at 37 °C for 30 min. The reaction was quenched by addition of 2x Laemmli buffer and boiled at 95 °C for 5 min. Samples were resolved by

SDS-PAGE (8-16%, Bio-Rad) and visualized by Coomassie staining. Gel bands were quantified by gel densitometry using ImageLab (Bio-Rad).

3.7. Alanine-screen

ATG3 C1 (15 µM) was mixed with GABARAP methyl fumarate **2** (15 µM) in PBS, and the reaction mixture was incubated at 37 °C for 30 min. The reaction was quenched by addition of 2x Laemmli buffer and boiled at 95 °C for 5 min. Samples were resolved by SDS-PAGE (8-16%, Bio-Rad) and visualized by Coomassie staining. Gel bands were quantified by gel densitometry using ImageLab (Bio-Rad).

3.8. NaCl dependence

ATG3 C1 (15 µM) was mixed with phosphate buffer with varying concentrations of NaCl (0-500 mM). GABARAP methyl fumarate **2** (15 µM) was added and the reaction mixture was incubated at 37 °C for 30 min. The reaction was quenched by addition of 2x Laemmli buffer and boiled at 95 °C for 5 min. Samples were resolved by SDS-PAGE (8-16%, Bio-Rad) and visualized by Coomassie staining. Gel bands were quantified by gel densitometry using ImageLab (Bio-Rad).

3.9. Structure prediction

The ATG3-GABARAP complex was predicted using the publicly available ColabFold work-book (https://colab.research.google.com/github/sokrypton/ColabFold/blob/main/AlphaFold2.ipynb). ATG3 sequence (Q9NT62) and mature-GABARAP (O95166) were used as input sequences. Structure prediction was performed in no-template mode with msa_mode "MMseqs2 (UniRef+Environmental)" and pair_mode "unpaired+paired".

3.10. Synthesis of linear peptides

Amino acids were loaded according to the general methods. Automated peptide elongation was carried out on a Multisyntech Syro I parallel synthesizer according to the general peptide methods. After coupling of the final amino acid Fmoc group was removed by treatment with 20 vol% piperidine. The resin was thoroughly washed. Acetic anhydride (2 equiv) and DIPEA (4 equiv) in DMF were added to the resin and agitated for 2h. The peptide was cleaved form the resin using TFA/DODT/H_2O (95:2.5:2.5, v/v) for 1 h. The resin was removed by filtration and the filtrate concentrated under reduced pressure. The solution was triturated with Et_2O and centrifuged to obtain crude peptide. The crude peptide was dissolved in H_2O/CH_3CN (1:1, v/v) + 0.1% (v/v) TFA and purified using preparative HPLC.

3.11. Synthesis of cyclic peptides

Amino acids were loaded according to the General Methods. Automated peptide elongation was carried out on a Multisyntech Syro I parallel synthesizer according to the general peptide methods. After coupling of the final amino acid Fmoc group was removed by treatment with 20 vol% piperidine. The resin was thoroughly washed. α-Chloroacetic anhydride (2 equiv) and DIPEA (4 equiv) in DMF were added to the resin and agitated for 2h. The resin was thoroughly washed. The peptide was cleaved form the resin using TFA/DODT/H_2O (95:2.5:2.5, v/v) for 1 h. The resin was removed by filtration and the filtrate concentrated under reduced pressure. The solution was triturated with Et_2O and centrifuged to obtain crude peptide. The peptide was dissolved in 6 M Gdn HCl, 200 mM Na_2CO_3 (pH 9.5, 20 mL, 5 mM with respect to initial resin loading) and shaken at rt for 1 h. The reaction mixture was diluted with H_2O/CH_3CN (1:1, v/v) + 0.1% (v/v) TFA and purified using preparative HPLC.

3.12. Synthesis of fluorescently labelled peptides

Fmoc-Lys(alloc)-OH was loaded according to the General Methods. Automated peptide elongation was carried out on a Multisyntech Syro I parallel synthesizer according to the general peptide methods. The side-chain alloc group was removed by treatment with $Pd(PPh_3)_4$ (0.25 equiv) and $PhSiH_3$ (24 equiv) in CH_2Cl_2. The deprotection step was repeated once. The resin was thoroughly washed. FITC (2 equiv) and DIPEA (4 equiv) were added in DMF and shaken for 2 h at rt. All steps following FITC coupling were performed in the dark. The resin was removed by filtration and the filtrate concentrated under reduced pressure. The solution was triturated with Et_2O and centrifuged to obtain crude peptide. The crude peptide was dissolved with H_2O/CH_3CN (1:1, v/v) + 0.1% (v/v) TFA and purified using preparative HPLC.

3.13. Fluorescence polarization measurement

Fluorescent peptides were dissolved in DMSO and PBS. The concentration was determined by UV-absorption and extinction coefficient at 280 nm. The peptides were diluted to a 2X concentration of 20 nM using PBS. Protein concentration was determined using UV-absorption and extinction coefficient at 280 nm. Peptides and proteins were mixed 1:1 to a final peptide concentration of 5 nM and protein concentrations of 15, 10, 5, 1.5, 1.0, 0.75, 0.5, 0.375, 0.25, 0.1875, 0.125, 0.094, 0.063, 0.047 µM. The reactions were incubated at room temperature for 45 min. Fluorescence polarization and intensity was measured on a Victor Nivo Plate Reader (Perkin Elmer). Fluorescence polarization was plotted against concentration and the binding curve was fitted in PRISM (GraphPad) using least square regression to extract binding affinities. The measurements were performed in triplicates and plotted as such. Errors were estimated based on these triplicates.

3.14. Co-crystallization of GABARAP with LIR^{ATG3} peptide

GABARAP–OH in 25 mM Tris, 150 mM NaCl, pH 8.0 was concentrated using centrifugal filters (Amicon) to 730 µM ATG3 LIR peptide (Y90-H112) was resuspended in DMSO (50 mM) and 3 equivalents with respect to GABARAP were added. The reaction was dialyzed against 25 mM Tris, 150 mM NaCl, pH 8.0 at room temperature for 20 h. Crystallization was performed using hanging-drop vapour diffusion in 24-well crystallization flasks (Hampton Research). Protein solution (2 µL) was mixed with reservoir solution (2 µL) on siliconized cover-slips (Hampton Research). Crystallizations were performed at room temperature. Crystals were observed after 2 days. Crystals were obtained for 0.2 M NaOAc, 0.1 M sodium cacodylate, 30 vol% PEG 8,000, pH 6.5. Crystals were cryo-protected with 25 vol% glycerol and flash-frozen. Crystal diffraction was measured at the Swiss Light Source (Paul Scherrer Institute, Villigen, Switzerland) beamline X06DA (PXIII) equipped with the PILATUS 2M-F detector system (Dectris, Switzerland) at a wavelength of 1.0 Å, while the crystal was kept at 100 K. XDS software was used for data processing[2]. Molecular replacement using the GABARAP structure from PDB structure 6HB9 as the search model was performed using Phaser[3, 4]. The asymmetric unit contained eight copies of the GABARAP–LIR^{ATG3} peptide complex. Refinement was performed iteratively using phenix.refine from the Phenix[5] software package and Coot[6]. Protein structure visualization and analysis (RMSD, Coulombic potential, surface area) was performed using the UCSF ChimeraX software package[7].

3.15. Generation of HCT116 ATG3 knock-down cell-line using CRISPRi

HCT116 cells expressing dCas9-KRAB were prepared according to published procedures.[8] sgRNA were incubated with T4 polynucleotide kinase (NEB) at 37 °C for 30 min and then annealed by incubation at 95 °C for 5 min and cooling to 25 °C at 5 °C/min. Annealed sgRNAs were cloned into pCRISPRia–v2 (Addgene #84832) using BstXI and BlpI restriction enzyme sites. Correct insertion was validated by Sanger sequencing.

Lentiviral packaging was performed in HEK293T cells using Xtreme-Gene HP (Roche) transfection reagent according the manufacturer's procedure. pVSVG (Addgene #8454), pR8.2 dvpr (Addgene #8455) and pCRISPRia–v2 carrying the sgRNA of interest were transfected using a ration of 4:1:3.5 (w/w/w). The media was changed after 24 h and lentiviral supernantant harvested after an additional 24 h. HCT116 dCas9-KRAB cells were transfected with lentiviral supernatant in the presence of 10 µg/mL polybrene. After 24 h cell media was changed. After additional 24 h, selection was initiated by supplementing cell media with 2.5 µM puromycin. Selection was performed for additional 3 d until all non-transfected control cells had died. ATG3 knock-down was confirmed using Anti-ATG3 Western blotting. sgRNA 1 showed most efficient knock-down and was used for all experiments. Used primer pairs are listed in Supplementary Table S5

3.16. Generation of HEK293T ATG3 -/- using CRISPR-Cas9

ATG3-deficient HEK293T cell lines were derived by nucleofection of recombinant Cas9 and in vitro transcribed (IVT) sgRNAs as described previously[9]. In brief, IVT templates were generated by extension PCR using Phusion Polymerase (New England Biolabs) with oligonucleutides T7FwdAmp (2 µM), T7 RevAmp (2 µM), T7RevLong (0.02 µM) and one of two oligonucleotides bearing an ATG3-targeting protospacer (T7FwdVar_sgATG3.KO3 and T7FwdVar_sgATG3.KO4, each 0.02 µM). Protospacers were chosen based on previously optimized design rules[10]. Crude PCR products were subjected to IVT (HiScribe T7 High yield RNA Synthesis kit, New England Biolabs, 37°C overnight), dephosphorylated (Quick CIP, New England Biolabs, 5U per reaction, 37°C, 30 min) and column purified (RNeasy Mini kit, QIAGEN).

120 pmol IVT product were complexed with 100 pmol recombinant His_6-Cas9-NLS (kindly provided by the Genome Engineering and Measurement Lab, GEML, Functional Genomics Center Zürich) for 20 min at room temperature in RNP assembly buffer (150 mM KCl, 20 mM HEPES, 1mM $MgCl_2$, pH 7.5) followed by delivery using a 4D-nucleofector device (Lonza). Sub-clonal cell lines were isolated by cell sorting (SONY SH-800). Genomic DNA of resulting clonal cell lines was isolated (QuickExtract, Lucigen) followed by PCR of target regions with Q5 Polymerase (New England Biolabs) using one of two primer pairs (sgATG3.KO3_seq or sgATG3.KO4_seq). Homozygous knockout lines were identified by sanger sequencing and deconvolution of composite chromatograms (ICE Analysis Tool, Synthego). All used primer pairs are listed in Supplementary Table S6.

3.17. *In cellulo lipidation* of LC3A/B

HEK293T ATG3 -/- cells were grown in a 6-well plate. pCMV-3xFLAG-ATG3 plasmids containing ATG3 wt, C264A or ΔLIR were transfected using Xtreme-Gene HP (Roche) in OptiMem. After 24 h media was changed to DMEM. After an additional 24 h the media was changed to HBSS supplemented with 40 µM chloroquine and incubated for 60 min. Cells were directly lysed using RIPA-buffer (50 mM Tris, 150 mM NaCl, 1 vol% Nonidet P-40, 0.5 wt% deoxycholate, 0.1 wt% SDS, pH 7.5) in the presence of protease inhibitors (Thermo-Fisher). Insoluble cell components were removed by centrifugation. Protein concentrations were measured by BCA according to the manufacturers protocol. Cell lysates were diluted to equal protein concentrations using RIPA-buffer. 10 µg of total protein was loaded and used for SDS-PAGE and Western bot analysis.

3.18. Co-immunoprecipitation of ATG3

HCT116 ATG3 knock-down cells, generated by CRISPR inhibition, were grown in T25 flasks. pCMV-3xFLAG-ATG3 plasmids containing ATG3 wt, C264A or ΔLIR were transfected using Xtreme-Gene HP (Roche) in OptiMem. After 24 h media was changed to DMEM. After an additional 24 h the media was changed to HBSS supplemented with 40 µM chloroquine and incubated for 60 min. Cells were

directly lysed using NP-40 buffer (50 mM Tris, 150 mM NaCl, 1 vol% nonidet-P40, pH 8.0) in the presence of protease inhibitors (Thermo-Fisher). Insoluble cell components were removed by centrifugation. An aliquot of lysate was removed for further analysis. The remaining cell lysate was incubated with Anti-FLAG M2 magnetic beads (20 µL, Sigma-Aldrich) for 2 h at 4 °C. The resin was washed with NP40 lysis buffer and PBS. Proteins were eluted by addition of 4x non-reducing Laemmli buffer and boiling at 95 °C for 5 min. β-mercaptoethanol was added to the eluted proteins. Input and elution were analyzed by SDS-PAGE and Western blot analysis.

3.19. Analysis of *in cellulo* ATG3 thioester formation

HEK293T ATG3 -/- cells were grown in a 6-well plate. pCMV-3xFLAG-ATG3 plasmids containing ATG3 wt, C264A or ΔLIR were transfected using Xtreme-Gene HP (Roche) in OptiMem. After 24 h media was changed to DMEM. After an additional 24 h the media was changed to HBSS supplemented with 40 µM chloroquine and incubated for 60 min. Cells were directly lysed using NP-40 buffer (50 mM Tris, 150 mM NaCl, 1 vol% nonidet-P40, pH 8.0) in the presence of protease inhibitors (Thermo-Fisher). Insoluble cell components were removed by centrifugation. Aliquots of cell lysate were removed and directly quenched by the addition of either 4x reducing Laemmli buffer or 4x non-reducing Laemmli buffer. Samples containing reducing Laemmli buffer were further boiled for 5 min at 95 °C. Samples were analyzed by SDS-PAGE and Western blot analysis.

3.20. ATG3 pull-down using 3xFLAG-ATG7

HCT116 cells were grown in T25 flasks. pCMV-3xFLAG-ATG7 plasmid was transfected using Xtreme-Gene HP (Roche) in OptiMem. After 24 h media was changed to DMEM and incubated for additional 24 h. Cells were directly lysed using NP-40 buffer (50 mM Tris, 150 mM NaCl, 1 vol% nonidet-P40, pH 8.0) in the presence of protease inhibitors (Thermo-Fisher). Insoluble cell components were removed by centrifugation. Cell lysate was incubated with Anti-FLAG M2 magnetic beads (40 µL, Sigma-Aldrich) for 2 h at 4 °C. The resin was washed with NP40 lysis buffer and PBS. Recombinant ATG3 variants (wt, ΔLIR, W107A, 15 µM) were added to immobilized ATG3 resin and incubated for 1 h at 4 °C. The resin was washed with NP40 lysis buffer and PBS. Proteins were eluted by addition of 4x non-reducing Laemmli buffer and boiling at 95 °C for 5 min. β-mercaptoethanol was added to the eluted proteins. Input and elution were analyzed by Sypro-Ruby staining and SDS-PAGE analysis.

3.21. Preparation of Fluorescein-GABARAP

Cysteine-GABARAP was expressed as a His-SUMO linear fusion as described above. Following SUMO-tag cleavage, GABARAP was concentrated to 100 µM and mixed with fluorescein-iodoacetamide in DMF (3 mg) and incubated at room temperature. Reaction progress was monitored using LC-MS analysis. After 3 h excess small molecule was removed by buffer exchange to PBS using MidiPrep desalting columns (Cytiva). Buffer exchange was repeated (2x) to provide fluorescein-GABARAP.

3.22. Pulse-chase assay of GABARAP transfer to ATG3

Fluorescein-GABARAP (10 µM) was mixed with ATG7 (1 µM) in 25 mM Tris, 150 mM NaCl, pH 7.5. ATP-$MgCl_2$ (5 mM) was added and the mixture was incubated at 4 °C for 15 min. The solution was diluted 3-fold with 25 mM Tris, 150 mM NaCl, 50 mM EDTA, pH 7.5 and buffer exchanged using a Zeba-desalting column to 25 mM Tris, 150 mM NaCl, 50 mM EDTA, pH 7.5. The solution was added to recombinant ATG3 (0.5 µM) variants and incubated for 5 min. Aliquots were removed after 1, 2.5 and 5 min and mixed with either 4x non-reducing Laemmli buffer or 4x reducing Laemmli buffer. Samples were resolved using SDS-PAGE and analyzed by in-gel fluorescence and Stain-free visualization. ATG3~GABARAP band was quantified by gel densitometry (Image-Lab, BioRad) and normalized to ATG3~GABARAP band for wild-type ATG3 at 5 min.

4. Characterization of peptides and proteins

4.1. Linear LIR^{ATG3} peptide 5

Leu-Glu-Ala-Ile-Ile-Glu-Glu-Asp-Asp-Gly-Asp-Gly-Gly-Trp-Val-Asp-Thr-Tyr-His-Ala-Gly-Gly-Gly-Lys

HRMS (ESI): calculated for $[C_{110}H_{162}N_{28}O_{42}]^{2+}$: m/z 1273.5695, found: m/z 1273.5712

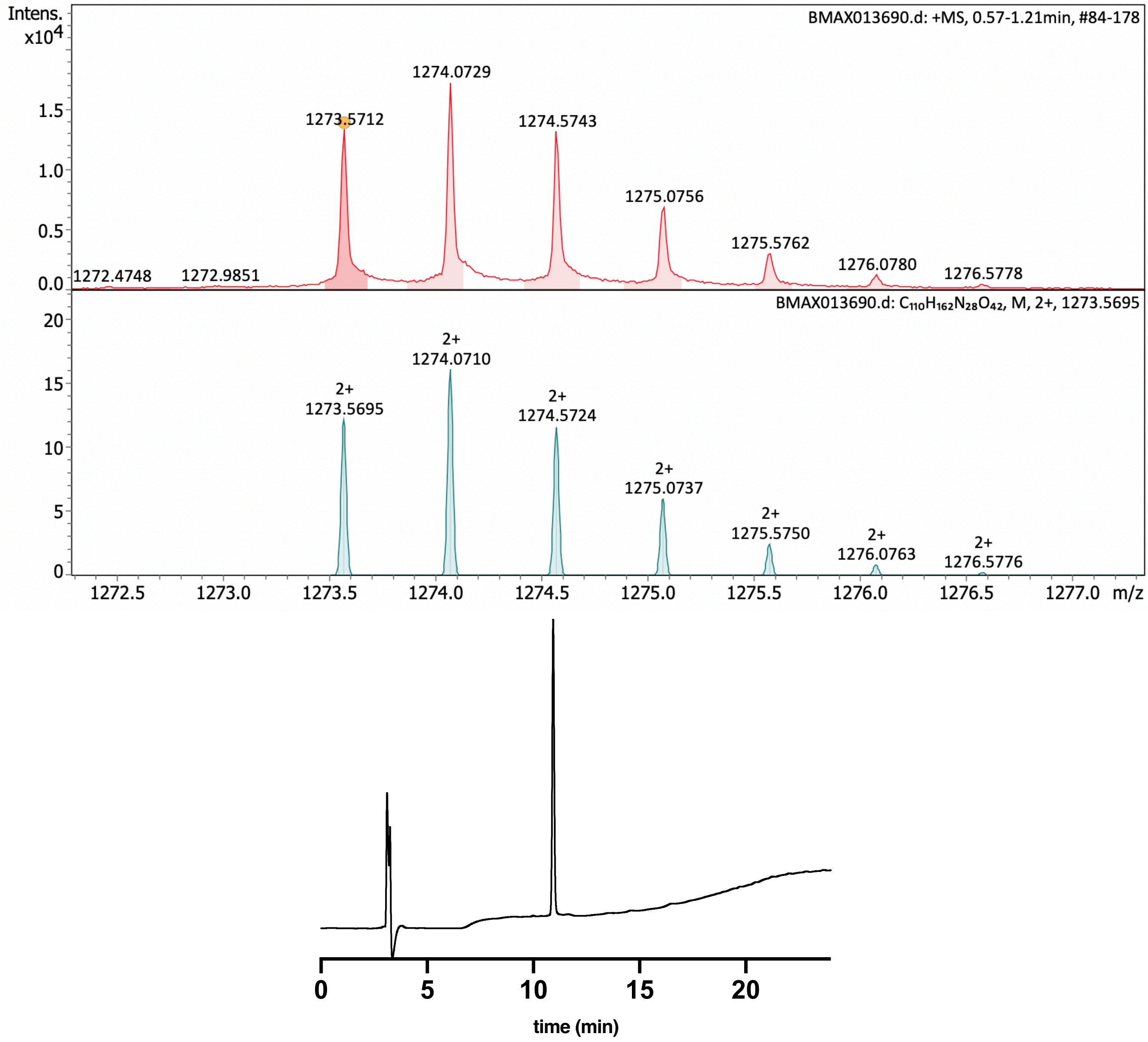

Characterization of peptide 5. HRMS (ESI) spectrum of purified peptide showing recorded mass spectrum (upper panel) and calculated spectrum (lower panel). Analytical RP-HPLC trace of **5**. Absorption at 220 nm is shown.

4.2. Cyclic LIR^{ATG3} peptide 6

Leu-Glu-Ala-Ile-Ile-Glu-Glu-Asp-Asp-Gly-Asp-Gly-Gly-Trp-Val-Asp-Thr-Tyr-His-Cys

HRMS (ESI): calculated for $[C_{95}H_{134}N_{22}O_{37}S]^{2+}$: m/z 1103.4495, found: m/z 1103.4492

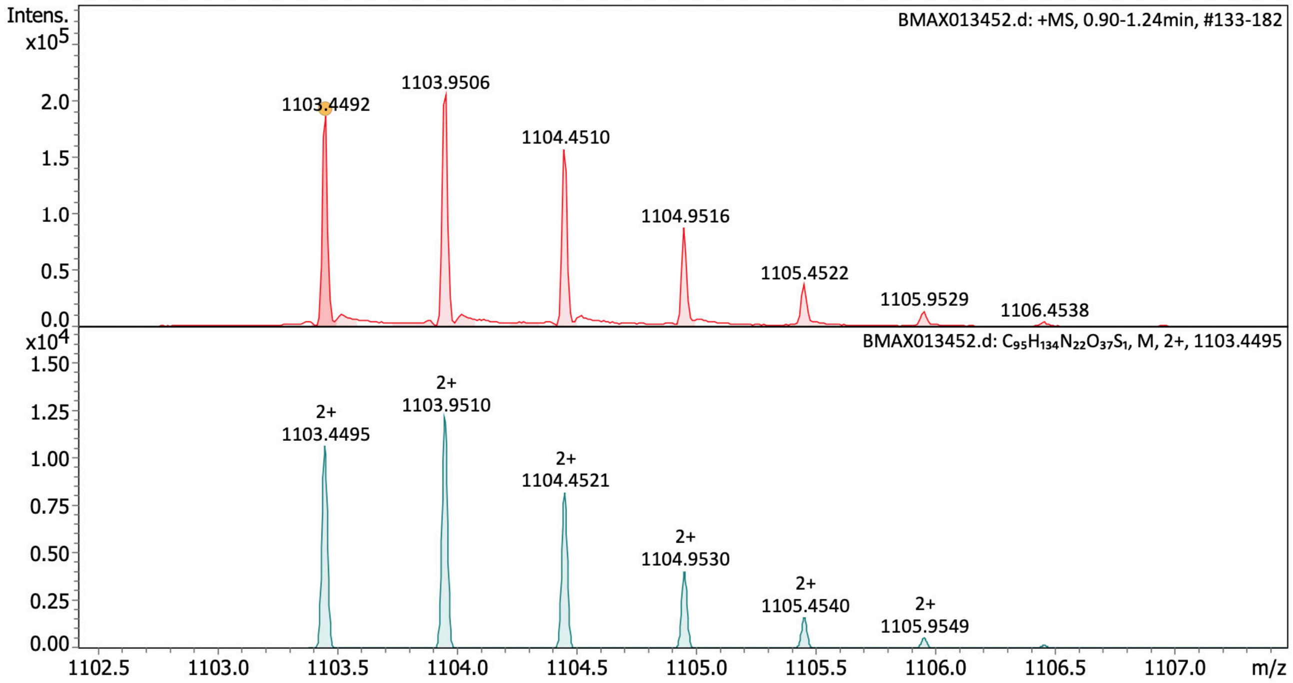

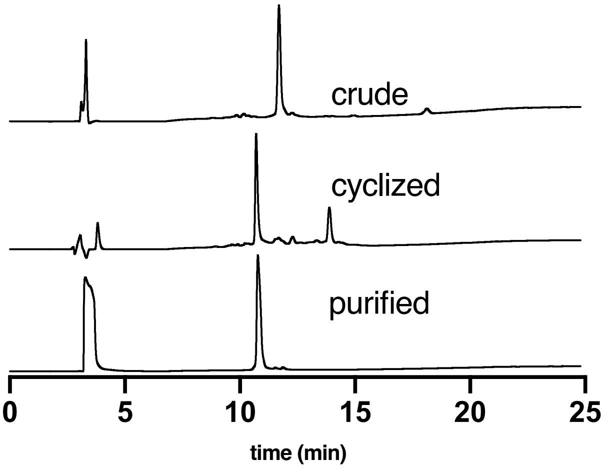

Characterization of peptide 6. HRMS (ESI) spectrum of purified peptide showing recorded mass spectrum (upper panel) and calculated spectrum (lower panel). RP-HPLC of linear peptide after resin cleavage, crude RP-HPLC trace during peptide cyclization, RP-HPL of purified cyclized peptide **6**. Absorption at 220 nm is shown.

4.3. Fluorescent linear LIR^{ATG3} peptide

Leu-Glu-Ala-Ile-Ile-Glu-Glu-Asp-Asp-Gly-Asp-Gly-Gly-Trp-Val-Asp-Thr-Tyr-His-Ala-Gly-Gly-Gly-Lys

The peptide was obtained as a white solid.
HRMS (ESI): calculated for $[C_{131}H_{173}N_{29}O_{47}S]^{2+}$: m/z 1468.0874, found: m/z 1468.0909

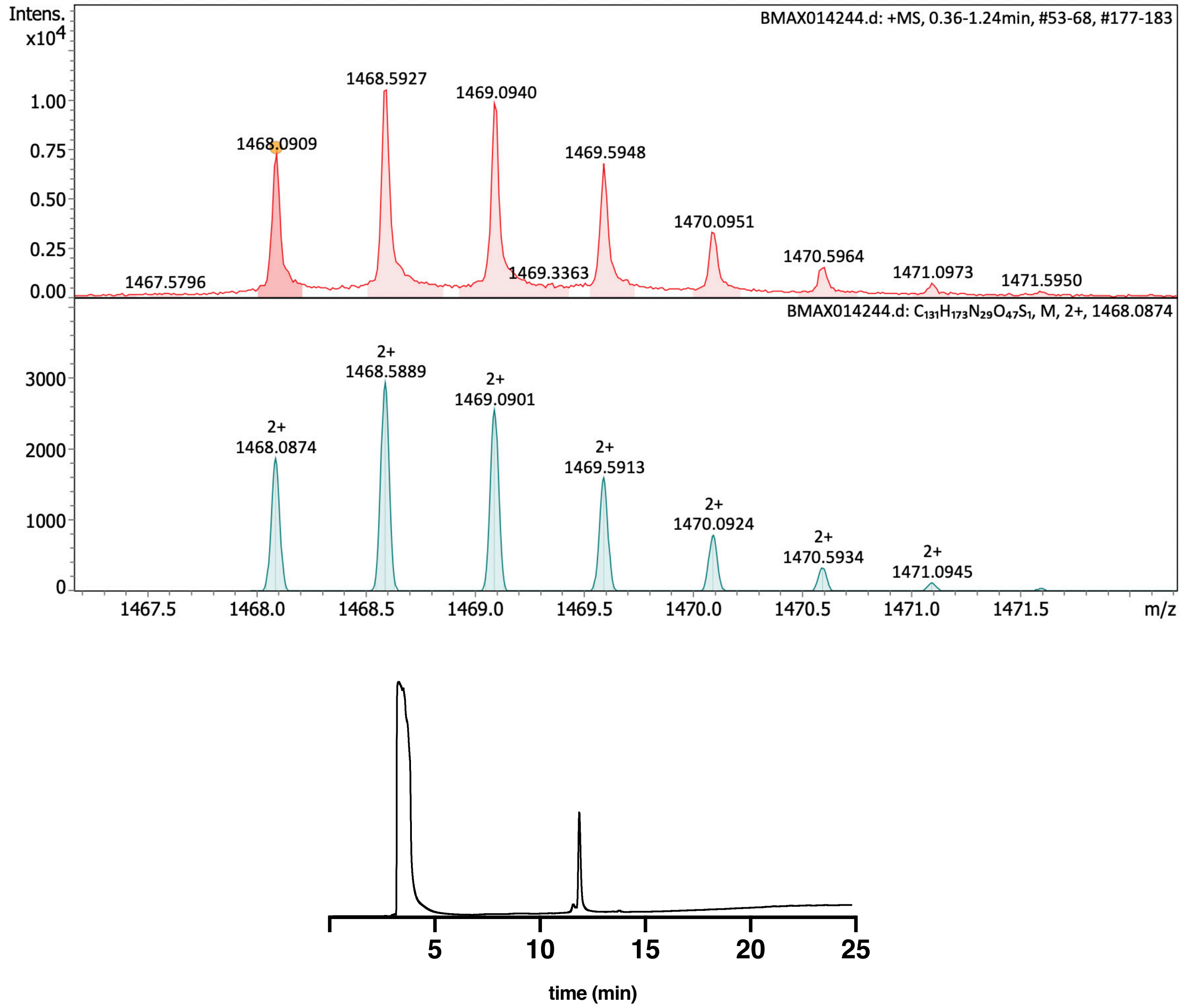

Characterization of fluorescent linear LIR^{ATG3} peptide. HRMS (ESI) spectrum of purified peptide **5** showing recorded mass spectrum (upper panel) and calculated spectrum (lower panel). Analytical RP-HPLC trace of purified peptide. Absorption at 220 nm is shown.

4.4. Fluorescent cyclic LIR[ATG3] peptide

Leu-Glu-Ala-Ile-Ile-Glu-Glu-Asp-Asp-Gly-Asp-Gly-Gly-Trp-Val-Asp-Thr-Tyr-His-Cys-Gly-Gly-Gly-Lys

HRMS (ESI): calculated for $[C_{131}H_{171}N_{29}O_{47}S2]^{2+}$: m/z 1483.0656, found: m/z 1483.0690

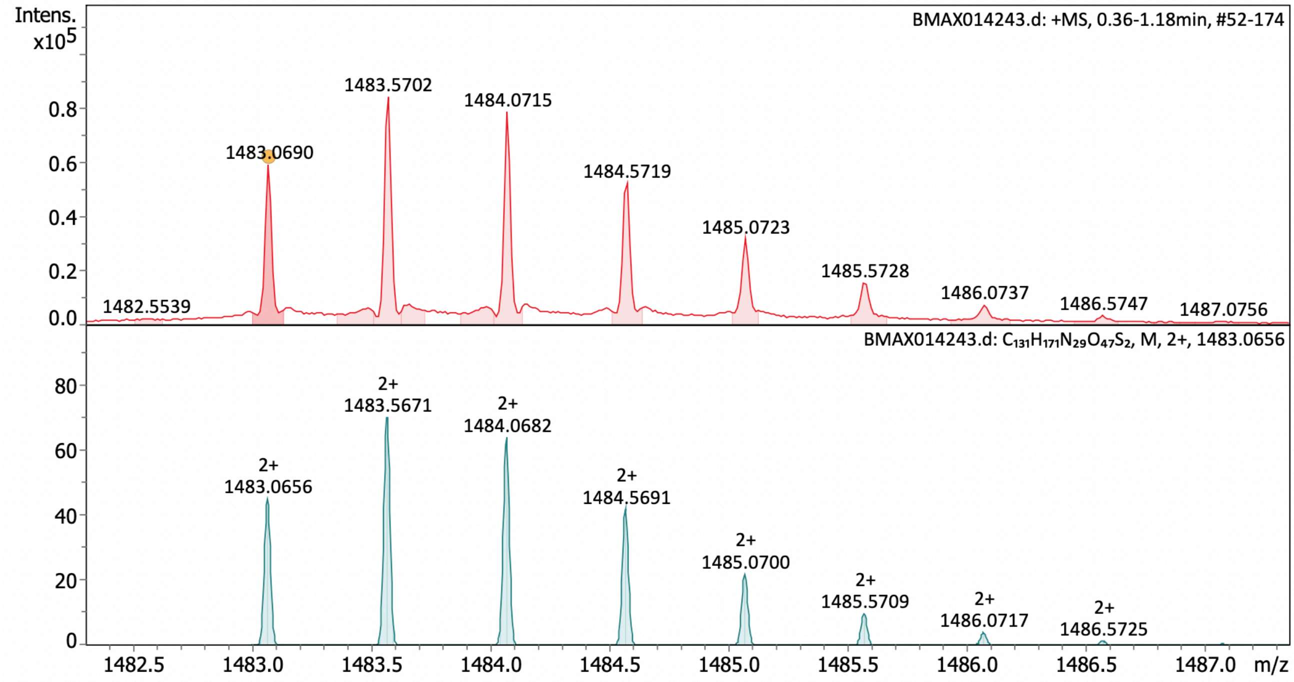

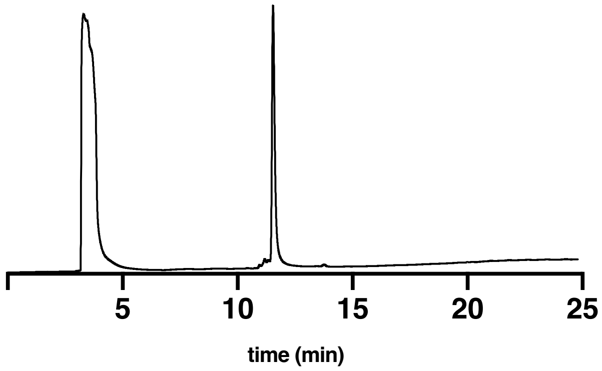

Characterization of fluorescent cyclic LIR[ATG3] peptide. HRMS (ESI) spectrum of purified peptide showing recorded mass spectrum (upper panel) and calculated spectrum (lower panel). Analytical RP-HPLC trace of purified peptide. Absorption at 220 nm is shown.

4.5. Fluorescent truncated peptide LIR^{ATG3} peptide

Gly-Asp-Gly-Gly-Trp-Val-Asp-Thr-Tyr-His-Ala-Gly-Gly-Gly-Lys

The peptide was obtained as a white solid.
HRMS (ESI): calculated for $[C_{87}H_{103}N_{20}O_{28}S+H]^+$: m/z 1907.6966, found: m/z 1907.6973

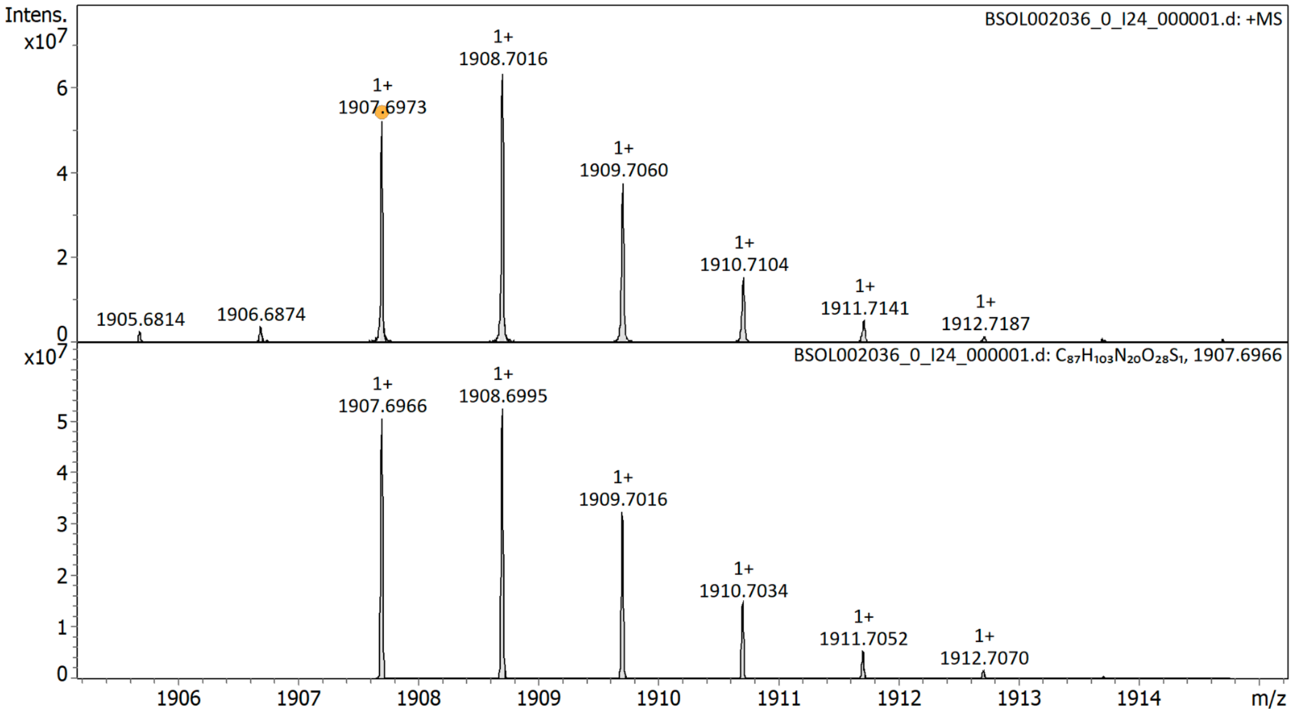

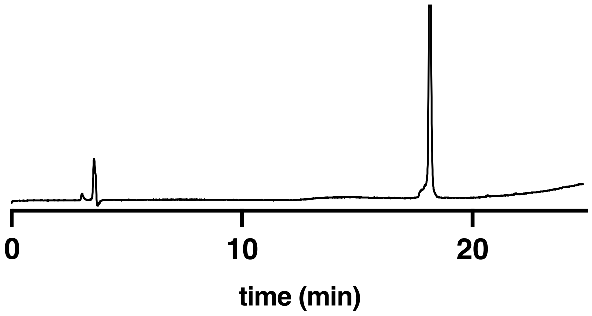

Characterization of fluorescent truncated LIR^{ATG3} peptide. HRMS (ESI) spectrum of purified peptide showing recorded mass spectrum. Analytical RP-HPLC trace of purified peptide. Absorption at 220 nm is shown.

4.6. LIR^{ATG3} peptide for co-crystallization

Tyr-Ser-Asp-Glu-Leu-Glu-Ala-Ile-Ile-Glu-Glu-Asp-Asp-Gly-Asp-Gly-Gly-Trp-Val-Asp-Thr-Tyr-His-Gly

The peptide was obtained as a white solid (30 mg, 11%).
HRMS (ESI): calculated for $[C_{116}H_{163}N_{27}O_{47}]^{2+}$: m/z 1343.0592, found: m/z 1343.0592

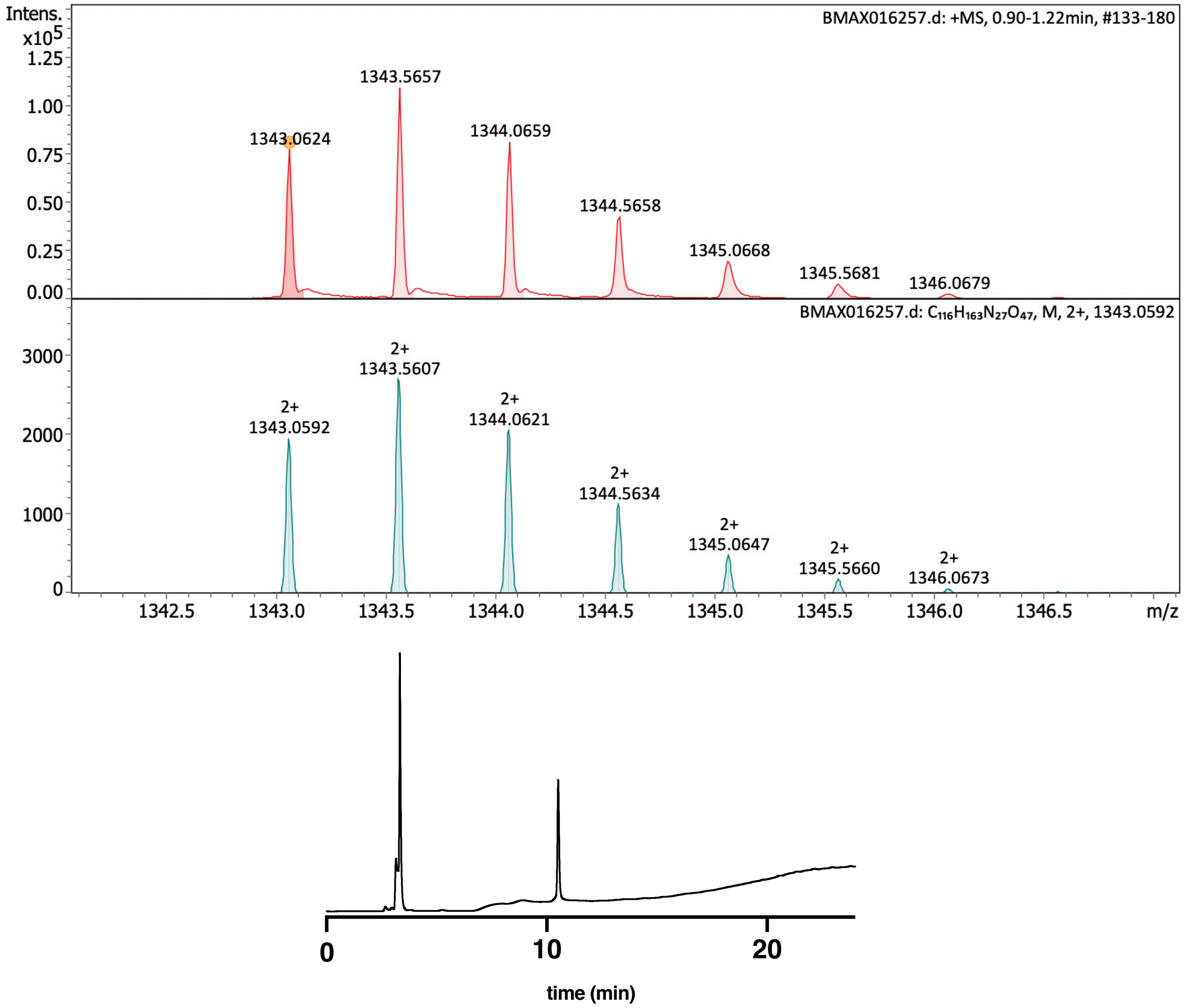

Characterization of LIR^{ATG3} peptide. HRMS (ESI) spectrum of purified peptide showing recorded mass spectrum (upper panel) and calculated spectrum (lower panel). Analytical RP-HPLC trace of purified peptide. Absorption at 220 nm is shown.

4.7. GABARAP

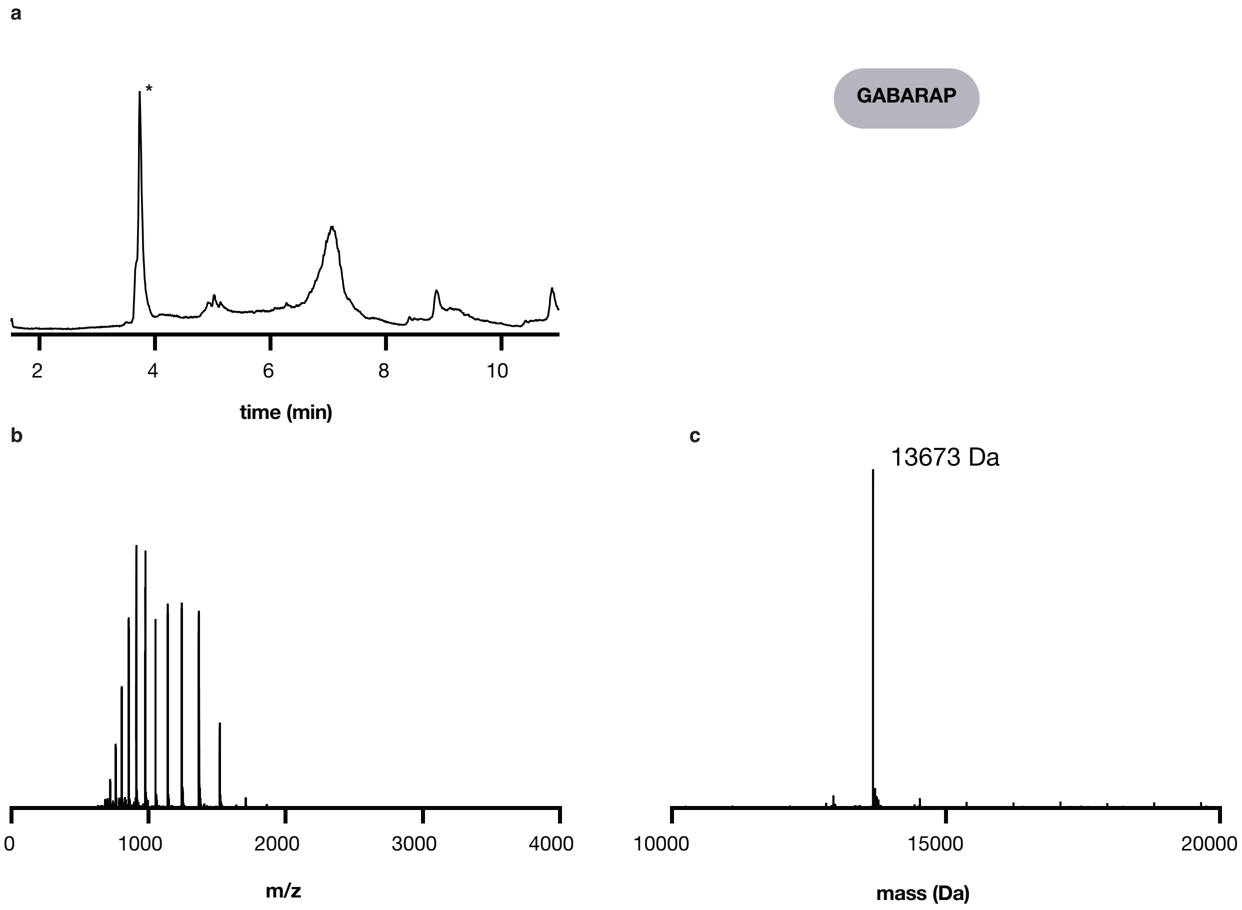

Characterization of GABARAP. **a** LC-chromatogram of recombinantly expressed GABARAP. TIC is shown. Protein peak is marked with a star. **b** Mass-spectrum (ESI) of recombinantly expressed GABARAP. **c** Deconvoluted mass spectrum of GABARAP. Expected mass 13673 Da.

4.8. GABARAP(ΔG116)–$NHNH_2$

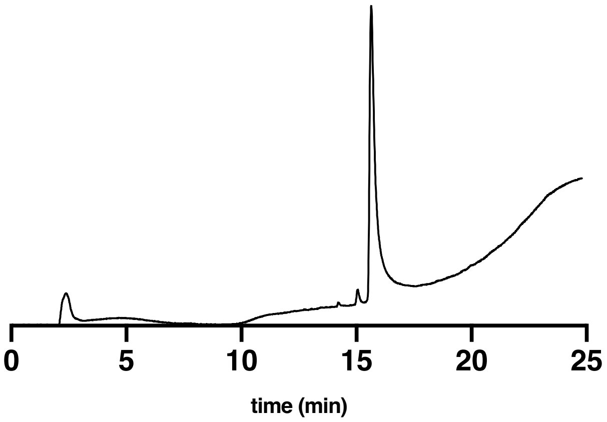

HRMS (ESI): calculated for $[C_{631}H_{965}N_{165}O_{171}S]^{+}$: m/z 13622.1608, found: m/z 13622.2880

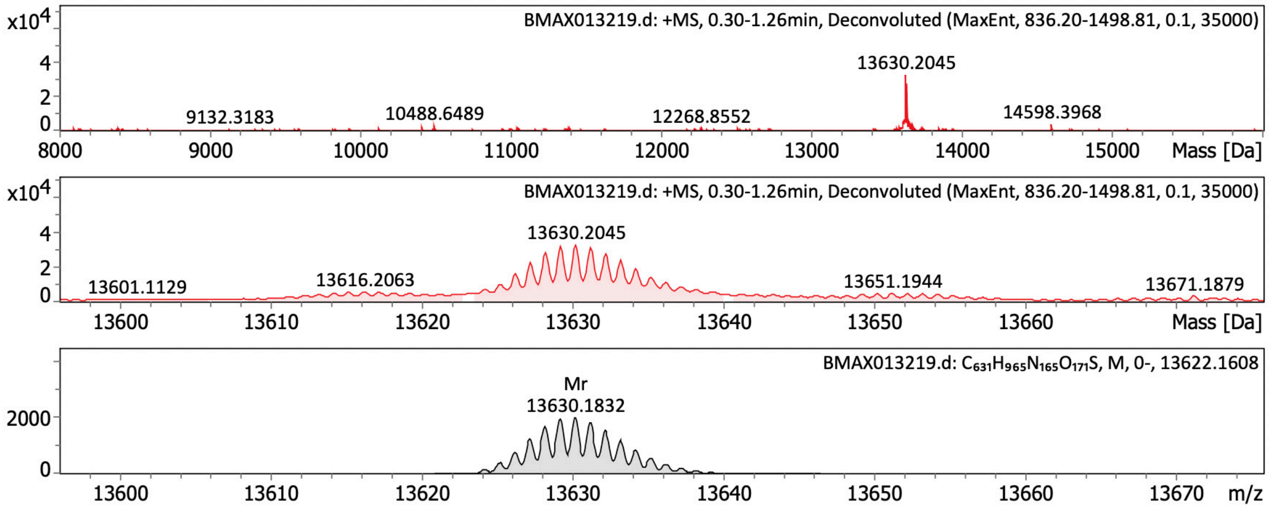

Characterization of GABARAP(ΔG116)–$NHNH_2$. Analytical RP-HPLC of purified GABARAP ΔG116 $NHNH_2$. UV absorption at 220 nm is shown. HRMS (ESI) spectrum of GABARAP ΔG116 $NHNH_2$ showing recorded mass spectrum (upper panel), zoom in of recorded spectrum (middle panel) and calculated spectrum (lower panel).

4.9. GABARAP(ΔG116)–NHNH α-chloroacetyl 1

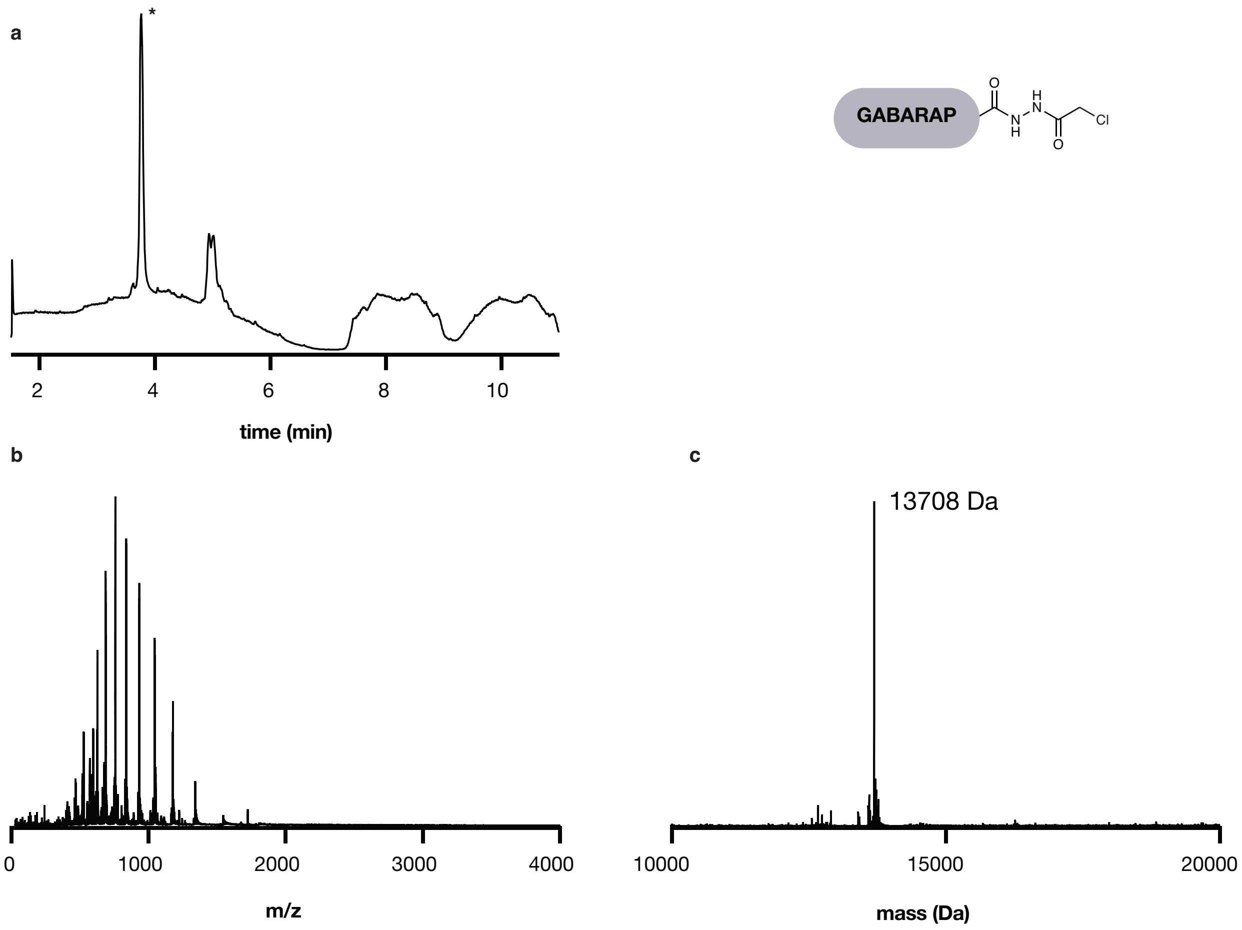

Characterization of GABARAP(ΔG116)–NHNH α-chloroacetyl. **a** LC-chromatogram of GABARAP(ΔG116)–NHNH α-chloroacetyl. TIC is shown. Protein peak is marked with a star. **b** Mass-spectrum (ESI) of GABARAP(ΔG116)–NHNH α-chloroacetyl. **c** Deconvoluted mass spectrum of GABARAP(ΔG116)–NHNH α-chloroacetyl. Expected mass 13707 Da.

4.10. GABARAP(ΔG116)–NHNH methyl fumarate 2

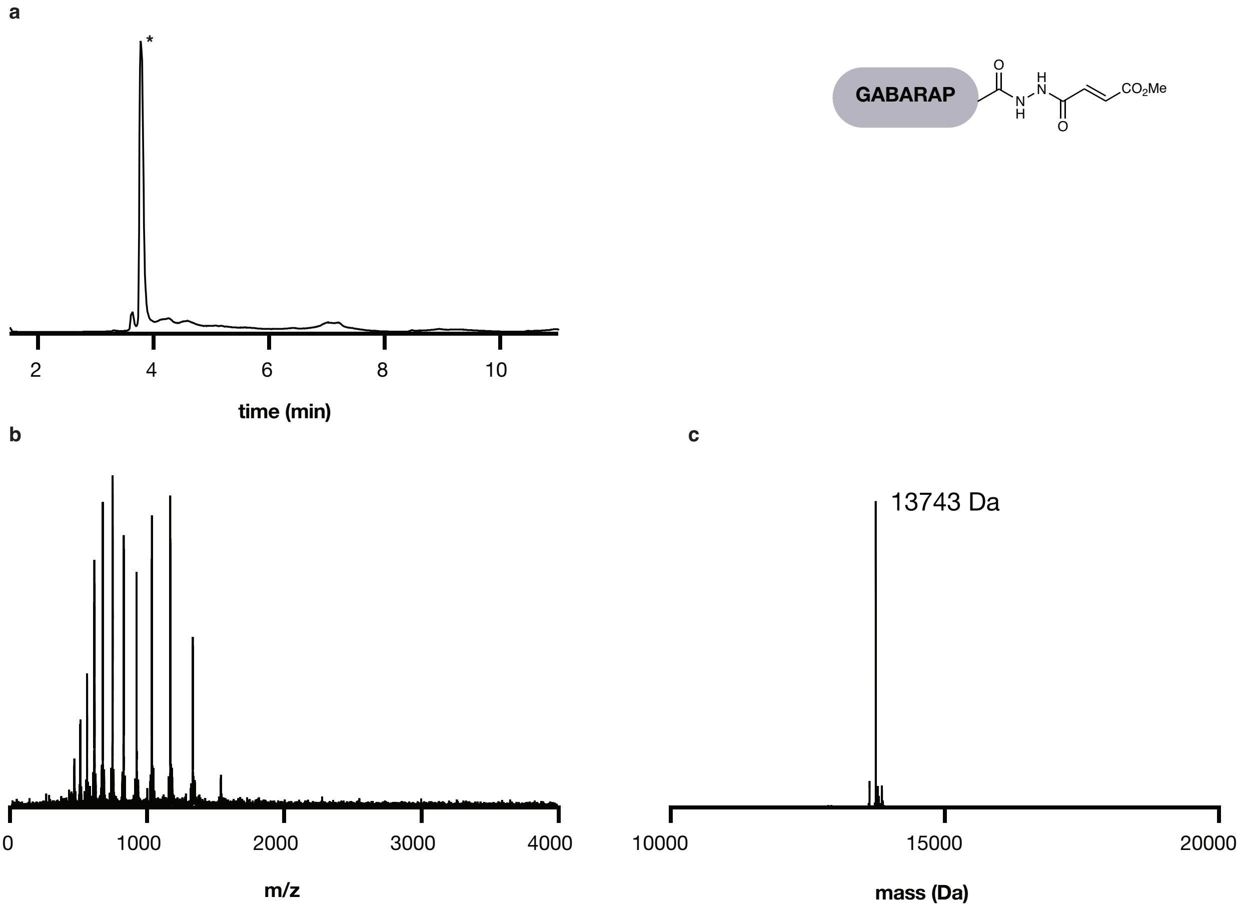

Characterization of GABARAP(ΔG116)–NHNH methyl fumarate. a LC-chromatogram of GABARAP(ΔG116)–NHNH methyl fumarate. TIC is shown. Protein peak is marked with a star. **b** Mass-spectrum (ESI) of GABARAP(ΔG116)–NHNH methyl fumarate. **c** Deconvoluted mass spectrum of GABARAP(ΔG116)–NHNH methyl fumarate. Expected mass 13742 Da.

4.11. LC3A

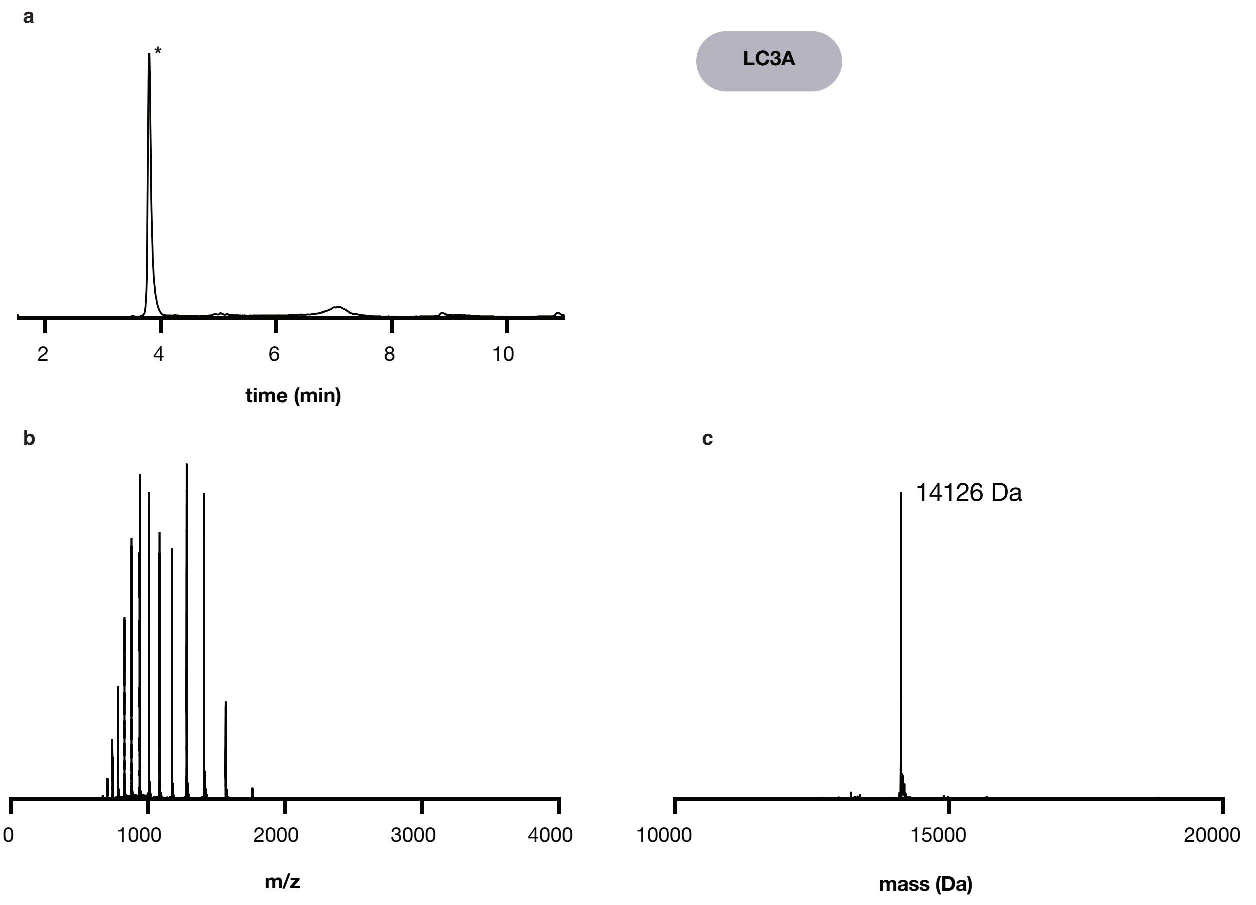

Characterization of LC3A. a LC chromatogram of recombinantly expressed LC3A. TIC is shown. Protein peak is marked with a star. **b** Mass-spectrum (ESI) of recombinantly expressed LC3A. **c** Deconvoluted mass spectrum of LC3A. Expected mass 14125 Da.

4.12. LC3A(ΔG120)–$NHNH_2$

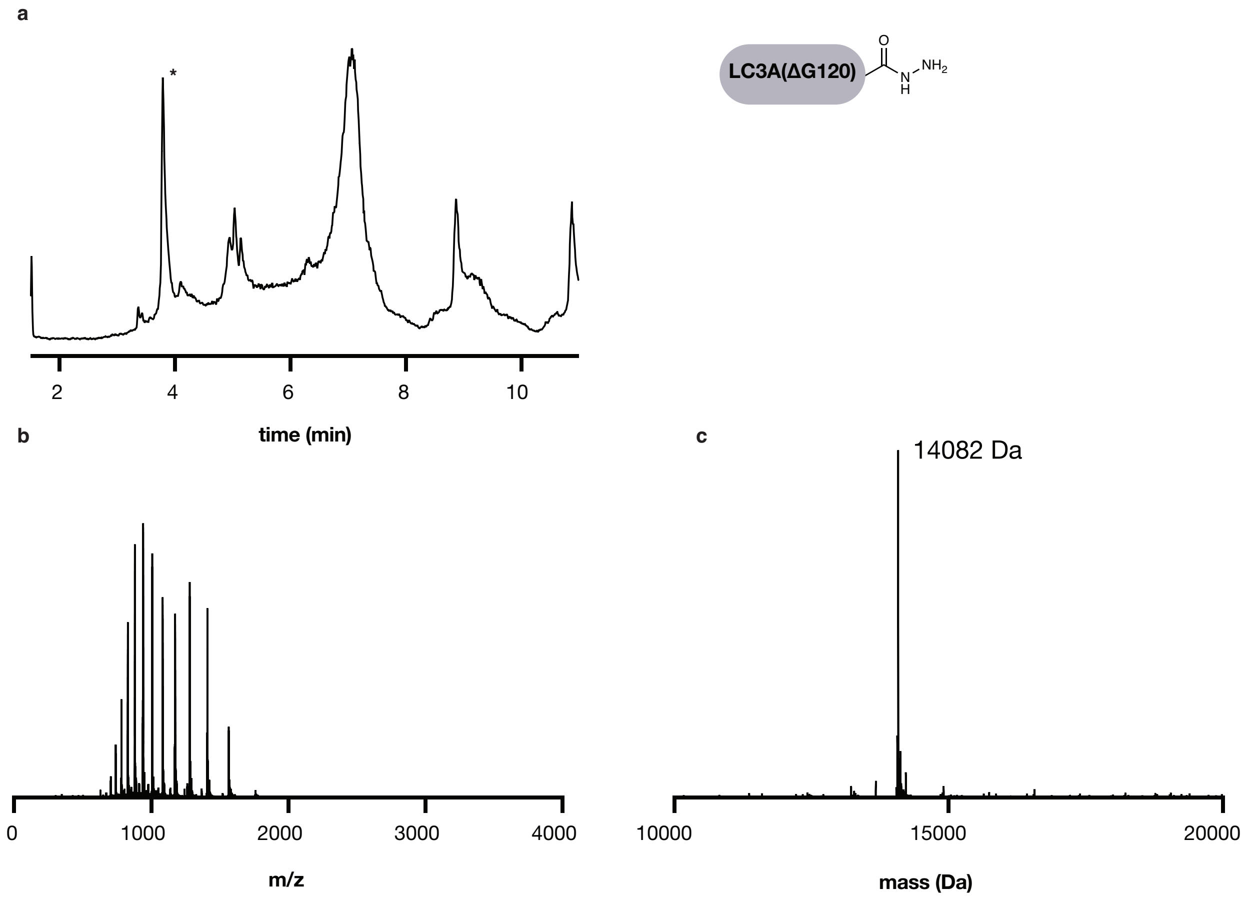

Characterization of LC3A(ΔG120)–$NHNH_2$. a LC-chromatogram of LC3A(ΔG120)–$NHNH_2$. TIC is shown. Protein peak is marked with a star. **b** Mass-spectrum (ESI) of LC3A(ΔG120)–$NHNH_2$. **c** Deconvoluted mass spectrum of LC3A(ΔG120)–$NHNH_2$. Expected mass 14082 Da.

4.13. LC3A(ΔG120)–NHNH α-chloroacetyl 3

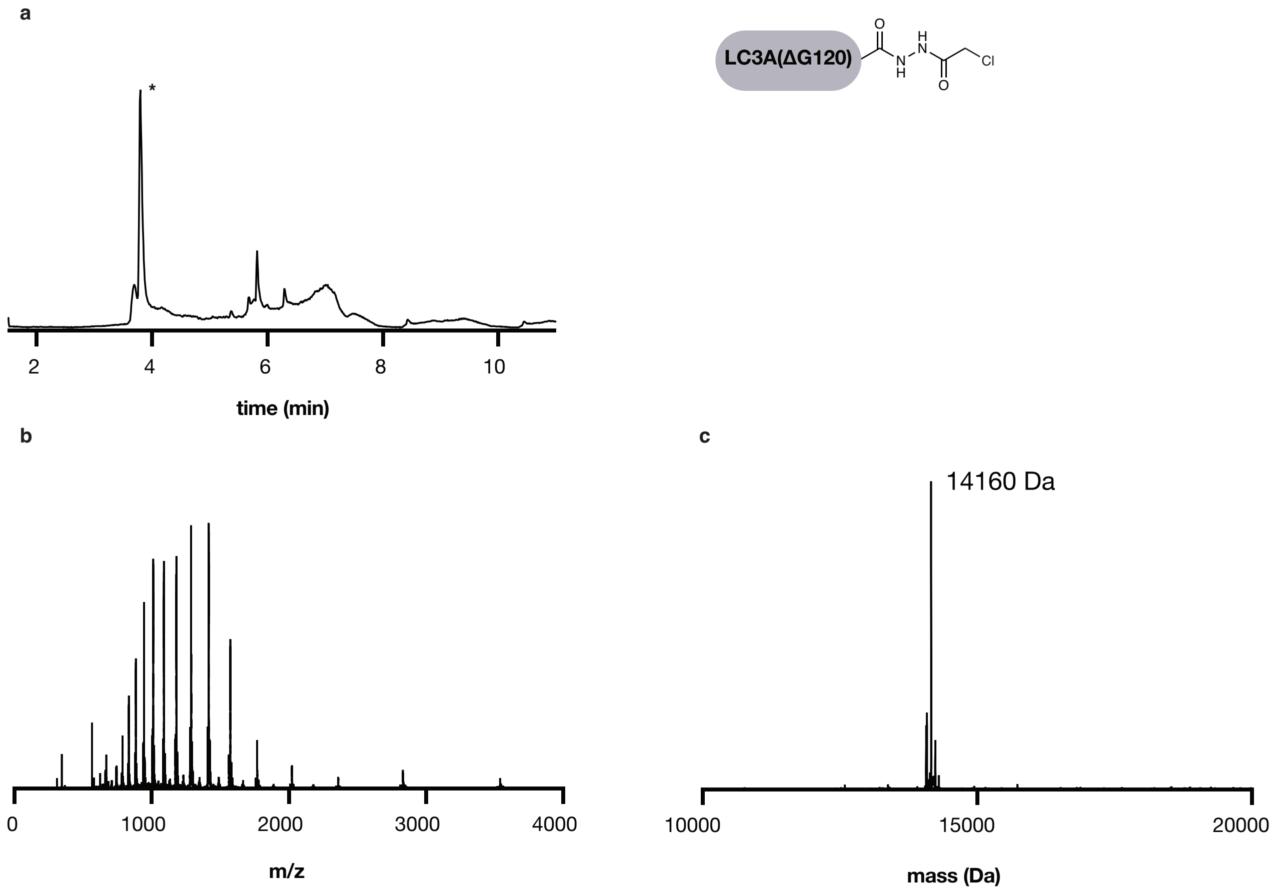

Characterization of LC3A(ΔG120)–NHNH α-chloroacetyl. a LC-chromatogram of LC3A(ΔG120)–NHNH α-chloroacetyl. TIC is shown. Protein peak is marked with a star. **b** Mass-spectrum (ESI) of LC3A(ΔG120)–NHNH α-chloroacetyl. **c** Deconvoluted mass spectrum of LC3A(ΔG120)–NHNH α-chloroacetyl. Expected mass 14158 Da.

4.14. LC3A(ΔG120)–NHNH methyl fumarate 4

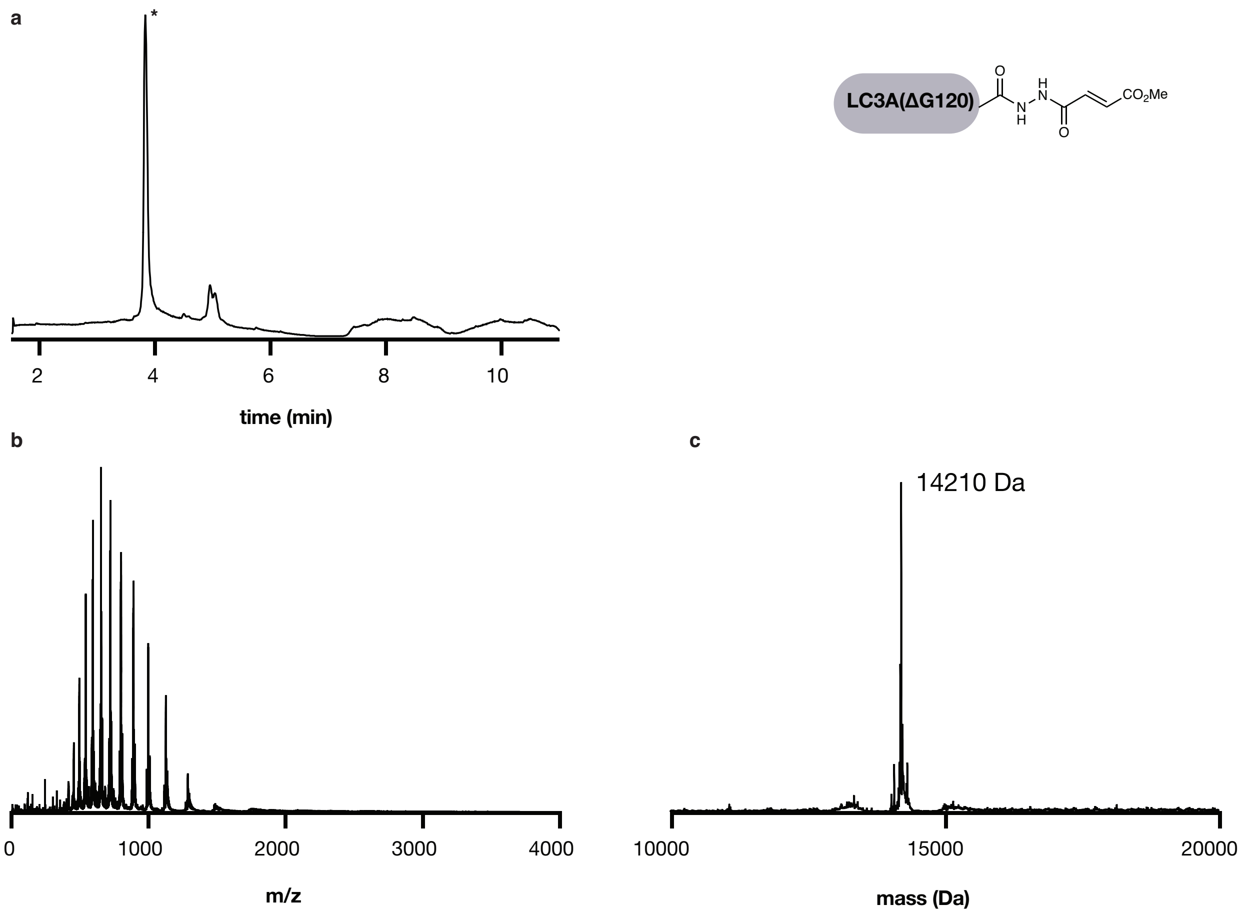

Characterization of LC3A(ΔG120)–NHNH methyl fumarate. **a** LC-chromatogram of LC3A(ΔG120)–NHNH methyl fumarate. TIC is shown. Protein peak is marked with a star. **b** Mass-spectrum (ESI) of LC3A(ΔG120)–NHNH methyl fumarate. **c** Deconvoluted mass spectrum of LC3A(ΔG120)–NHNH methyl fumarate. Expected mass 14210 Da.

4.15. GABARAP fluorescein

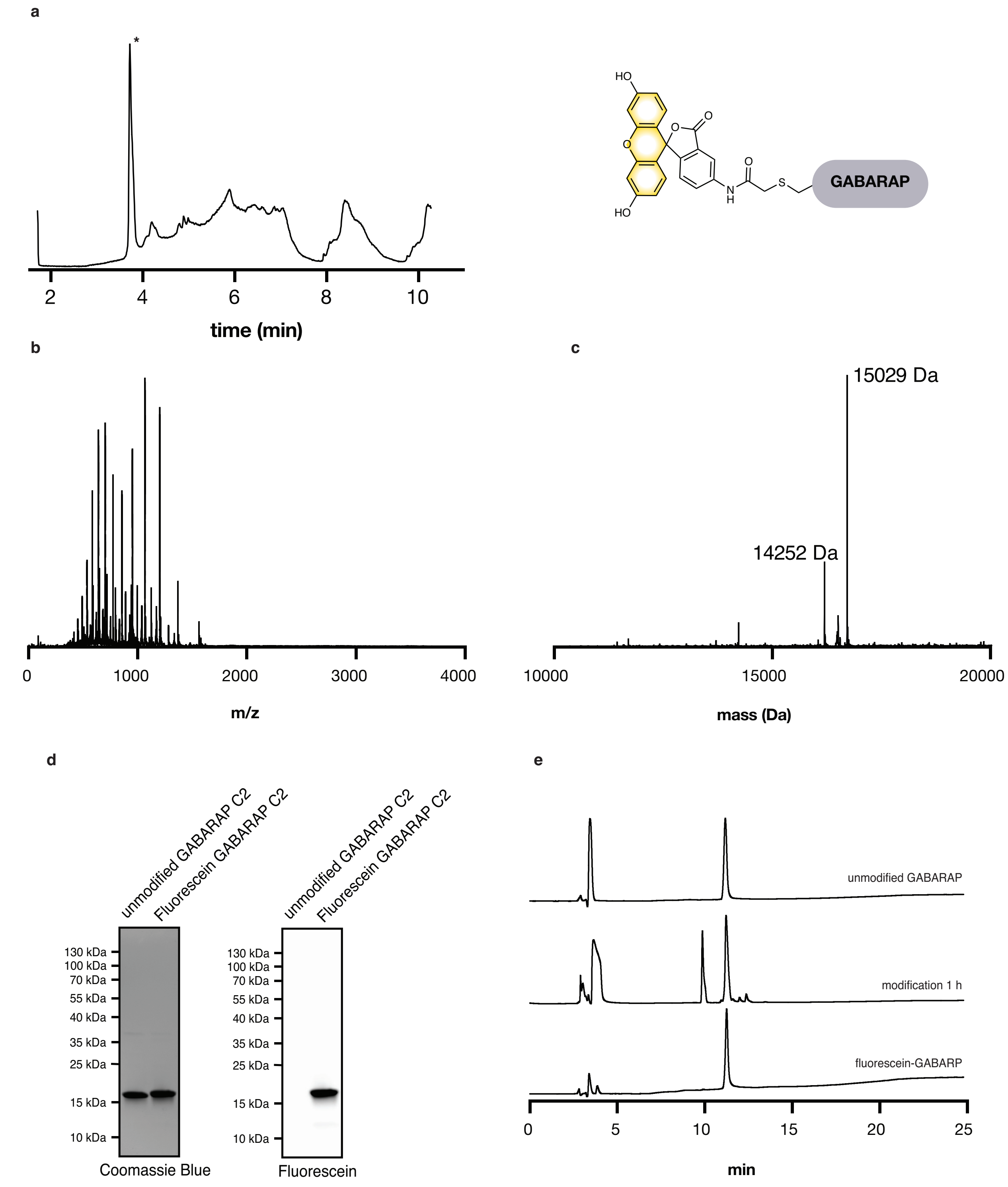

Characterization of fluorescein-GABARAP. **a** LC-chromatogram of fluorescein-GABARAP. TIC is shown. Protein peak is marked with a star. **b** Mass-spectrum (ESI) of fluorescein-GABARAP. **c** Deconvoluted mass spectrum of fluorescein-GABARAP. Expected mass 15029 Da. **d** SDS-PAGE gel analysis of GABARAP before and after modification with 5-Iodoacetamide fluorescein. Analysis by Coomassie Blue staining (left gel) and in-gel fluorescence (right gel). **E** RP-HPLC chromatograms showing unmodified GABARAP, crude chromatogram of GABARAP modification with 5-Iodoacetamide fluorescein, and purified fluorescein-GABARAP.

5. Amino acid sequences of recombinantly expressed proteins

Protein	Amino acid sequence	Comments
Ubiquitin (ΔGG)-**GyrA-His$_6$**	MQIFVKTLTGKTITLEVEPSDTIENVKAKIQDKEGIPPDQQRLIFAGKQL<br>EDGRTLSDYNIQKESTLHLVLRLR**CITGDALVALPEGESVRIADIVPGAR**<br>**PNSDNAIDLKVLDRHGNPVLADRLFHSGEHPVYTVRTVEGLRVTGTANHP**<br>**LLCLVDVAGVPTLLWKLIDEIKPGDYAVIQRSAFSVDCAGFARGKPEFAP**<br>**TTYTVGVPGLVRFLEAHHRDPDAQAIADELTDGRFYYAKVASVTDAGVQP**<br>**VYSLRVDTADHAFITNGFVSHALEHHHHHH**	*Mxe*-GyrA intein with C-terminal His$_6$
SUMO-GABARAP (ΔG116)-**GyrA-His$_6$**	**MGSDSEVNQEAKPEVKPEVKPETHINLKVSDGSSEIFFKIKKTTPLRRLM**<br>**EAFAKRQGKEMDSLRFLYDGIRIQADQTPEDLDMEDNDIIEAHREQIGG**K<br>FVYKEEHPFEKRRSEGEKIRKKYPDRVPVIVEKAPKARIGDLDKKKYLVP<br>SDLTVGQFYFLIRKRIHLRAEDALFFFVNNVIPPTSATMGQLYQEHHEED<br>FFLYIAYSDESVY**CITGDALVALPEGESVRIADIVPGARPNSDNAIDLKV**<br>**LDRHGNPVLADRLFHSGEHPVYTVRTVEGLRVTGTANHPLLCLVDVAGVP**<br>**TLLWKLIDEIKPGDYAVIQRSAFSVDCAGFARGKPEFAPTTYTVGVPGLV**<br>**RFLEAHHRDPDAQAIADELTDGRFYYAKVASVTDAGVQPVYSLRVDTADH**<br>**AFITNGFVSHALEHHHHHH**	*Mxe*-GyrA intein with C-terminal His$_6$
**His$_6$-SUMO**-GABARAP	**MGSSHHHHHHGSDSEVNQEAKPEVKPEVKPETHINLKVSDGSSEIFFKIK**<br>**KTTPLRRLMEAFAKRQGKEMDSLRFLYDGIRIQADQTPEDLDMEDNDIIE**<br>**AHREQIGG**KFVYKEEHPFEKRRSEGEKIRKKYPDRVPVIVEKAPKARIGD<br>LDKKKYLVPSDLTVGQFYFLIRKRIHLRAEDALFFFVNNVIPPTSATMGQ<br>LYQEHHEEDFFLYIAYSDESVYG	N-terminal His$_6$ with SUMO-tag
SUMO-LC3A(ΔG120) **-GyrA-His$_6$**	**MGSDSEVNQEAKPEVKPEVKPETHINLKVSDGSSEIFFKIKKTTPLRRLM**<br>**EAFAKRQGKEMDSLRFLYDGIRIQADQTPEDLDMEDNDIIEAHREQIGG**M<br>PSDRPFKQRRSFADRCKEVQQIRDQHPSKIPVIIERYKGEKQLPVLDKTK<br>FLVPDHVNMSELVKIIRRRLQLNPTQAFFLLVNQHSMVSVSTPIADIYEQ<br>EKDEDGFLYMVYASQETF**CITGDALVALPEGESVRIADIVPGARPNSDNA**<br>**IDLKVLDRHGNPVLADRLFHSGEHPVYTVRTVEGLRVTGTANHPLLCLVD**<br>**VAGVPTLLWKLIDEIKPGDYAVIQRSAFSVDCAGFARGKPEFAPTTYTVG**<br>**VPGLVRFLEAHHRDPDAQAIADELTDGRFYYAKVASVTDAGVQPVYSLRV**<br>**DTADHAFITNGFVSHALEHHHHHH**	*Mxe*-GyrA intein with C-terminal His$_6$
**His$_6$-SUMO-**ATG3 wt	**MGSSHHHHHHGSDSEVNQEAKPEVKPEVKPETHINLKVSDGSSEIFFKIK**<br>**KTTPLRRLMEAFAKRQGKEMDSLRFLYDGIRIQADQTPEDLDMEDNDIIE**<br>**AHREQIGG**QNVINTVKGKALEVAEYLTPVLKESKFKETGVITPEEFVAAG<br>DHLVHHCPTWQWATGEELKVKAYLPTGKQFLVTKNVPCYKRCKQMEYSDE<br>LEAIIEEDDGDGGWVDTYHNTGITGITEAVKEITLENKDNIRLQDCSALC<br>EEEEDEDEGEAADMEEYEESGLLETDEATLDTRKIVEACKAKTDAGGEDA<br>ILQTRTYDLYITYDKYYQTPRLWLFGYDEQRQPLTVEHMYEDISQDHVKK<br>TVTIENHPHLPPPPMCSVHPCRHAEVMKKIIETVAEGGGELGVHMYLLIF<br>LKFVQAVIPTIEYDYTRHFTM	N-terminal His$_6$ with SUMO-tag
**His$_6$-SUMO-**ATG3 ΔLIR	**MGSSHHHHHHGSDSEVNQEAKPEVKPEVKPETHINLKVSDGSSEIFFKIK**<br>**KTTPLRRLMEAFAKRQGKEMDSLRFLYDGIRIQADQTPEDLDMEDNDIIE**<br>**AHREQIGG**QNVINTVKGKALEVAEYLTPVLKESKFKETGVITPEEFVAAG<br>DHLVHHCPTWQWATGEELKVKAYLPTGKQFLVTKNVPCYKRCKQMEYSDE<br>LRGHNTGITGITEAVKEITLENKDNIRLQDCSALCEEEEDEDEGEAADME<br>EYEESGLLETDEATLDTRKIVEACKAKTDAGGEDAILQTRTYDLYITYDK<br>YYQTPRLWLFGYDEQRQPLTVEHMYEDISQDHVKKTVTIENHPHLPPPPM<br>CSVHPCRHAEVMKKIIETVAEGGGELGVHMYLLIFLKFVQAVIPTIEYDY<br>TRHFTM	N-terminal His$_6$ with SUMO-tag
**His$_6$-SUMO-**ATG3 C1	**MGSSHHHHHHGSDSEVNQEAKPEVKPEVKPETHINLKVSDGSSEIFFKIK**<br>**KTTPLRRLMEAFAKRQGKEMDSLRFLYDGIRIQADQTPEDLDMEDNDIIE**<br>**AHREQIGG**NVINTVKGKALEVAEYLTPVLKESKFKETGVITPEEFVAAGD<br>HLVHHAPTWQWATGEELKVKAYLPTGKQFLVTKNVPAYKRAKQMEYSDEL<br>EAIIEEDDGDGGWVDTYHNTGITGITEAVKEITLENKDNIRLQDASALAE<br>EEEDEDEGEAADMEEYEESGLLETDEATLDTRKIVEAAKAKTDAGGEDAI<br>LQTRTYDLYITYDKYYQTPRLWLFGYDEQRQPLTVEHMYEDISQDHVKKT	N-terminal His$_6$ with SUMO-tag

	VTIENHPHLPPPPMASVHPCRHAEVMKKIIETVAEGGGELGVHMYLLIFL KFVQAVIPTIEYDYTRHFTM	
**His$_6$-SUMO-** ATG3 C1 ΔLIR	**MGSSHHHHHHGSDSEVNQEAKPEVKPEVKPETHINLKVSDGSSEIFFKIK KTTPLRRLMEAFAKRQGKEMDSLRFLYDGIRIQADQTPEDLDMEDNDIIE AHREQIGG**NVINTVKGKALEVAEYLTPVLKESKFKETGVITPEEFVAAGD HLVHHAPTWQWATGEELKVKAYLPTGKQFLVTKNVPAYKRAKQMEYSDEL RGHNTGITGITEAVKEITLENKDNIRLQDASALAEEEEDEDEGEAADMEE YEESGLLETDEATLDTRKIVEAAKAKTDAGGEDAILQTRTYDLYITYDKY YQTPRLWLFGYDEQRQPLTVEHMYEDISQDHVKKTVTIENHPHLPPPPMA SVHPCRHAEVMKKIIETVAEGGGELGVHMYLLIFLKFVQAVIPTIEYDYT RHFTM	N-terminal His$_6$ with SUMO-tag
**His$_6$-SUMO-** LC3A	**MGSSHHHHHHGSDSEVNQEAKPEVKPEVKPETHINLKVSDGSSEIFFKIK KTTPLRRLMEAFAKRQGKEMDSLRFLYDGIRIQADQTPEDLDMEDNDIIE AHREQIGG**MPSDRPFKQRRSFADRCKEVQQIRDQHPSKIPVIIERYKGEK QLPVLDKTKFLVPDHVNMSELVKIIRRRLQLNPTQAFFLLVNQHSMVSVS TPIADIYEQEKDEDGFLYMVYASQETFG	N-terminal His$_6$ with SUMO-tag
His-TEV-Ube2K (C170S)	MGSSHHHHHHSSGAENLYFQGMANIAVQRIKREFKEVLKSEETSKNQIKV DLVDENFTELRGEIAGPPDTPYEGGRYQLEIKIPETYPFNPPKVRFITKI WHPNISSVTGAICLDILKDQWAAAMTLRTVLLSLQALLAAAEPDDPQDAV VANQYKQNPEMFKQTARLWAHVYAGAPVSSPEYTKKIENLSAMGFDRNAV IVALSSKSWDVETATELLLSN	N-terminal His$_6$ with TEV cleavage site
His-thrombin-SENP1 (419-644)	MGSSHHHHHHSSGLVPRGSHMEFPEITEEMEKEIKNVFRNGNQDEVLSEA FRLTITRKDIQTLNHLNWLNDEIINFYMNMLMERSKEKGLPSVHAFNTFF FTKLKTAGYQAVKRWTKKVDVFSVDILLVPIHLGVHWCLAVVDFRKKNIT YYDSMGGINNEACRILLQYLKQESIDKKRKEFDTNGWQLFSKKSQEIPQQ MNGSDAGMFACKYADCITKDRPINFTQQHMPYFRKRMVWEILHRKLL	N-terminal His$_6$ with thrombin cleavage site

Table S4 Amino acid sequences of recombinantly expressed proteins used in this study. Purification handles cleaved after expression are shown in bold.

6. Primer sequences

Protein	Mutation	Primer	Sequence
**His$_6$-SUMO-** ATG3	C264A	Forward primer (5'–3')	AGTTCACCCAGCGAGGCATGCTGAGG
		Reverse primer (5'–3')	GAACACATGGGAGGTGGT
**His$_6$-SUMO-** ATG3	LIR deletion	Forward primer (5'–3')	GGCCACAACACAGGTATTACAGGAA
		Reverse primer (5'–3')	CCGCAATTCATCTGAATATTCCATCTGTTT
**His$_6$-SUMO-** ATG3	Y90A	Forward primer (5'–3')	GCGTCAGATGAATTGGAAGCTATCATTGAAGAAGA
		Reverse primer (5'–3')	TTCCATCTGTTTCGCCCGC
**His$_6$-SUMO-** ATG3	D92A	Forward primer (5'–3')	GCGGAATTGGAAGCTATCATTGAAGAAGATGATGG
		Reverse primer (5'–3')	TGAATATTCCATCTGTTTCGCCCG
**His$_6$-SUMO-** ATG3	E95A	Forward primer (5'–3')	AGATGAATTGGCGGCTATCATTGAAG
		Reverse primer (5'–3')	GAATATTCCATCTGTTTCG
**His$_6$-SUMO-** ATG3	I97A	Forward primer (5'–3')	ATTGGAAGCTGCGATTGAAGAAGATGATG
		Reverse primer (5'–3')	TCATCTGAATATTCCATCTG
	I98A	Forward primer (5'–3')	GGAAGCTATCGCGGAAGAAGATGATGG

**His$_6$-SUMO-**ATG3		Reverse primer (5'–3')	AATTCATCTGAATATTCCATCTG
**His$_6$-SUMO-**ATG3	E99A	Forward primer (5'–3')	GCGGAAGATGATGGTGATGGCGGATG
		Reverse primer (5'–3')	AATGATAGCTTCCAATTCATCTGAATATTCCATC
**His$_6$-SUMO-**ATG3	D102A	Forward primer (5'–3')	GCGGGTGATGGCGGATGGGTAGA
		Reverse primer (5'–3')	ATCTTCTTCAATGATAGCTTCCAATTCATCTG
**His$_6$-SUMO-**ATG3	D104A	Forward primer (5'–3')	GCGGGCGGATGGGTAGATACATATCACA
		Reverse primer (5'–3')	ACCATCATCTTCTTCAATGATAGCTTCCA
**His$_6$-SUMO-**ATG3	W107A	Forward primer (5'–3')	GCGGTAGATACATATCACAACACAGGTATTACAGGA
		Reverse primer (5'–3')	TCCGCCATCACCATCATCTTCT
**His$_6$-SUMO-**ATG3	V108A	Forward primer (5'–3')	GCGGATACATATCACAACACAGGTATTACAGGAA
		Reverse primer (5'–3')	CCATCCGCCATCACCATCATC
**His$_6$-SUMO-**ATG3	D109A	Forward primer (5'–3')	GCGACATATCACAACACAGGTATTACAGGAATAACG
		Reverse primer (5'–3')	TACCCATCCGCCATCACCATCA
**His$_6$-SUMO-**ATG3	T110A	Forward primer (5'–3')	GCGTATCACAACACAGGTATTACAGGAATAACGGA
		Reverse primer (5'–3')	ATCTACCCATCCGCCATCACC
**His$_6$-SUMO-**ATG3	Y111A	Forward primer (5'–3')	GCGCACAACACAGGTATTACAGGAATAACGG
		Reverse primer (5'–3')	TGTATCTACCCATCCGCCATCAC

Table S5 Primer sequences used to introduce deletions and point mutations for proteins used in this study.

experiment	primer ID	primer	sequence
sgRNA production	sgATG3.KD1_seq	Forward primer (5'–3')	TTGGGACTCCCTGGCCCCTGACAGTTTAAGAGC
sgRNA production		Reverse primer (5'–3')	TTAGCTCTTAAACTGTCAGGGGCCAGGGAGTCCCAACAAG
sgRNA production	sgATG3.KD2_seq	Forward primer (5'–3')	TTGGTCACGTGAGGCCCCGGTGGGTTTAAGAGC
sgRNA production		Reverse primer (5'–3')	TTAGCTCTTAAACCCACCGGGGCCTCACGTGACCAACAAG
sgRNA production	sgATG3.KD3_seq	Forward primer (5'–3')	TTGGCCCGGCTGGCAGCACCCGAGTTTAAGAGC
sgRNA production		Reverse primer (5'–3')	TTAGCTCTTAAACTCGGGTGCTGCCAGCCGGGCCAACAAG
sgRNA production	sgATG3.KD4_seq	Forward primer (5'–3')	TTGGGAAAGTGCAGCCGTGTCAGGTTTAAGAGC
sgRNA production		Reverse primer (5'–3')	TTAGCTCTTAAACCTGACACGGCTGCACTTTCCCAACAAG

Table S6 Primer sequences used to generate sgRNAs for ATG3 knock-down. Protospacer sequences are underlined.

experiment	primer ID	primer	sequence
sgRNA production	T7FwdVar-sgATG3.KO3	extension PCR template (5'-3')	GGATCCTAATACGACTCACTATAGGTGAACTGAACACATGGGGTTTTAGAGCTAGAA
sgRNA production	T7FwdVar-sgATG3.KO4	extension PCR template (5'-3')	GGATCCTAATACGACTCACTATAGTGGCTGAGTACCTGACCCGTTTTAGAGCTAGAA
sgRNA production	T7RevLong	extension PCR template (5'-3')	AAAAAAGCACCGACTCGGTGCCACTTTTTCAAGTTGATAACGGACTAGCCTTATTTTAACTTGCTATTTCTAGCTCTAAAAC
sgRNA production	T7FwdAmp	Forward primer (5'–3')	GGATCCTAATACGACTCACTATAG
sgRNA production	T7RevAmp	Reverse primer (5'–3')	AAAAAAGCACCGACTCGG
clone genotyping	sgATG3.KO3_seq	Forward primer (5'–3')	CTTTCAGCAACGGCAGCCTT
clone genotyping		Reverse primer (5'–3')	AGCACAAGAAAATTTCTAAGGAGGAC
clone genotyping	sgATG3.KO4_seq	Forward primer (5'–3')	CACGGCTGCACTTTCCATCC
clone genotyping		Reverse primer (5'–3')	CTCGAGCCCTACTGCCTTCC

Table S7 Primer sequences used to generate sgRNAs for ATG3 knockout and for PCR amplification of edited loci. Protospacer sequences are underlined.

7. Uncropped gels and blots

Figure 1b, Coomassie Blue

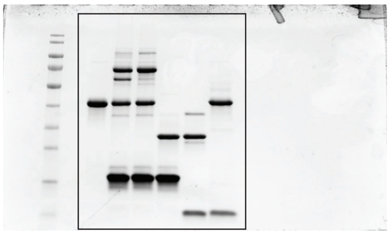

Figure 1d, Coomassie Blue

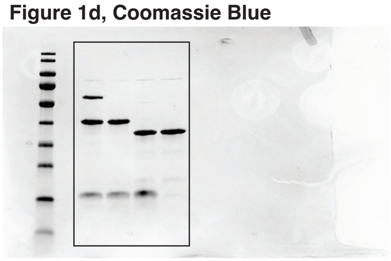

Figure 2b, Coomassie Blue Replicate 1

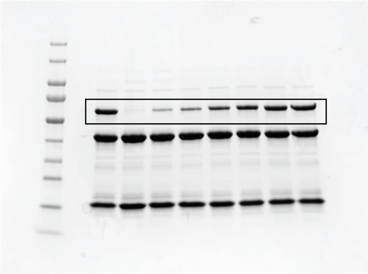

Figure 2b, Coomassie Blue Replicate 2

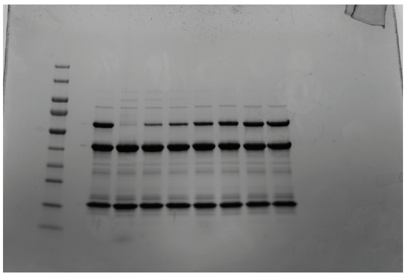

Figure 2b, Coomassie Blue Replicate 2

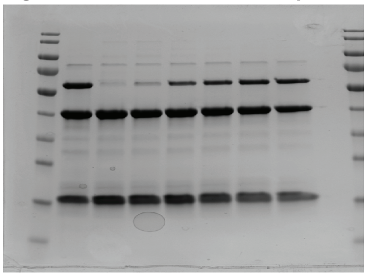

Figure 2c, Coomassie Blue Replicate 1

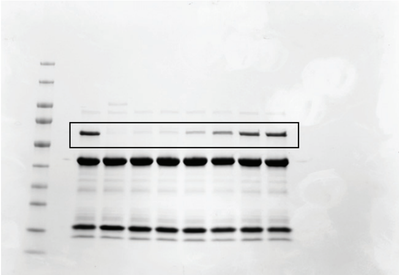

Figure 2c, Coomassie Blue Replicate 2

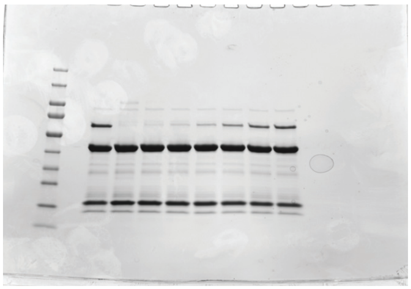

Figure 2c, Coomassie Blue Replicate 3

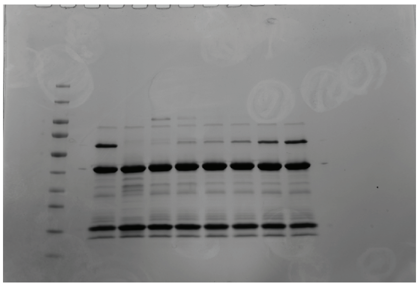

Figure 4, Replicate 1

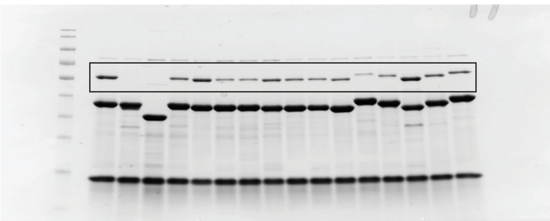

Figure 4, Coomassie Blue, Replicate 2

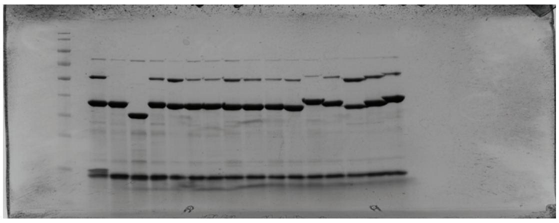

Figure 5d, Fluorescein, Replicate 1

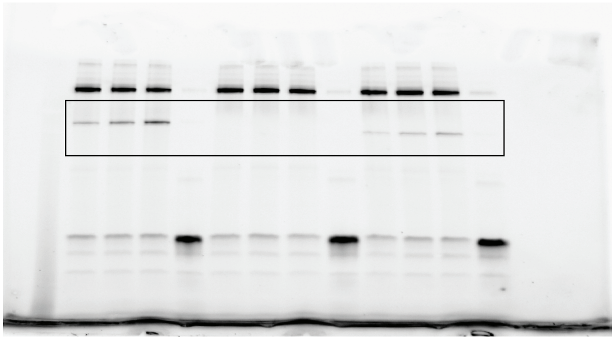

Figure 5d, Stain-Free, Replicate 1

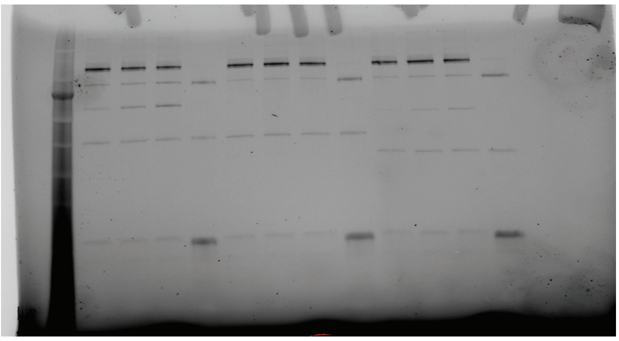

Figure 5d, Fluorescein, Replicate 2

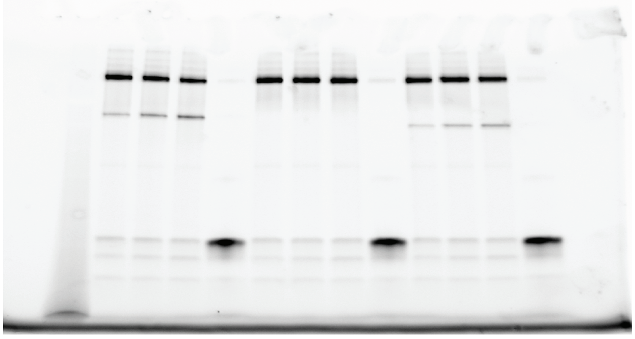

Figure 5d, Stain-Free, Replicate 2

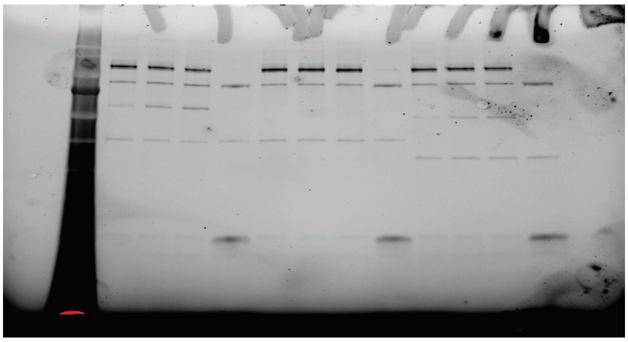

Figure 5d, Fluorescein, Replicate 2

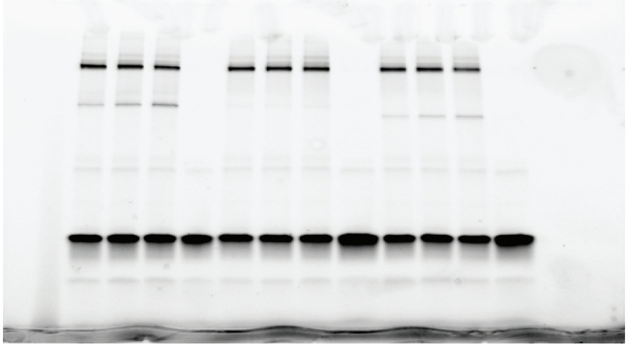

Figure 5d, Stain-Free, Replicate 2

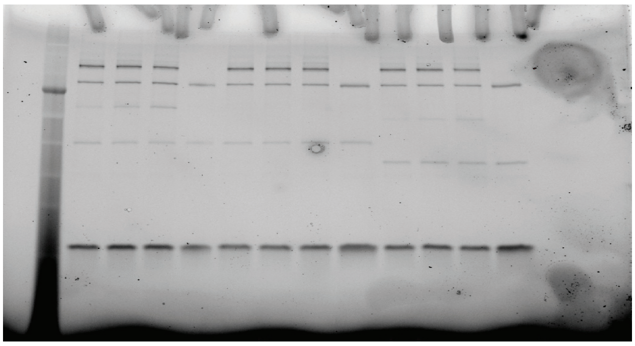

Figure 5a, LC3A

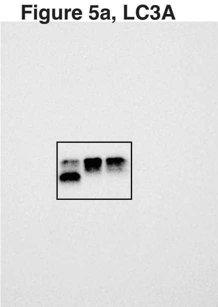

Figure 5a, ATG3

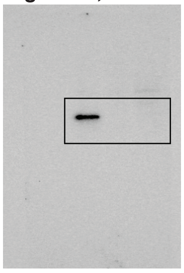

Figure 5a, beta-Actin

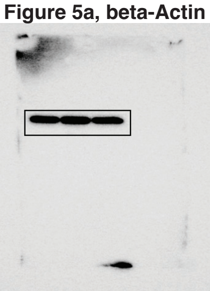

Figure 5b, Figure S15

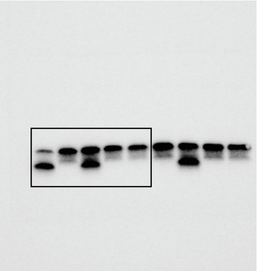

Figure 5b, Figure S15

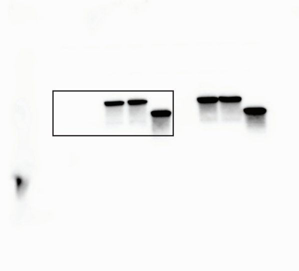

Figure 5b, Figure S15

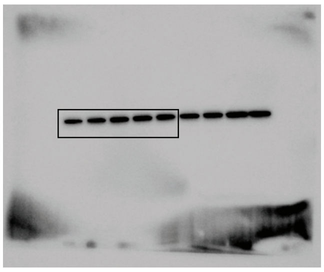

Figure 5c

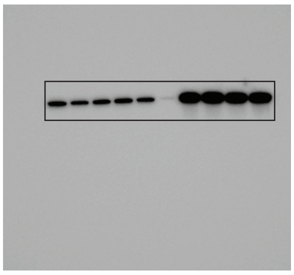

Figure 5c

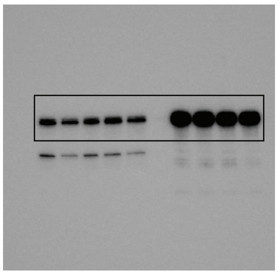

Figure 5c

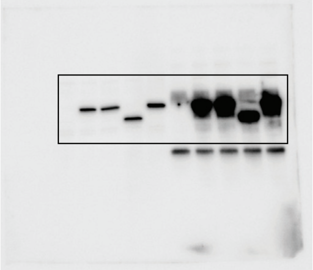

Figure 5c

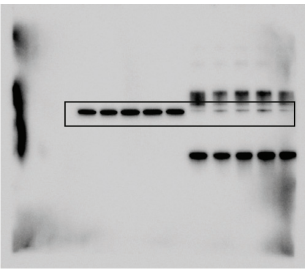

Figure 5g

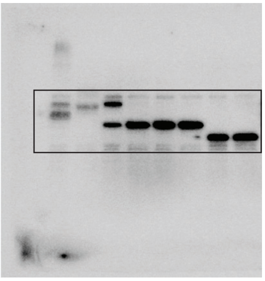

Figure 5g

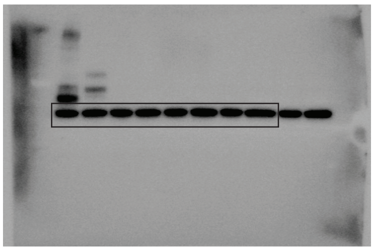

Figure S9, Coomassie Blue

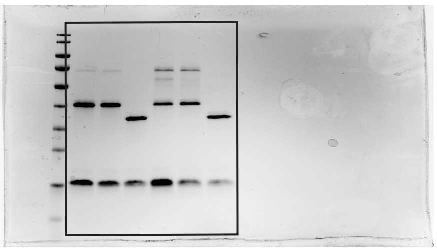

Figure S10, Coomassie Blue

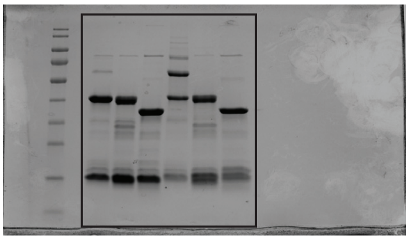

Figure S13c, Coomassie Blue, Replicate 1

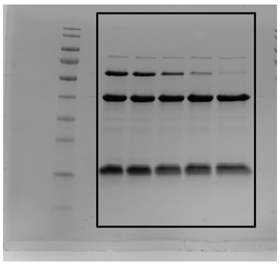

Figure S13c, Coomassie Blue, Replicate 2

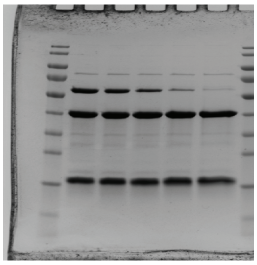

Figure S13c, Coomassie Blue, Replicate 3

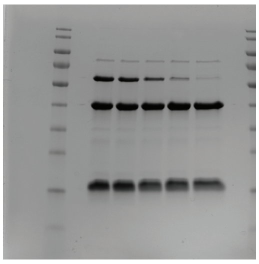

Figure S16, Sypro Ruby

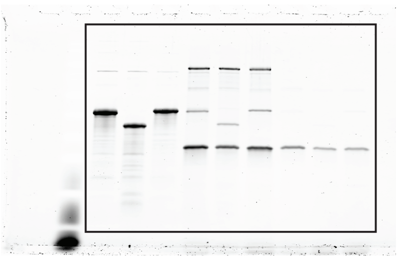

Figure S2, Coomassie Blue

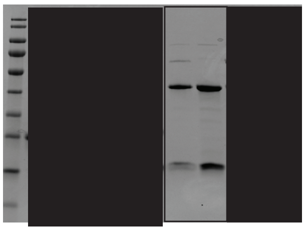

8. References

(1) Raasi, S.; Pickart, C. M. Ubiquitin chain synthesis. *Methods Mol Biol* **2005**, *301*, 47-55.

(2) Kabsch, W. Xds. *Acta Crystallogr D Biol Crystallogr* **2010**, *66* (Pt 2), 125-132.

(3) Huber, J.; Obata, M.; Gruber, J.; Akutsu, M.; Lohr, F.; Rogova, N.; Guntert, P.; Dikic, I.; Kirkin, V.; Komatsu, M.; et al. An atypical LIR motif within UBA5 (ubiquitin like modifier activating enzyme 5) interacts with GABARAP proteins and mediates membrane localization of UBA5. *Autophagy* **2020**, *16* (2), 256-270.

(4) McCoy, A. J.; Grosse-Kunstleve, R. W.; Adams, P. D.; Winn, M. D.; Storoni, L. C.; Read, R. J. Phaser crystallographic software. *J Appl Crystallogr* **2007**, *40* (Pt 4), 658-674.

(5) Afonine, P. V.; Grosse-Kunstleve, R. W.; Echols, N.; Headd, J. J.; Moriarty, N. W.; Mustyakimov, M.; Terwilliger, T. C.; Urzhumtsev, A.; Zwart, P. H.; Adams, P. D. Towards automated crystallographic structure refinement with phenix.refine. *Acta Crystallogr D Biol Crystallogr* **2012**, *68* (Pt 4), 352-367.

(6) Emsley, P.; Cowtan, K. Coot: model-building tools for molecular graphics. *Acta Crystallogr D* **2004**, *60*, 2126-2132.

(7) Pettersen, E. F.; Goddard, T. D.; Huang, C. R. C.; Meng, E. E. C.; Couch, G. S.; Croll, T. I.; Morris, J. H.; Ferrin, T. E. UCSF ChimeraX: Structure visualization for researchers, educators, and developers. *Protein Sci* **2021**, *30* (1), 70-82.

(8) Larson, M. H.; Gilbert, L. A.; Wang, X. W.; Lim, W. A.; Weissman, J. S.; Qi, L. S. CRISPR interference (CRISPRi) for sequence-specific control of gene expression. *Nat Protoc* **2013**, *8* (11), 2180-2196.

(9) Lingeman, E.; Jeans, C.; Corn, J. E. Production of Purified CasRNPs for Efficacious Genome Editing. *Curr Protoc Mol Biol* **2017**, *120*, 31 10 31-31 10 19.

(10) Michlits, G.; Jude, J.; Hinterndorfer, M.; de Almeida, M.; Vainorius, G.; Hubmann, M.; Neumann, T.; Schleiffer, A.; Burkard, T. R.; Fellner, M.; et al. Multilayered VBC score predicts sgRNAs that efficiently generate loss-of-function alleles. *Nat Methods* **2020**, *17* (7), 708-716.
