# Supplementary material for: Semisynthetic LC3 Probes for Autophagy Pathways Reveal a Noncanonical LC3 Interacting Region Motif Crucial for the Enzymatic Activity of Human ATG3
Source: ACS Cent Sci. 2023 Apr 27;9(5):1025–34. doi: 10.1021/acscentsci.3c00009 (PMC10214526; doi:10.1021/acscentsci.3c00009)
Supplement: Supplementary file 2 — oc3c00009_si_002.pdf [file oc3c00009_si_002.pdf]

oc-2023-00009a.R1

Name: Peer Review Information for "Semi-synthetic LC3 probes for autophagy pathways reveal a non-canonical LIR motif crucial for the enzymatic activity of human ATG3"

First Round of Reviewer Comments

Reviewer: 1

Comments to the Author

This is a superbly written manuscript with highly appropriate and detailed experiments. The experiments are all carefully controlled, and well-analyzed and interpreted except in a small number of areas. The ultimate conclusion, that human ATG3 has a noncanonical LIR motif that affects the activity of the ATG3 complex for lipidating LC3/GABARAP, is well-supported.

Enthusiasm for this manuscript is diminished by three things. First, there are issues with the binding affinity (including issues related to data analysis) that should be addressed. Second, some of the finer conclusions are not well-supported, as described below. Finally, while the new LIR motif certainly plays a role, the lack of a convincing model for how it affects function of the ATG7-ATG3 complex detracts from the long-term impact of the work. More details on each of these three points are below. Ultimately, even with the first set of issues addressed, I do not see this work being of broad interest to researchers outside the fields of autophagy and protein-protein interactions. Thus, I suggest fixing the methodological issues (point 1 below), perhaps adding a second binding assay to improve confidence in the Kd values, and then publishing in a more specialized journal such as ACS Chemical Biology.

1. The binding affinities for peptides that included the novel LIR motif were measured by fluorescence polarization. However, the data in figures 2e,f have some issues. Overall, it would be good to display these data on a log-scale x-axis so data at lower concentrations can be seen more clearly. Also, the data points in Figure 2e and 2f do not appear to line up with the values stated in the methods section (this is most obvious in Figure 2e, but appears to be the case for 2f as well). Most importantly, the curve fits do not look like correct curve fits to extract Kd values from fraction-bound data. Specifically, Kd curves fit to the appropriate equation (for example, equation 5 in Dan Herschlag's excellent 2020 eLife resource https://elifesciences.org/articles/57264) are monotonic and do not decrease after saturating at high concentrations. However, some of these curves appear to decrease after saturating at high concentrations. These data should be double-checked and re-analyzed given these concerns.

Given these issues and the importance of measuring the affinity accurately, perhaps there is another assay (such as SPR, BLI, or AlphaLISA) that the authors could use to measure binding affinity to correlate their Kd values.

2. The authors state that "The stronger affinity of the cyclic peptides supports the importance of the β-sheet conformation in binding to LC3A and GABARAP". However, as their CD data show (Supplementary Figure 12), the cyclic peptide does not have a stable beta-sheet conformation in aqueous solution. It

may be that there is some degree of pre-organization within the cyclic peptide, but there is no evidence for it in the manuscript. Later, the authors also write “We provide conclusive evidence that the bent β-sheet conformation for LIRATG3 is required for efficient binding to LC3/GABARAP and is not an artifact of co-crystallizing LIR peptide and LC3/GABARAP”, but if this evidence is limited to the binding data with linear and cyclic peptides, then this evidence is not even close to conclusive. These interpretations are complicated by the uncertainty surrounding the binding affinity data, since a peptide-protein complex with high micromolar affinity may crystallize (especially because GABARAP itself crystallizes relatively easily) but may have more flexible regions and nonspecific interactions that are prone to crystallization artifacts. If the affinity is indeed sub-micromolar, this may strengthen the case that the beta-sheet observed in the crystal structure is important for function. Even then, however, I don’t think these data would comprise “conclusive evidence that the bent β-sheet conformation for LIRATG3 is required for efficient binding to LC3/GABARAP”.

These interpretations should be omitted or additional evidence showing the importance of a beta-sheet conformation (rather than just an extended linear conformation) should be provided.

On a related point, the alanine scanning data indicate that W107 and V108 are important for ATG3-GABARAP cross-linking (and, by inference, ATG3-GABARAP binding). This is consistent with the crystal structure. However, the alanine scanning data also indicate that D102, I97, and E95 are at least as important as V108, but the specific importance of these residues do not appear to be rationalized by the crystal structure. This provides additional uncertainty that the crystal structure may not be showing the complete set of interactions required for the observed binding, and may imply that the actual binding motif involves a different conformation, or multiple different conformations.

3. The cellular work includes diligently performed ATG3 knockout experiments and complementation experiments with ATG3 variants. The results of these experiments, and the results of work with GABARAP-derived probe 2, suggest that the delta-LIR construct of ATG3 is impaired in its ability to mediate LC3/GABARAP lipidation. However, the results with a pulse-chase assay looking at ATG3-GABARAP complex formation shows impairment, but not abolishment, of the functional interaction. The authors do a good job trying to explain these nuances in the discussion. However, the model shown in figure 5f and the discussion of possible mechanisms are highly speculative. Ultimately, while the data do support a role for this noncanonical LIR motif, a clean model for how this portion of the flexible region of ATG3 affects activity of the lipidation complex remains elusive.

Reviewer: 2

Comments to the Author

Summary of this manuscript

J. Farnung et al. prepared GABARAP/LC3 ABPs using the hydrazide acylation protocol developed by the Bode group. These ABPs enabled the identification of covalently linked GABARAP/LCL3-ATG3 complexes. The formation of a crosslinking complex is more reliable evidence compared to the results of the binding assay (Sci. Adv. 2021, 7, 40). The structural prediction by ColabFold has suggested a non-canonical β-sheet LIR motif of ATG3, and a co-crystal structure of the GABARAP-LIR peptide complex clearly showed the new LIR motif. Most researchers had missed an important motif consisting of 'WXXT' sequences for

many years before this study. Since the scope of LIR motifs has been expanded, the discovery of new LIR and proteins involved in autophagy is expected. The combination of GABARAP/LC3 ABPs and ColabFold prediction enabled them for this novel discovery. Furthermore, the importance of this non-canonical LIR motif in LC3 lipidation is confirmed in cellulo experiments. These experimental results support the novelty of the discovery.

Major points of critiques

(1) Salt-dependent cross-linking efficiency was discussed (page 13, line 32, Supplementary Figure 13), but the crosslinking needed two steps, LIR-GABARAP interaction and the following 1,4-addition on the catalytic residue ATG3, C264. To validate the contribution of electrostatic interaction to overall interaction, the salt concentrations should be varied in the LIR peptide-GABARAP/LC3 binding experiment (Figure 2e, f), where the effect of salt concentrations on ATG3 catalytic activity can be dismissed.

Minor points of critiques

(1) There is no label on L50 and K24 (Figure 3c, 3d).

(2) Lines should indicate H-bonds and salt bridges (Figure 3c, d).

(3) “derived by from” → “derived by” (a legend in Supplementary Figure 8)

(4) “CH3N” → “CH3CN” (SI page 14, 22)

(5) “NaCO3” → “NaHCO3” or “Na2CO3” (SI page 22)

Author's Response to Peer Review Comments:

Please see attached file

21 April 2023

Dear Editor,

Thank you handling this submission and for the extension to address the comments of the referees. We have now been able to conduct the requested additional experiments and made changes to the manuscript and the SI. Please find point-by-point responses below, and versions of the manuscript and SI with changes and additions highlighted in the manuscript files.

Reviewer(s)' Comments to Author:

Reviewer: 1

1. The binding affinities for peptides that included the novel LIR motif were measured by fluorescence polarization. However, the data in figures 2e,f have some issues. Overall, it would be good to display these data on a log-scale x-axis so data at lower concentrations can be seen more clearly. Also, the data points in Figure 2e and 2f do not appear to line up with the values stated in the methods section (this is most obvious in Figure 2e, but appears to be the case for 2f as well). Most importantly, the curve fits do not look like correct curve fits to extract Kd values from fraction-bound data. Specifically, Kd curves fit to the appropriate equation (for example, equation 5 in Dan Herschlag's excellent 2020 eLife resource https://elifesciences.org/articles/57264) are monotonic and do not decrease after saturating at high concentrations. However, some of these curves appear to decrease after saturating at high concentrations. These data should be double-checked and re-analyzed given these concerns.

Given these issues and the importance of measuring the affinity accurately, perhaps there is another assay (such as SPR, BLI, or AlphaLISA) that the authors could use to measure binding affinity to correlate their Kd values.

We thank the reviewer for pointing out poor data fit of our binding-affinity measurements. We have remeasured the affinities using fluorescence polarization. These show a better fit to determine the dissociation constants. Overall, the affinities in our initial submission have been confirmed. Figure 2 was updated with the new binding data and presentation on logarithmic scale as suggested by reviewer 1.

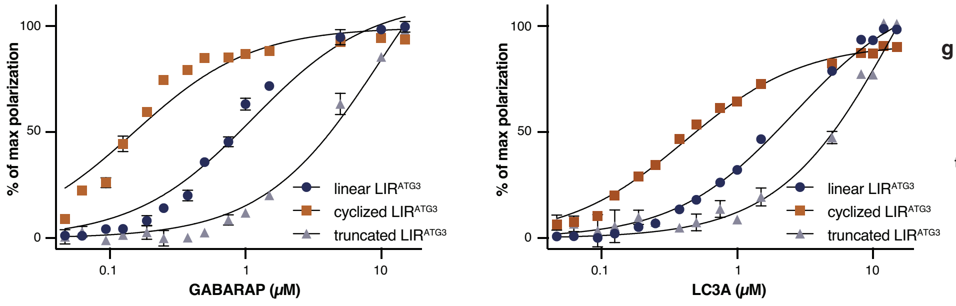

g

	GABARAP	LC3A
linear LIR^{ATG3}	1.2 ± 0.09 μM	2.6 ± 0.21 μM
cyclized LIR^{ATG3}	0.15 ± 0.01 μM	0.41 ± 0.03 μM
truncated LIR^{ATG3}	>10 μM	>15 μM

2. The authors state that "The stronger affinity of the cyclic peptides supports the importance of the β-sheet conformation in binding to LC3A and GABARAP". However, as their CD data show (Supplementary Figure 12), the cyclic peptide does not have a stable beta-sheet conformation in aqueous solution. It may be that there is some degree of pre-organization within the cyclic peptide, but there is no evidence for it in the manuscript. Later, the authors also write "We provide conclusive evidence that the bent β-sheet conformation for LIRATG3 is required for efficient binding to LC3/GABARAP and is not an artifact of co-crystallizing LIR peptide and LC3/GABARAP", but if this evidence is limited to the binding data with linear and cyclic peptides, then this evidence is not

even close to conclusive. These interpretations are complicated by the uncertainty surrounding the binding affinity data, since a peptide-protein complex with high micromolar affinity may crystallize (especially because GABARAP itself crystallizes relatively easily) but may have more flexible regions and nonspecific interactions that are prone to crystallization artifacts. If the affinity is indeed sub-micromolar, this may strengthen the case that the beta-sheet observed in the crystal structure is important for function. Even then, however, I don't think these data would comprise "conclusive evidence that the bent β-sheet conformation for LIRATG3 is required for efficient binding to LC3/GABARAP".

These interpretations should be omitted or additional evidence showing the importance of a beta-sheet conformation (rather than just an extended linear conformation) should be provided.

To confirm the importance of the beta-sheet conformation of LIR^{ATG3} we additionally prepared a truncated version of the LIR-peptide lacking amino acids 104-95. This peptide as shown above and in Figure 2 binds with >8-fold weaker affinity to both GABARAP and LC3A. This data clearly shows the importance of the solvent-exposed strand of the proposed beta-sheet. This data in combination with the binding as well as inhibitory data of the cyclized peptide strongly indicate the presence of a beta-sheet conformation of LIR^{ATG3}. If the peptide adopted other conformations such as an extended one, cyclization would lead prevent the peptide from adopting such an extended conformation which would be reflected in the binding affinity. However, as our cyclized peptides showed significantly tighter binding a beta-sheet conformation is the most logical explanation. It is our opinion that combining these data with our modelling and structural data provide conclusive evidence to the importance of the beta-sheet conformation of LIR^{ATG3}.

We have added the additional fluorescence polarization data to Figure 2.e,f and added the following additional explanatory statements in the text.

Tighter binding upon peptide cyclization strongly suggests that it is the β-sheet conformation, observed in our prediction, that binds to GABARAP/LC3A because conformational restriction of the peptide precludes binding to other regions of GABARAP in an extended fashion. A truncated peptide, containing amino acids G103-Y111 including the core LIR motif, bound 6-fold weaker than the linear peptide. The severely diminished affinity observed for the truncated peptide indicates that the solvent-exposed upper half of the β-sheet (104-95) contributes significantly to the binding of LIR^{ATG3}.

On a related point, the alanine scanning data indicate that W107 and V108 are important for ATG3-GABARAP cross-linking (and, by inference, ATG3-GABARAP binding). This is consistent with the crystal structure. However, the alanine scanning data also indicate that D102, I97, and E95 are at least as important as V108, but the specific importance of these residues do not appear to be rationalized by the crystal structure. This provides additional uncertainty that the crystal structure may not be showing the complete set of interactions required for the observed binding, and may imply that the actual binding motif involves a different conformation, or multiple different conformations.

As outlined above we showed that the upper section of the beta-sheet contributes significantly to the binding affinity of LIR^{ATG3} as also shown by our alanine screen. We believe that residues such as E95 and I97 are crucial for formation of the beta-sheet. E95 forms a salt-bridge with GABARAP R67 and I97 clamps down on V106 burying it in a hydrophobic pocket. We have the following added statements to the text to expand on these points.

ATG3 V108 is buried by hydrophobic interactions with GABARAP K46 and Y49 and the upper strand of the LIR β-sheet (E95, I97). These interactions block any solvent contact of V108 and likely strengthen its hydrophobic packing.

We agree that it is possible for the LIR motif to potentially adopt multiple conformations, however, based on the data presented above we believe that the beta-sheet conformation is the LIR^{ATG3} conformation adopted by this peptide when bound to GABARAP/LC3A.

3. The cellular work includes diligently performed ATG3 knockout experiments and complementation experiments with ATG3 variants. The results of these experiments, and the results of work with GABARAP-derived probe 2, suggest that the delta-LIR construct of ATG3 is impaired in its ability to mediate LC3/GABARAP lipidation. However, the results with a pulse-chase assay looking at ATG3-GABARAP complex formation shows impairment, but not abolishment, of the functional interaction. The authors do a good job trying to explain these nuances in the discussion. However, the model shown in figure 5f and the discussion of possible mechanisms are highly speculative. Ultimately, while the data do support a role for this noncanonical LIR motif, a clean model for how this portion of the flexible region of ATG3 affects activity of the lipidation complex remains elusive.

We agree with the reviewer that our findings do not point to a discrete function of LIR^{ATG3} in ATG3's enzymatic function. However, we believe that our findings will be instrumental in further elucidating the mechanism of GABARAP/LC3 lipidation by providing new data on one of the core enzymes of autophagy that was previously not unknown. Mechanistic work required to elucidate the precise function of this LIR motif would be beyond the scope of this publication and will require additional work.

Additional Questions:
Quality of experimental data, technical rigor: Top 5%

Significance to chemistry researchers in this and related fields: Moderate

Broad interest to other researchers: Moderate

Novelty: High

Is this research study suitable for media coverage or a First Reactions (a News & Views piece in the journal)?: No

Reviewer: 2

Recommendation: Publish in ACS Central Science after minor revisions noted.

Comments:
Summary of this manuscript
J. Farnung et al. prepared GABARAP/LC3 ABPs using the hydrazide acylation protocol developed by the Bode group. These ABPs enabled the identification of covalently linked GABARAP/LCL3-ATG3 complexes. The formation of a crosslinking complex is more reliable evidence compared to the results of the binding assay (Sci. Adv. 2021, 7, 40). The structural prediction by ColabFold has suggested a non-canonical β-sheet LIR motif of ATG3, and a co-crystal structure of the GABARAP-LIR peptide complex clearly showed the new LIR motif. Most researchers had missed an important motif consisting of 'WXXT' sequences for many years before this study. Since the scope of LIR motifs has been expanded, the discovery of new LIR and proteins involved in autophagy is expected. The combination of GABARAP/LC3 ABPs and ColabFold prediction enabled them for this novel discovery. Furthermore, the importance of this non-canonical LIR motif in LC3 lipidation is confirmed in cellulo experiments. These experimental results support the novelty of the discovery.

Major points of critiques
(1) Salt-dependent cross-linking efficiency was discussed (page 13, line 32, Supplementary Figure

13), but the crosslinking needed two steps, LIR-GABARAP interaction and the following 1,4-addition on the catalytic residue ATG3, C264. To validate the contribution of electrostatic interaction to overall interaction, the salt concentrations should be varied in the LIR peptide-GABARAP/LC3 binding experiment (Figure 2e, f), where the effect of salt concentrations on ATG3 catalytic activity can be dismissed.

We thank the reviewer for this suggestion. We measured fluorescence polarization of the cyclized peptide used in Fig. 2e in the presence of various concentrations of NaCl. As seen for the cross-linking assay, binding of the LIR peptide to ATG3 measured by fluorescence polarization is drastically influenced by the buffer's salt concentration, underlining the contribution of electrostatic interactions to the binding of LIR^{ATG3} to GABARAP. We have added an additional panel to Fig. S13 and an additional statement to the main text.

The hairpin turn binds mainly via electrostatic interactions to GABARAP. Increasing the salt concentration disrupts this interaction as shown by reduced fluorescence polarization of LIR^{ATG3} peptide upon binding to GABARAP, and cross-linking with ATG3 (Figure S13). These data highlight how binding of LIR^{ATG3} to GABARAP/LC3A is driven by a combination of hydrophobic and ionic interactions.

Minor points of critiques
(1) There is no label on L50 and K24 (Figure 3c, 3d).
(2) Lines should indicate H-bonds and salt bridges (Figure 3c, d).
(3) "derived by from" → "derived by" (a legend in Supplementary Figure 8)
(4) "CH3N" → "CH3CN" (SI page 14, 22)
(5) "NaCO3" → "NaHCO3" or "Na2CO3" (SI page 22)

We thank the reviewer for these suggestions and catching these mistakes and have corrected them in the manuscript.

Additional Questions:
Quality of experimental data, technical rigor: Top 1%

Significance to chemistry researchers in this and related fields: Top 1%

Broad interest to other researchers: Top 1%

Novelty: Top 1%

Is this research study suitable for media coverage or a First Reactions (a News & Views piece in the journal)?: Yes
